# Morphomechanical Innovation Drives Explosive Seed Dispersal

**DOI:** 10.1016/j.cell.2016.05.002

**Published:** 2016-06-30

**Authors:** Hugo Hofhuis, Derek Moulton, Thomas Lessinnes, Anne-Lise Routier-Kierzkowska, Richard J. Bomphrey, Gabriella Mosca, Hagen Reinhardt, Penny Sarchet, Xiangchao Gan, Miltos Tsiantis, Yiannis Ventikos, Simon Walker, Alain Goriely, Richard Smith, Angela Hay

**Affiliations:** 1 Max Planck Institute for Plant Breeding Research, Carl-von-Linné-Weg 10, 50829 Köln, Germany; 2 Mathematical Institute, University of Oxford, Radcliffe Observatory Quarter, Woodstock Road, Oxford OX2 6GG, UK; 3 Structure and Motion Laboratory, Department of Comparative Biomedical Sciences, Royal Veterinary College, University of London, Hawkshead Lane, Hatfield AL9 7TA, UK; 4 Institute of Plant Sciences, University of Bern, Altenbergrain 21, 3013 Bern, Switzerland; 5 Plant Sciences Department, University of Oxford, South Parks Road, Oxford OX1 3RB, UK; 6 Mechanical Engineering Department, University College London, Torrington Place, London WC1E 7JE, UK; 7 Zoology Department, University of Oxford, South Parks Road, Oxford OX1 3PS, UK

## Abstract

How mechanical and biological processes are coordinated across cells, tissues, and organs to produce complex traits is a key question in biology. *Cardamine hirsuta*, a relative of *Arabidopsis thaliana*, uses an explosive mechanism to disperse its seeds. We show that this trait evolved through morphomechanical innovations at different spatial scales. At the organ scale, tension within the fruit wall generates the elastic energy required for explosion. This tension is produced by differential contraction of fruit wall tissues through an active mechanism involving turgor pressure, cell geometry, and wall properties of the epidermis. Explosive release of this tension is controlled at the cellular scale by asymmetric lignin deposition within endocarp *b* cells—a striking pattern that is strictly associated with explosive pod shatter across the Brassicaceae plant family. By bridging these different scales, we present an integrated mechanism for explosive seed dispersal that links evolutionary novelty with complex trait innovation.

**Video Abstract:**

## Highlights

• Fruits explode while turgid, not dry, due to active contraction of epidermal cells
• Asymmetric deposition of lignin in endocarp *b* cells drives explosive energy release
• Endocarp *b* asymmetry is an evolutionary novelty underlying explosive dispersal
• Explosive dispersal emerges from cell and tissue interactions in multi-scale model

## Graphical Abstract

**Figure:**
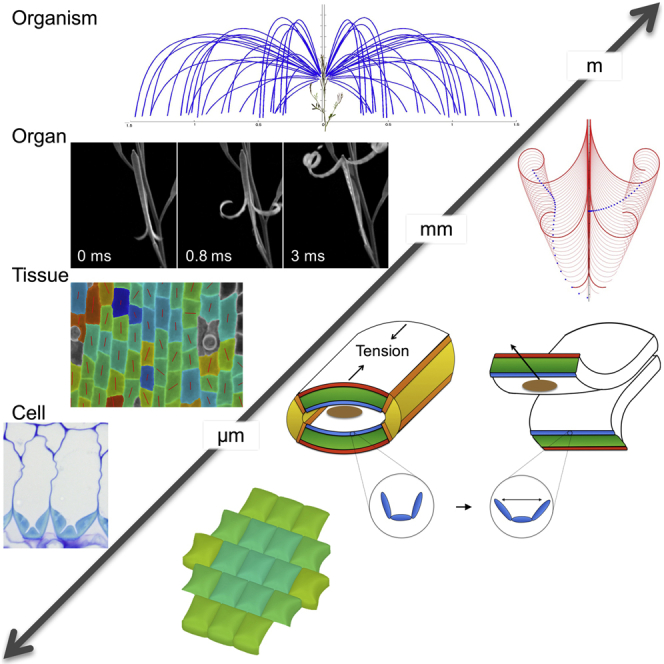

## Teaser

The violent explosion of seed pods is one of the fastest movements in the plant kingdom. Using interactions between cell and tissue-level processes, a multi-scale model reproduces the explosive seed dispersal in popping cress.

## Introduction

Understanding how morphological novelties evolved is a major goal of biology. Rapid plant movements, such as the “snap” of a Venus fly trap, are striking character gains that have led to trait innovations such as carnivory (Darwin, 1875). However, the majority of fast motions in plants and fungi are adaptations for dispersal. Catapulted pollen or synchronous puffs of fungal spores are evolutionary solutions to the problem drag poses to getting small particles airborne (Edwards et al., 2005, Roper et al., 2010). While the mechanics of these rapid movements are well described, little is known about the cellular basis of such novel phenotypes and how they have evolved.

Although plants are sessile, they can move by swelling, shrinking, or growing; for example, surface stomata open and close and leaves move with a circadian rhythm (Hoshizaki and Hamner, 1964, Schroeder et al., 1984). These movements are water-driven and are constrained by the timescale of water transport through cells and tissues (Skotheim and Mahadevan, 2005). To overcome this constraint and generate rapid motion requires a mechanism that stores elastic energy gradually but releases it rapidly. Such physical mechanisms can be diverse and fascinating: for example, the snap-buckling of a Venus flytrap or the cavitation catapult of a fern sporangium (Forterre et al., 2005, Noblin et al., 2012), but the biological processes by which they are produced are unknown. A key problem is that rapid movements are relatively rare and model species where the experimental tools for detailed functional studies exist, such as *Arabidopsis thaliana*, do not exhibit such movements. A fundamental theoretical challenge is that rapid movements are the culmination of activities integrated across different spatial scales, hence a complete understanding requires biomechanical models that link causal events at the cell and tissue levels to the macroscopic organ and plant response. To address these issues, we used experimental and theoretical approaches to analyze explosive seed dispersal in *Cardamine hirsuta*, a close relative of *A. thaliana*, commonly described as popping cress for the explosive shatter of its fruit pods (Hay et al., 2014, Rich, 1991). We took advantage of the genetic tractability of *C. hirsuta* (Barkoulas et al., 2008, Hay and Tsiantis, 2006, Vlad et al., 2014) combined with biophysical experiments, high-speed videography, quantitative imaging, and multi-scale mathematical modeling, in order to investigate and fully explain the biological and physical basis of explosive seed dispersal.

Explosive seed dispersal is a rapid movement found in various flowering plants and was likely a key innovation for the invasiveness of species such as *C. hirsuta*, *Impatiens glandulifera*, and the dynamite tree, *Hura crepitans* (Clements et al., 2008, Deegan, 2012, Randall, 2002, Swaine and Beer, 1977, Vogel, 2005, Yatsu et al., 2003). Seed launch speeds have been previously calculated using a variety of techniques including advanced high-speed cameras, which were used to record mean speeds ranging from 1–6 ms^−1^ (Deegan, 2012, Garrison et al., 2000, Hayashi et al., 2009, Hayashi et al., 2010). Seed dispersal occurs via a process called pod shatter in both the explosive fruit of *C. hirsuta* and the non-explosive fruit of *A. thaliana* and relies on the precise patterning of fruit tissues (Liljegren et al., 2004). Fruits of these species look very similar, with a fruit wall comprised of two valves that enclose the seeds, attached to the replum. The fruit splits open by dehiscence along a thin line of specialized tissues at the valve margins to allow pod shatter (Dinneny and Yanofsky, 2005). In *A. thaliana*, this occurs as the fruit dries out, separating the valves from the replum and exposing the seeds for dispersal. However, in *C. hirsuta*, explosive pod shatter occurs while the fruit is turgid, not dry (Schneider, 1935). This observation contradicts the established view that drying generates the energy for explosive seed dispersal by causing fruit tissues to deform (Beer and Swaine, 1977, Vaughn et al., 2011) and suggests that the *C. hirsuta* fruit uses a previously undescribed mechanism to generate tension actively.

Here, we uncover this mechanism through a comprehensive experimental and theoretical study of explosive seed dispersal in *C. hirsuta*. By combining analyses at different scales of magnitude, we identify specific cellular features that cause the tissue-level mechanics underpinning explosive dispersal. We demonstrate that tension is actively generated in *C. hirsuta* fruit by the anisotropic deformation of living cells that sustain turgor pressure. This unusual mechanism relies on a combination of three-dimensional cellular geometry and anisotropic cell wall properties of the fruit epidermis. Moreover, we show that the stored potential energy giving rise to tissue tension is released explosively via coiling of the fruit valves. This coiling mechanism requires the asymmetric localization of lignin in a single cell layer of the valve and represents an evolutionary novelty associated with explosive seed dispersal across the genus *Cardamine*.

## Results and Discussion

### Seed Dispersal Dynamics

**Figure 1:**
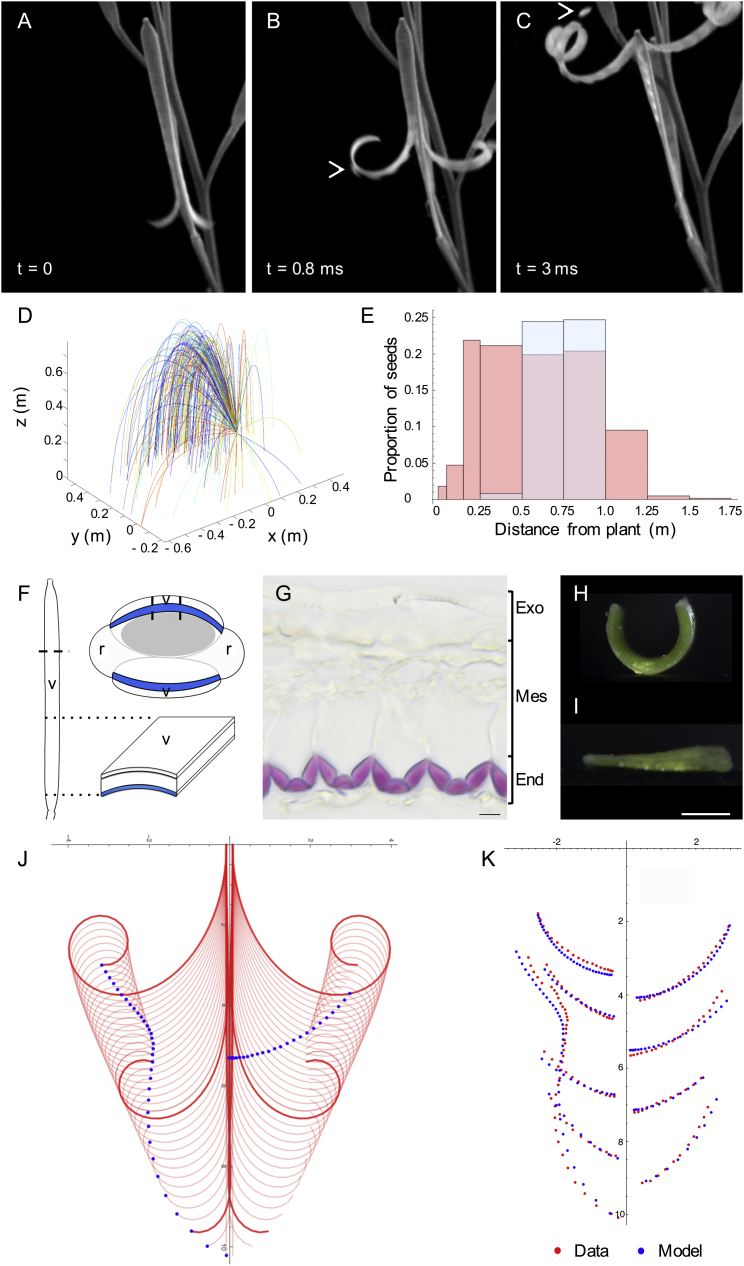
Dynamic Model of Explosive Seed Dispersal in *C. hirsuta* (A–C) Explosive seed dispersal recorded at 15,000 fps: the two valves detach from the fruit (A), curl back with seeds adhered to the inner valve surface (B), and launch seeds while coiling (C); t, time between frames; arrows indicate seeds. (D) Seed flight paths extrapolated from measured launch conditions; n = 229 seeds from 14 fruits; velocity max: 10.4 ms^−1^, mean: 5.0 ± 2.1 ms^−1^. (E) Measured distribution of 52,585 seeds dispersed by 21 plants (red) overlaid with computed distribution of seeds ejected from a single valve using model dynamics (blue). (F) Cartoon of *C. hirsuta* fruit, dashed line indicates transverse cut shown in adjacent cartoon, dashed lines through valve demarcate section shown in (G); dotted lines indicate longitudinal segment of valve shown in (H) and (I). v, valve; r, replum; endocarp *b* layer, blue; seed, gray. (G) Transverse valve section labeled as a mechanical trilayer; lignified endocarp *b* secondary cell walls (End) stain pink with phloroglucinol; non-lignified cells form two layers, exocarp (Exo) and mesocarp/non-lignified endocarp *b* (Mes). Scale bar, 10 μm. (H and I) Valve segments in water, intact (H) or lacking endocarp *b* layer (I). Scale bar, 1 mm. (J) Simulated trajectories of coiling valves from model. Valves shown at successive time intervals (red); valve tip and midpoint are marked (blue) to visualize how their position changes over time. (K) Trajectories at nine points on the valves quantified from high-speed movies (red); and simulated from the model at equivalent time steps (blue). Axes in (J) and (K) show distance (mm). See also Figure S1 and Movies S1 and S2.

**Figure S1:**
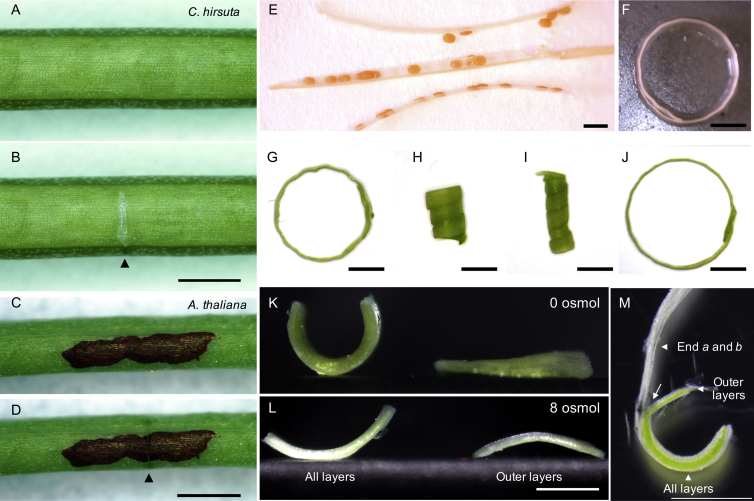
Mechanical and Osmotic Experiments in *C. hirsuta and A. thaliana* Fruits, Related to Figure 1 (A and B) Incision of the *C. hirsuta* valve shows mechanical tension in situ. Mature fruit before (A) and after a shallow cut is made in the valve outer layers (B). The cut layers immediately gape in the long direction of the fruit, revealing the white, lignified endocarp *b* cell walls underneath (arrowhead), indicating that the valve was in a state of tension while attached to the fruit. (C and D) The same experiment in *A. thaliana* shows no tension. Ink was applied to the intact valve (C) to aid visibility of the incision (D, arrowhead), which did not gape. (E–J) Turgor pressure in living cells is required for full valve curvature. Valves of alcohol-dried fruits fall off without explosion and are almost flat (E). Re-hydration in water produces little curvature in alcohol-treated valves (F) or in valves killed by freezing (G), as observed previously upon re-hydration of oven-dried *C. parviflora* fruits (Hayashi et al., 2010). Valves of living, freshly exploded fruits are curled much more tightly in coils of ∼2 mm diameter (H). Hydration of these same valves in water results in even tighter curling in coils of ∼1 mm diameter (I), while after plasmolysis in 4M salt solution the coils open to ∼5-6 mm diameter (J). Thus, the tight coiling of valves in explosive fruits (H) is an active process requiring living cells that can sustain internal pressure. In contrast to this, the slight residual curvature in dead, hydrated valves (F and G) is passive and could be explained by gradients of cell wall composition within the valve (Hayashi et al., 2010, Vaughn et al., 2011). (K and L) Both inner and outer layers of the valve and turgor pressure are required for curvature. In pure water (K), a valve segment comprising all layers (left) curves, while a segment of excised outer layers (right) remains flat. Please note that these data are presented in Figure 1 of the main text but are also shown here for clarity. In 8 osmoles of salt solution (L), curvature of the same intact valve segment (left) is very reduced, while the same segment of excised outer layers (right) curves slightly in the opposite direction, indicating that the outermost exocarp layer increases in length following plasmolysis. (M) Endocarp layers alone do not curve in water, as shown by separating the outer layers of a valve segment from the endocarp *a* and *b* layers (white arrow). The valve segment comprising all layers curves, while each of the separated layers remain flat. Therefore, a bilayer composed of the inner and outer valve layers is necessary and sufficient for curvature, rather than a bilayer composed of the endocarp *a* and *b* layers as previously proposed (Hayashi et al., 2010). Scale bars: 1 mm (A-D, K-M), 2 mm (E-J).

To quantify explosive seed dispersal in *C. hirsuta* at the plant and organ level, we recorded the shatter of fruit pods using high-speed videography, extrapolated the trajectories of launched seeds, and measured the distribution of seeds dispersed around parent plants. During explosive pod shatter the two valves curl back from the fruit pod, initially peeling the seeds off the inner septum and launching them at speeds in excess of 10 ms^−1^ (Figures 1A–1C; Movie S1). This process is rapid, taking less than 3 ms, and fires the small seeds upon ballistic trajectories to land within a 2-m radius of the parent plant (Figures 1D and 1E). The exploded valves come to rest in a curled configuration of three or four coils (Figure 1C). We identified key properties of the valve associated with explosive pod shatter by comparing the valves of non-explosive *A. thaliana* and explosive *C. hirsuta* fruit. Two striking features differentiated these fruit. First, *C. hirsuta* valves contain more lignin, localized asymmetrically to cell walls on the inner side of the endocarp *b* layer (Figures 1F and 1G) (Vaughn et al., 2011). Lignin is a complex phenylpropanoid polymer that adds stiffness to secondary cell walls, suggesting that this inner valve layer is considerably stiffer in the explosive fruit of *C. hirsuta*. Second, shallow incisions to the outside of the turgid valve caused wounds that gaped instantly in *C. hirsuta* but not in *A. thaliana* (Figures S1A–S1D). This observation implies that, in *C. hirsuta*, the outer tissue layer is under tension while the valve is flat, prior to explosion.

To examine the mechanical properties of different tissues within the *C. hirsuta* valve, we performed simple dissections. A valve curves lengthwise both in water (Figure 1H) and in air (Figures 1A–1C) when released from the fruit. However, when we separate the lignified tissue from the rest of the valve, this curvature vanishes (Figures 1I and S1M). Moreover, when we separate the outer valve tissues from the inner lignified layer, the outer layer shortens while the lignified layer does not (Table S1). Based on these findings, we considered the *C. hirsuta* valve as three mechanical layers: an active soft outer layer, a passive middle layer, and a stiff inner layer. The exocarp (active outer layer) is attached to the inextensible secondary cell wall of the endocarp *b* (stiff inner layer) through the mesocarp and the non-lignified part of the endocarp *b* (middle layer), which act as a passive buffer (Figure 1G). Therefore, a shortening of the exocarp, while the stiff endocarp *b* conserves its length, causes the valve to naturally coil when released from the fruit.

These observations suggest a mathematical model for the whole valve based on three elastic layers, each with a different reference geometry, attached together so that, in the flat state, the outer layer is in tension and the other two are in compression. The elastic energy stored in this trilayer is determined by the deformation of underlying tissues and can be expressed as a function of the curvature along the length of the valve (an explicit energy description is given in Supplemental Experimental Procedures). The parameters required for the model consist of geometric parameters measured in live fruit and fresh sections (Supplemental Experimental Procedures) and material parameters characterizing the stiffness of each layer. The Young’s modulus of lignin is readily available in the literature (Burgert and Dunlop, 2011) and characterizes the stiffness of the endocarp *b* layer. We obtained the tissue-level stiffness of the exocarp and middle layers from extensometer measurements of the valve (Supplemental Experimental Procedures). Initially, the trilayered valve is flat, storing elastic energy. Upon dehiscence, the valve is free to coil on itself, transforming elastic potential energy to kinetic energy. We described the dynamics of the coiling using classical mechanics and found that the model closely matched our observations of valve coiling (Figures 1A–1C and 1J; Movie S2; Supplemental Experimental Procedures). We validated the spatio-temporal accuracy of this model by directly comparing model simulations with measured trajectories of distinct points along the valve tracked from high-speed movies of explosive pod shatter (Figure 1K). The striking agreement we observed between model and data confirms that the dynamics of explosive pod shatter are captured correctly and suggests that this tissue-scale model should be predictive of seed dispersal at the plant scale.

To test this hypothesis, we used the model dynamics of a single valve to obtain the ballistic trajectories of seeds explosively dispersed away from the parent plant. Taking from the model the initial velocity of each seed catapulted from the valve and a spatial orientation of the valve on the plant, we computed the motion and probability distribution of multiple seeds through Monte Carlo simulations (Supplemental Experimental Procedures). In high speed movies we observed a transient adhesion between seeds and valve (Figure 1B, arrow), which we modeled via a linear viscoelastic force provided by a pectic cell surface. This means that the force of attachment depends linearly on both time and the distance between seed and valve. When the distance between seed and valve exceeds a critical length, this attachment breaks and the seed is released to follow a ballistic trajectory under the influence of aerodynamic drag. Comparing seed distributions and seed launch angles between theoretical predictions and direct measurements, we found the assumption of viscoelastic adhesion to be the only mechanism consistent with the data (Figure 1E; Supplemental Experimental Procedures). We also found that the distance of dispersed seeds had a plateau in its distribution (Figure 1E) (Schneider, 1935), suggesting that this launch mechanism appears tuned to spread seeds over a maximal area, rather than to achieve a maximal distance.

### Endocarp *b* Deletion Mutant Is Non-explosive

**Figure 2:**
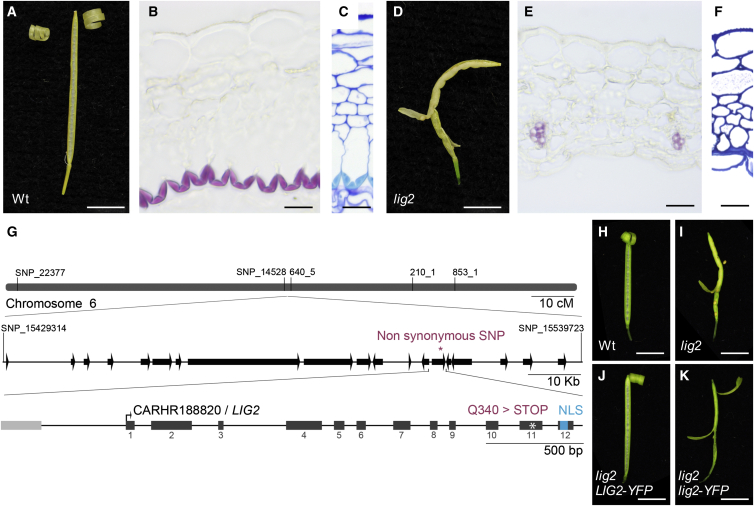
Loss of the Endocarp *b* Layer in *lig2* Prevents Explosive Pod Shatter (A–F) Exploded fruit observed in air and transverse valve sections through mature stage 17 fruit of wild-type (A–C) and *lig2* (D–F). Lignified cell walls stain pink with phloroglucinol (B and E) and cyan with TBO (C and F). Note vascular bundles contain lignified xylem cells. Wild-type valves have 9.2 ± 0.1 cell layers mid-valve and *lig2* valves have 8.2 ± 0.1, n = 36 valves, data represented as mean ± SEM. Scale bars, 5 mm (A and D), 20 μm (B, C, E, and F). (G) Cartoon of *C. hirsuta* chromosome 6 region containing *LIG2*. Name and position of five markers used for mapping are indicated above chromosome; scale bar, 10 cM. Zoomed-in region flanked by two additional markers contains 19 predicted genes (arrows) and a single non-synonymous SNP (^∗^); scale bar, 10 kb. Zoomed-in CARHR188820/*LIG2* locus containing a C2523 > T mutation in exon 11 that causes a Q340 > STOP mutation (^∗^) before the NLS at amino acids 367–383, *LIG2* exons are shown as dark gray boxes, non-coding sequences as lines, and the START codon is indicated by an arrow, upstream gene CAHR188810 is shown as a light gray box. Scale bar, 500 bp. (H–K) Mature fruits of wild-type (H), *lig2* (I), *lig2* complemented with a fluorescently tagged genomic *LIG2* construct, *LIG2-YFP* (J), and not complemented with a fluorescently tagged mutant *lig2* construct, *lig2-YFP* (K). Scale bars, 5 mm. See also Figure S2.

**Figure S2:**
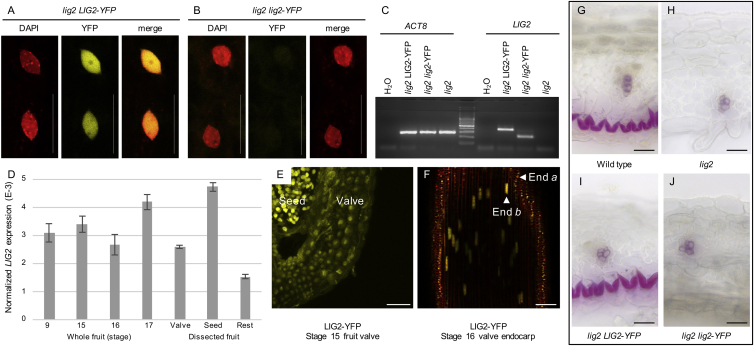
The *lig2* Mutation Prevents Nuclear Accumulation of the DNA Binding Protein LIG2, Related to Figure 2 (A and B) CLSM of DAPI-stained fruit mesocarp cells from *lig2; LIG2-YFP* (A) and *lig2; lig2-YFP* (B) transgenic lines. DAPI signal (red) indicates the nucleus, YFP signal (yellow) accumulates in the nucleus in *lig2; LIG2-YFP* (A) cells, but is extremely reduced in *lig2; lig2-YFP* (B) cells, and the merged DAPI and YFP signals confirms the nuclear localization of YFP in (A). (C) RT-PCR performed on cDNA template reverse transcribed from RNA samples of *lig2, lig2 LIG2-YFP* and *lig2 lig2-YFP* transgenic lines. *LIG2* and YFP primers were used to amplify a 402 bp product from the *LIG2-YFP* transgene and a 224 bp product from the *lig2-YFP* transgene. 402 bp and 224 bp amplicons in these samples indicated that both transgenes were expressed. No amplification was observed in the *lig2* sample. Amplicons of the *ACT8* housekeeping gene indicated equal amounts of cDNA template in each RT-PCR reaction. (D) Expression levels of *LIG2*, measured by qRT-PCR, are low throughout fruit development with no significant differences between stage 9 and other stages (Student’s t test p > 0.05). *LIG2* is expressed in all tissues of stage 17 fruit with significantly higher expression in the seed than the valve and significantly higher expression in the valve than the rest of the fruit (Student’s t test p < 0.01). Mean values and standard deviations are shown. *LIG2* expression was normalized to expression of the reference gene *Clathrin/AP2M* (CARHR174880). (E and F) CLSM of LIG2-YFP expression (yellow). Nuclear expression is observed in the seed and all layers of the valve in a cross section of stage 15 fruit (E). Nuclear expression is observed in the endocarp *b* and *a* layers in an *en face* section of a stage 16 valve; cells are outlined by propidium iodide staining (F). (G–J) Transverse valve sections, 70 μm thick, stained with phloroglucinol to visualize lignin. A lignified endocarp *b* cell layer is present in wild-type (G), absent in *lig2* (H), restored when a *LIG2-YFP* transgene is introduced into the *lig2* mutant (I) but not when a *lig2-YFP* transgene containing the *lig2* mutation is introduced into the *lig2* mutant (J). Scale bars: 25 μm (A, B, E), 50 μm (F), 20 μm (G-J).

To investigate whether the endocarp *b* layer is strictly required for explosive pod shatter, we took a genetic approach. Having shown that this stiff layer plays a mechanical role in generating valve curvature, we reasoned that an endocarp *b* deletion mutant should reveal how important this layer is for explosive shatter. This class of mutant had not been previously identified in *A. thaliana*, so rather than follow a targeted genome editing approach we conducted a mutant screen. We screened a population of ethyl methanesulfonate (EMS)-treated *C. hirsuta* plants for mutants with less lignified valves. In one such mutant, *less lignin2* (*lig2*), the entire endocarp *b* cell layer was missing (Figures 2A–2F). We showed that *lig2* is a loss-of-function mutant caused by a premature stop codon before the nuclear localization signal in the *C. hirsuta* ortholog of the DNA-binding protein BRASSINOSTEROID-INSENSITIVE4 (At5g24630; Figures 2G–2K) (Breuer et al., 2007, Kirik et al., 2007). *LIG2* is expressed in endocarp *b* cells and throughout the fruit, and the *lig2* mutation prevents nuclear accumulation of LIG2, resulting in loss of endocarp *b* layer integrity through mechanisms that remain to be determined (Figure S2). Importantly, pod shatter in the *lig2* mutant was non-explosive (Figure 2D), providing genetic evidence that the endocarp *b* layer is indeed necessary for explosive pod shatter.

### Secondary Cell Wall Geometry Enables Explosive Energy Release

**Figure 3:**
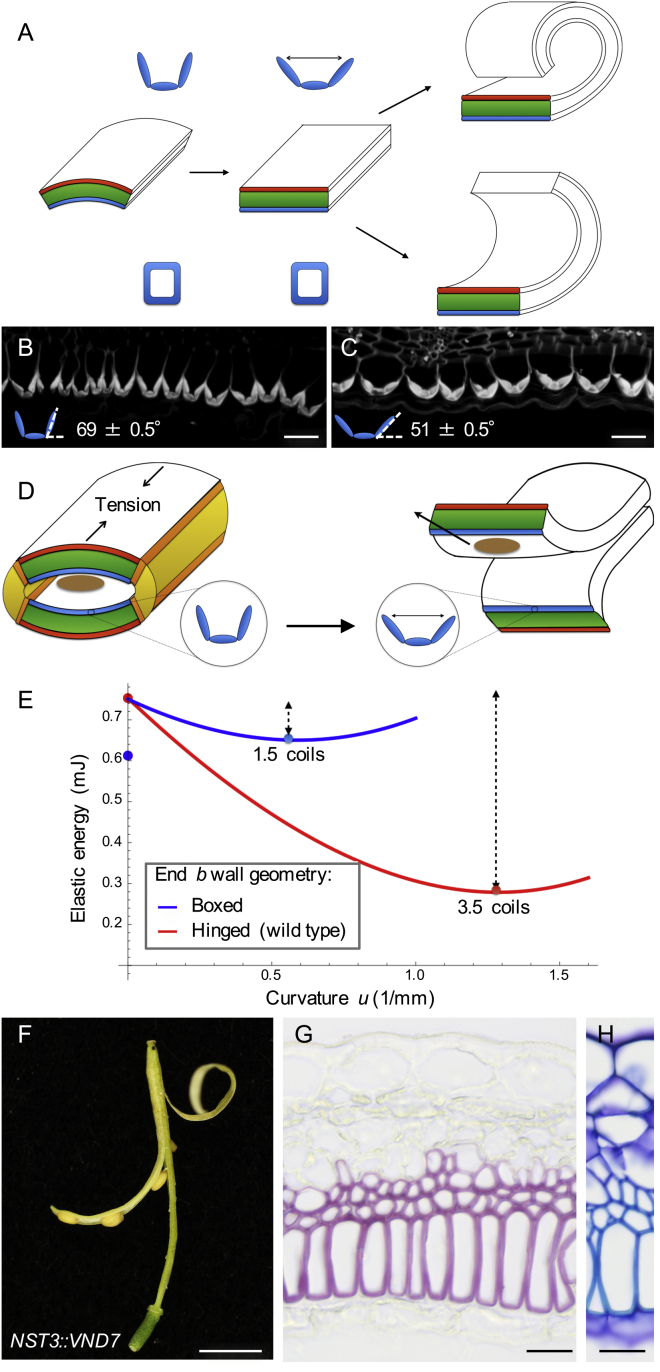
Lignified Cell Wall Geometry Triggers Explosive Energy Release (A) Cartoon of valve geometry specified in model, exocarp (red), middle layers (green), lignified endocarp *b* (blue), for wild-type (hinged), and boxed endocarp *b* cell wall. (B and C) Lignin autofluorescence in endocarp *b* cell walls pre- (B) and post-explosion (C); cartoons show hinge angle, n = 659 cells, data represented as mean ± SEM. Scale bars, 20 μm. (D) Cartoon of how the endocarp *b* hinge mechanism triggers energy release. Left panel: valves are curved in cross section and building tension while attached to the fruit. Dehiscence zones (orange) form along the valve margins, weakening this attachment. Right panel: valves flatten in cross section via opening of the lignified endocarp *b* hinge (blue). Valves detach from the fruit as they coil to relieve tension, transferring kinetic energy to launch seeds (brown). Replum (yellow). (E) Energy profile of valve with hinged (red) or boxed (blue) endocarp *b* wall geometry modeled during explosive pod shatter. Energy computed once the valve cross-section is flat and plotted as a function of longitudinal curvature. For each case, the energy minimizer is shown as a point on the curve and the energy released as a dashed line; coils per valve are indicated for these points. Points on the y axis indicate initial energy when the valve cross section is curved; note that energy input is required to flatten the valve with boxed endocarp *b* wall geometry. (F–H) Fruit observed in air and transverse valve sections through mature *NST3::VND7* fruit (F). Boxed geometry of lignified endocarp *b* cells and two adjacent mesocarp layers stained pink (G) and cyan (H). Scale bars, 5 mm (F); 20 μm (G and H). See also Figure S3.

**Figure S3:**
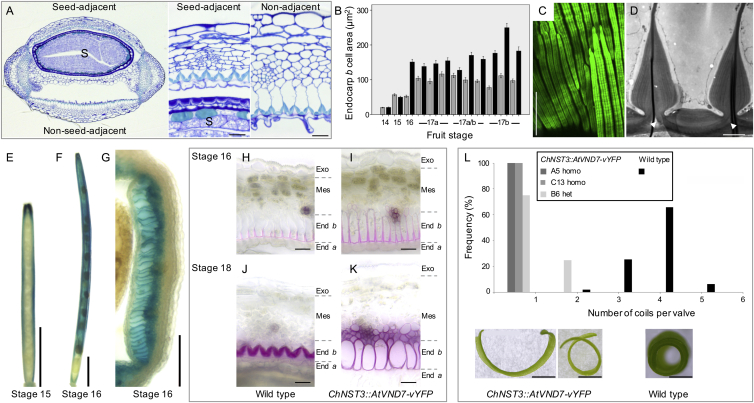
*C. hirsuta* Valve Geometry and Anatomy, Related to Figure 3 (A) Transverse sections through the fruit show the valve is bowed outward when adjacent to a seed and valve depth, especially that of the endocarp *b* cell layer, is much reduced when the valve is adjacent to a seed versus non-adjacent, suggesting that seed expansion may exert tension in the lateral direction across the valve (see Figure S6); S: seed. (B) Bar chart of mean cross-sectional area of endocarp *b* cells at positions in the valve immediately adjacent to a seed (gray) and non-adjacent to a seed (black). Sections were analyzed from every fruit along a single inflorescence stem at successive developmental stages (14 to 17b). From stage 16 onward, cell area is considerably reduced if positioned adjacent to a seed. Error bars show SEM. (C) CLSM *en face* view of the lignified secondary cell walls of the endocarp *b* cell layer showing lignin autofluorescence in three long rods per cell connected by thin hinges; cell length ∼2 mm. (D) Transmission electron micrograph transverse view of the lignified secondary cell walls of endocarp *b* cells showing thin hinges (arrowheads) connecting three rods on the cell face adjacent to the seeds. (E–G) *C. hirsuta NST3::GUS* fruits stained and viewed as whole mounts (E, F) or a 100 μm thick vibratome cross section (G). GUS expression is observed in cells of the replum but not the valve just prior to stage 16 (E), and in the valve from stage 16 onward (F) in the endocarp *b* cell layer and adjacent few mesocarp cell layers (G). (H–K) Timing of lignification in phloroglucinol-stained 70 μm thick vibratome cross sections of wild-type (H, J) and *ChNST3::AtVND7-vYFP* (I, K) valves. Lignin was first deposited in the endocarp *b* cell layer at stage 16 in both wild-type (H) and *ChNST3::AtVND7-vYFP* (I). At stage 18, the final pattern of lignification differed dramatically between the endocarp *b* cell layer in wild-type (J) and the endocarp *b* and adjacent few mesocarp cell layers in *ChNST3::AtVND7-vYFP* (K). (L) Distribution of the number of complete coils per valve in water for wild-type and 3 independent *ChNST3::AtVND7-vYFP* transgenic lines; homo: homozygous, het: heterozygous for transgene. Below the graph are shown representative *ChNST3::AtVND7-vYFP* and wild-type valves in water. Scale bars: 20 μm (A), 100 μm (C, G), 2 μm (D), 2 mm (E, F), 1 mm (L).

We have shown that the lignified endocarp *b* layer is required for explosive pod shatter and has a mechanical role in generating valve curvature. However, explosive pod shatter also requires a means of rapid energy release. To identify this mechanism, we investigated the role of the fruit valve geometry and the geometry of the lignified secondary cell walls of the endocarp *b* layer. During fruit maturation, the growing seeds deform the valve, so that the valve cross-section is not flat but rather is bowed outward (Figures 3A, S3A, and S3B). In order to release valve tension by coiling lengthwise, the valve must first flatten in cross-section (Figure 3A). The same principle is in action in so-called “slap bracelets” (these bracelets are made out of a strip of metal with a curved cross section when the central axis is straight and a flat cross section when the central axis is coiled). For the fruit valve to deform from a curved to a flat cross-section, either the endocarp *b* layer must widen (Figure 3A, blue) or the exocarp layer must narrow (Figure 3A, red). We hypothesized that the geometry of the endocarp *b* secondary cell wall provides the key: lignin is deposited with subcellular precision to form three stiff rods connected by very thin hinges (Figures 2B and 2C; Figures S3C and S3D). We observed that these hinged cell walls open during explosion (Figures 3B and 3C), enabling the stiff endocarp *b* layer to widen passively at a negligible cost of mechanical energy (Figure 3A; Supplemental Experimental Procedures). Therefore, once sufficient tension is established along the length of the valve and the dehiscence zone weakens at the valve margins, this hinge mechanism allows the valve to change freely from a curved to a flat cross-section and release the tension by coiling (Figure 3D).

This mechanism for energy release can be quantified by modeling the energy landscape of the valve from the moment it detaches from the fruit (Figure 3E; Supplemental Experimental Procedures). For this analysis, we identified three valve configurations: the initial state with a curved cross-section, a transitory state with a flattened cross-section, and the final energy minimizing (equilibrium) state that is coiled lengthwise (Figure 3A). The energy in each valve configuration is the sum of the bending and stretching contributions due to the deformation of the three idealized tissue layers shown in Figure 3A. We found that the equilibrium configuration of the detached valve is a coiled structure with three to four coils, which matches well with experimental data for fully hydrated valves (Figures 3E and S3L). We also computed a drop in energy of ∼0.5 mJ from the initial valve configuration to the coiled state (Figure 3E). This energy, converted from elastic potential energy into kinetic energy, is what drives the explosive nature of pod shatter and seed dispersal in *C. hirsuta*. Our model suggests that the passive opening of the hinged secondary cell wall in the endocarp *b* layer during cross-sectional flattening of the valve is of fundamental importance in explosive pod shatter. Without hinges, the valve transition from a curved to a flat cross-section would require energy input, which would considerably alter the energy landscape during pod shatter. For example, the same computation using a “boxed” geometry for the lignified endocarp *b* cell wall results in a much smaller energy difference between the initial and the equilibrium states and fewer coils in the equilibrium state valve (Figures 3A and 3E; Supplemental Experimental Procedures). Notably, this boxed geometry is found in *A. thaliana* and Brassica crops (Spence et al., 1996), plausibly explaining why pod shatter is non-explosive in these related species.

### Hinged Secondary Cell Wall Geometry Is an Evolutionary Novelty

To test our hypothesis that the hinged geometry of endocarp *b* cells provides the key mechanism for explosive energy release, we employed transgenic and phylogenetic analyses. We modified secondary cell wall patterning in endocarp *b* cells to create a boxed geometry that cannot “open,” and assessed explosive pod shatter. To do this, we expressed the *A. thaliana VASCULAR-RELATED NAC-DOMAIN PROTEIN7* (*VND7*) gene, which induces secondary wall formation (Kubo et al., 2005), using the promoter of the *C. hirsuta NAC SECONDARY WALL THICKENING PROMOTING FACTOR3* (*NST3*) gene (Mitsuda et al., 2007). This transgene initiates lignification of the endocarp *b* at a similar stage to wild-type and subsequently lignifies two adjacent mesocarp cell layers but not the endocarp *a* (Figures S3E–S3K). In comparison to wild-type, the secondary wall of endocarp *b* cells in *NST3::VND7* lines was uniformly thickened and lignified, creating a stiff box around each cell (Figures 3G and 3H). This modified geometry prevented explosive pod shatter and dehisced valves formed only one coil, similar to our model predictions (Figures 3E, 3F, and S3L). Therefore, the hinged geometry of the endocarp *b* secondary cell wall is fundamental to the explosive release of energy stored in the valve.

**Figure 4:**
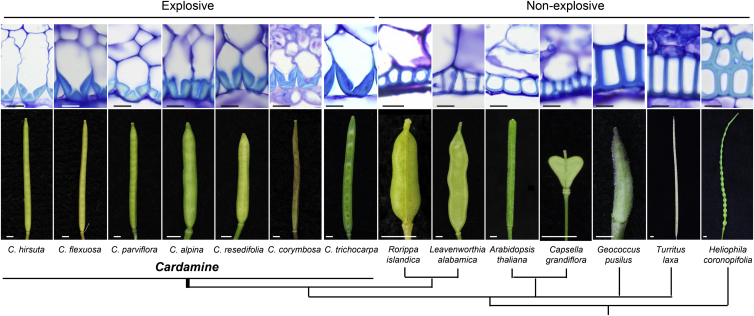
Morphomechanical Innovation Drives Explosive Seed Dispersal Endocarp *b* secondary cell wall geometry in representative species with explosive pod shatter in *Cardamine* and with non-explosive pod shatter in a Brassicaceae-wide sample. Lignified cell walls stain cyan with TBO in transverse valve sections through mature fruit; fruit morphology is shown for each species and their phylogenetic relationship is indicated by the cladogram below. Scale bars, 10 μm (cells); 2 mm (fruits). See also Figure S4.

**Figure S4:**
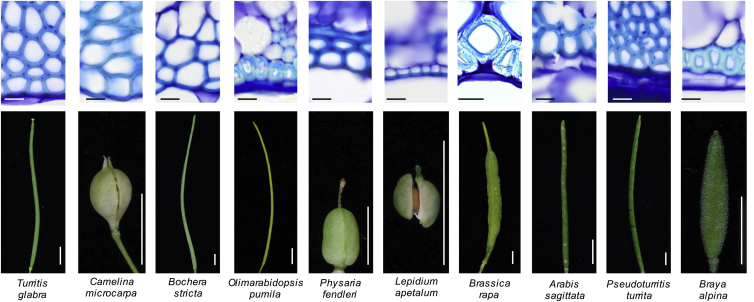
“Boxed” Geometry of Endocarp *b* Secondary Cell Wall in Brassicaceae Fruit with Non-explosive Pod Shatter, Related to Figure 4 Endocarp *b* secondary cell wall geometry in representative species with non-explosive pod shatter in the Brassicaceae family. Lignified cell walls stain cyan with TBO in transverse valve sections and fruit morphology is shown for each species. Scale bars: 1 mm (fruits), 10 μm (histology panels).

To test whether the hinged geometry found in the endocarp *b* secondary cell wall of *C. hirsuta* may represent a morphomechanical innovation associated with trait evolution, we analyzed this character across a broad sample of species in the Brassicaceae. To our knowledge, *Cardamine* is the only genus in this large plant family where explosive seed dispersal is found, and we observed a hinged secondary cell wall in the endocarp *b* layer of all *Cardamine* species that we sampled with explosive pod shatter (Figure 4). Conversely, we observed a boxed secondary cell wall in the endocarp *b* layer of a wide sample of species with non-explosive pod shatter (Figures 4 and S4). Together with this phylogenetic association, we have provided genetic evidence that the endocarp *b* cell layer, and specifically the geometry of its secondary cell wall, is necessary for explosive pod shatter. Additionally, we have provided a model that explicitly describes how this cell wall geometry enables explosive pod shatter. Therefore, we conclude that the hinged geometry of endocarp *b* secondary cell walls in *Cardamine* is an evolutionary novelty that allows valves to release elastic potential energy stored in the valve trilayer to drive ballistic seed dispersal.

### Turgor-Driven Shrinkage

**Figure 5:**
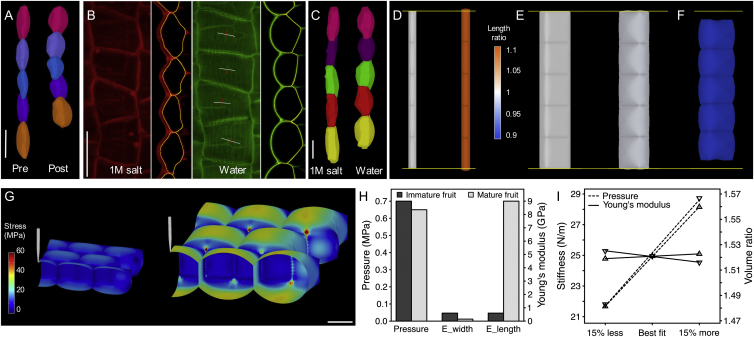
Turgor-Driven Shrinkage (A–C) Exocarp cells aligned to longitudinal fruit axis. (A) Side view of segmented cells from CLSM stacks of propidium iodine (PI)-stained fruits pre- and post-explosion, in water. (B) Top and side view of PI-stained cells treated with 1 M salt or water prior to imaging, cell outlines in yellow were used for quantitation and crosshairs show principal directions of shrinkage (red) and expansion (white). (C) Side view of cells segmented from CLSM stacks of PI-stained short valve segments treated with 1 M salt or water prior to imaging. Scale bars, 50 μm (A, B), 20 μm (C). (D–F) FEM simulations of cells pressurized from 0 Mpa (left) to 0.7 MPa (right); heatmap shows relative increase (orange) or decrease (blue) in cell length; horizontal yellow line shows initial length. Cell dimensions: 100 × 20 × 20 μm for *A. thaliana* exocarp cells (D), 50 × 50 × 20 μm for *C. hirsuta* exocarp cells (E and F). Cell wall material: isotropic (D and E), anisotropic (F). Pressure: 0 MPa (left, D and E), 0.7 MPa (right, D–F). (G) FEM simulations of exocarp cells in immature fruit of cell dimensions 30 × 20 × 14 μm (left) and mature fruit of cell dimensions 50 × 50 × 20 μm (right), micro-indented by a CFM tip. Heatmap shows stress in MPa. Scale bar, 20 μm. (H) Barplot of turgor pressure and cell wall elasticity parameters given by the FEM model for immature (dark gray) and mature (light gray) exocarp cells shown in (G). Young’s modulus in the width (E_width_) and length (E_length_) directions of the cell wall, defined by the fruit’s principal axes. (I) Sensitivity analysis of FEM model. Effect of best-fit parameters and values 15% lower and higher for pressure (dashed lines) and the Young’s modulus ratio (E_width_:E_length_, solid lines) on cell stiffness (N/m) and cell volume (ratio change), shown on the left and right y axes, respectively. See also Table S1 and Movies S3, S4, and S5.

We have identified the role of the endocarp *b* secondary cell wall in energy release; however, the other critical component for explosive pod shatter is the build-up of elastic energy in the system. To address this mechanism, we investigated the cellular basis for the differential shortening of the fruit valve that generates tension (Figures 1H and 1I). We measured a 20% reduction in cell length in the outermost exocarp layer between the flat valve, attached to the fruit, and the curled, detached valve (Figure 5A; Table S1). To understand the mechanics of this cell shortening, we challenged a previous proposal that shrinkage in the *C. hirsuta* valve is caused by passive loss of cell turgor pressure via drying (Vaughn et al., 2011). Under the “drying” hypothesis, detached valves would flatten out in pure water where cell turgor pressure is high due to osmosis. Yet, we observed higher curvature in water than in air, which then flattened out upon transfer to salt solution where the cells lost turgor (Figures S1H–S1J). Moreover, explosive shatter can be prevented by drying fruits with alcohol or freezing them (Figures S1E–S1G). These results show that drying is not the cause of exocarp cell shortening in *C. hirsuta* and suggest that exocarp shortening is an active process, requiring living cells that can sustain turgor pressure.

Turgor-induced shrinkage is counter-intuitive since turgor pressure drives plant cell expansion. To resolve this apparent contradiction, we compared the three-dimensional shape of exocarp cells at low turgor (1 M salt treatment) and high turgor (pure water). We found that exocarp cells in mature fruit responded to increased turgor by a 53% expansion in volume but a 12% shrinkage in length (along the fruit axis), accompanied by an expansion in depth (40%) and width (18%) (Figures 5B and 5C; Table S1). Therefore, as exocarp cells pump up, they shorten, analogous to artificial air muscles that contract when pressurized (Tondu, 2012). However, while the valve is attached to the fruit, these cells are prevented from contracting because the stiff endocarp *b* layer is inextensible, so the valve is held flat in a state of tension. In this state, the system is building elastic energy. When the valve is detached from the fruit, exocarp cells are free to relax toward their reference dimensions and hence, in the energy-minimizing state, the valve is coiled.

To understand how contraction in one direction can occur during overall volumetric expansion, we considered the stresses that develop in the cell wall during an increase in turgor and explored which cellular parameters were responsible for this behavior. To approach this problem, we constructed a finite-element model of pressurized cells (Bassel et al., 2014) and used this model to identify the parameters required to mimic the cell deformations measured in response to osmotic treatments (Figures 5C–5F; Supplemental Experimental Procedures). First, we used rectangular boxes made of isotropic material to model typical plant cells, such as exocarp cells in *A. thaliana* (100 × 20 × 20 μm, length × width × depth). When these cells were inflated with an internal pressure of 0.7 MPa, we found that the cells lengthened, as expected (Figure 5D; Movie S3). However, when we used cellular dimensions for the exocarp of *C. hirsuta* fruit that are competent to explode (50 × 50 × 20 μm), the cells shortened slightly (Figure 5E; Movie S4). The increased surface area of the relatively shallow cells caused the surface and bottom walls to bulge out, increasing the depth and shortening the length of cells. However, the shortening was negligible and insufficient to reproduce the change in geometry observed in osmotic treatments (Figure 5C; Table S1). Next, we hypothesized that an anisotropic cell wall material, which is stiffer in the longitudinal direction of the fruit, might cause the cells to shorten. Using the same cell template, we implemented anisotropic material properties for the cell wall in our model. We found that a significantly higher Young’s modulus in the longitudinal compared to the transverse direction resulted in 12% shrinkage in length when cells increased in volume by 53%, which matched the deformations of exocarp cells measured during osmotic experiments (Figures 5C and 5F; Table S1; Movie S5). These results suggest that both cell shape and cell wall anisotropy play an important role in turgor-driven shrinkage and predict that changes in cell geometry or wall material properties during fruit development may contribute to explosive pod shatter.

We then tested whether turgor pressure increased in the valve during development, since valve tension requires turgid cells. Although the absolute stiffness measured by cellular force microscopy (CFM) (Routier-Kierzkowska et al., 2012) was higher in the exocarp of mature rather than immature *C. hirsuta* fruits, we investigated whether this could simply be an effect of cell geometry (Figure 5G). This is because larger cells are expected to appear stiffer given the same turgor pressure (Weber et al., 2015). We performed finite element simulations of pressurized cells to model the exocarp of immature fruits, for which we assumed an isotropic cell wall material, as well as mature fruits with cell walls stiffer in the longitudinal direction (Supplemental Experimental Procedures). The cellular models could fit both the deformations measured during osmotic treatments and the stiffness data quantified by CFM. Mechanical parameters such as the Young’s moduli (E_width_ and E_length_) and turgor pressure extracted from the model fit (Figure 5H) are within the ranges previously reported for plant cells (Cosgrove, 2016, Kim et al., 2015, Pritchard et al., 1989, Wang et al., 2004, Weber et al., 2015). The value of the Young’s modulus for the stiffer direction (E_length_) is approximate, as the simulation is less sensitive to variation of this parameter (Figure 5I). In contrast, the model is very sensitive to variations in turgor pressure (Figure 5I), so the value for this parameter is more precise. Because we were able to fit the data for both immature and mature fruits without invoking any substantial change in turgor pressure as the fruit developed (Figure 5H), we conclude that the increase in apparent stiffness as the fruit matures is due to the changes in cell geometry rather than an increase in turgor pressure. This suggests that tension develops in the valve while the cell turgor pressure remains relatively constant at 0.65–0.7 MPa.

### Cellular Mechanics Play a Key Role in Turgor-Driven Shrinkage

**Figure 6:**
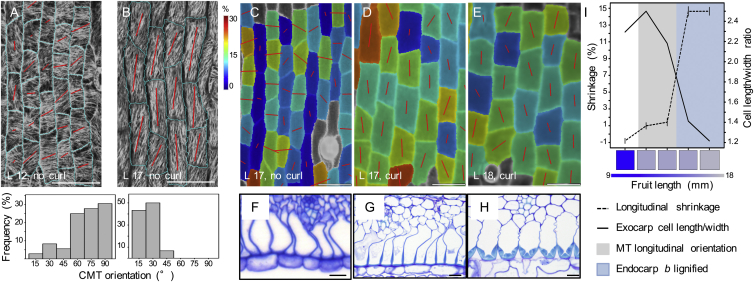
Cellular Determinants of Valve Tension (A and B) Principal direction and degree of cortical microtubule (CMT) alignment (red lines) in exocarp cells (outlined in cyan) of fruit at stage 16 (A, 12 mm fruit length) and stage 17a (B, 17 mm fruit length); CMTs visualized by GFP-TUA6 expression; barplots quantify the distribution of CMT orientations, relative to the longitudinal fruit axis, n = 66 cells. Scale bars, 50 μm. (C–E) Principal direction and amount of tension (red lines) in hydrated exocarp cells during successive stages of fruit development: early stage 17a (C), late stage 17a (D), and stage 17b (E). Heatmap shows tension as % cell shrinkage in the exocarp after tension is release by excising the valve from the fruit. In (A)–(E) images are aligned to the longitudinal fruit axis. L, fruit length in mm; curl, no curl, valve does or does not curl when cut. Scale bars, 50 μm. (F–H) Transverse TBO-stained sections of fruit valves at early stage 17a (F), late stage 17a (G), and stage 17b (H), showing progressive thickening and lignification of endocarp *b* secondary cell walls. Scale bars, 10 μm. (I) Barplot of valve tension (% shrinkage in longitudinal direction, dashed line) shown on left y axis and exocarp cell shape (cell length/width ratio, solid line) shown on right y axis, relative to CMT reorientation (gray) and endocarp *b* lignification (blue) during development, fruit length shown as a heatmap on x axis. See also Figures S5 and S6.

**Figure S5:**
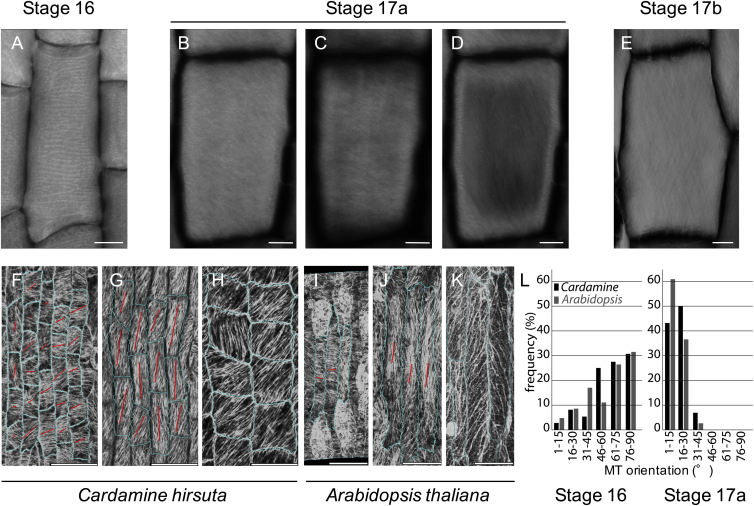
Realignment of Cellulose Microfibrils in the Fruit Exocarp, Related to Figure 6 (A–E) Surface projections of cellulose microfibrils stained with S4B in *C. hirsuta* exocarp cells at successive stages of fruit development. Microfibrils are aligned in a uniformly transverse direction at stage 16 (A) but longitudinally aligned in the inner cell wall at stage 17a (D) such that net alignment is no longer transverse at stage 17b (E). Signal is projected from the following surface depths: 0 - 0.9 μm (total cell wall, A), 0 – 1.4 μm (total cell wall, B), 0 – 0.5 μm (outer cell wall, C), 1 – 1.4 μm (inner cell wall, D) and 0 - 1.6 μm (total cell wall, E). (F–K) Surface projections of *35S::GFP:TUA6* expression marking cortical microtubules (MT) in exocarp cells at successive stages of *C. hirsuta* (F-H) and *A. thaliana* (I-K) fruit development. Please note that panels (F, G) are presented in Figure 6 of the main text but are also shown here for clarity. MT orientation switches from transverse in stage 16 (F, I) to longitudinal in stage 17a (G, J). Cells are outlined in blue, and red lines indicate the principal direction of MT orientation in each cell. At stage 17b (H, K), *C. hirsuta* exocarp cells change shape from rectangular to square (H), while *A. thaliana* exocarp cells remain rectangular (K). This suggests that the MT reorientation alone is not sufficient to change cell shape and that other factors must also contribute to lateral cell expansion in *C. hirsuta*. A possible candidate is the lateral tension exerted by expanding seeds during *C. hirsuta* fruit maturation (Figures S2 and S6). (L) Distribution of MT orientations in stage 16 and 17a exocarp cells of *C. hirsuta* (black) and *A. thaliana* (gray). All images are oriented such that vertical corresponds to the long axis of the fruit. Scale bars: 10 μm (A-E), 50 μm (F-K).

A key prediction from the cellular model is that exocarp cell shortening can only occur in anisotropic cells. To test this, we estimated the degree of cell anisotropy that best fit the measurements from CFM and osmotic treatments in finite element simulations. Our results showed that exocarp cell walls became distinctly anisotropic during development, becoming stiffer in length (along the fruit axis) and softer in width directions (Figure 5H). Because plant cell walls are directionally reinforced by stiff cellulose microfibrils, these results predict a significant change in their net alignment in the cell walls of the exocarp layer during fruit development. Cortical microtubules (CMTs) act as tracks at the plasma membrane to guide cellulose deposition (Paredez et al., 2006). In growing cells, the alignment of CMT arrays predicts the direction of minimal cell expansion, since stiff cellulose microfibrils restrict growth. We observed well-aligned CMT arrays in the exocarp that reoriented from transverse (45°–90°) to longitudinal (0°–30°) before valves were competent to curl (Figures 6A and 6B). We verified a similar reorientation in cellulose microfibril alignment (Figure S5). This reorientation increases longitudinal cell wall stiffness and switches the maximal direction of cell expansion to the width direction, contributing to the change in cell shape from rectangular to square.

**Figure S6:**
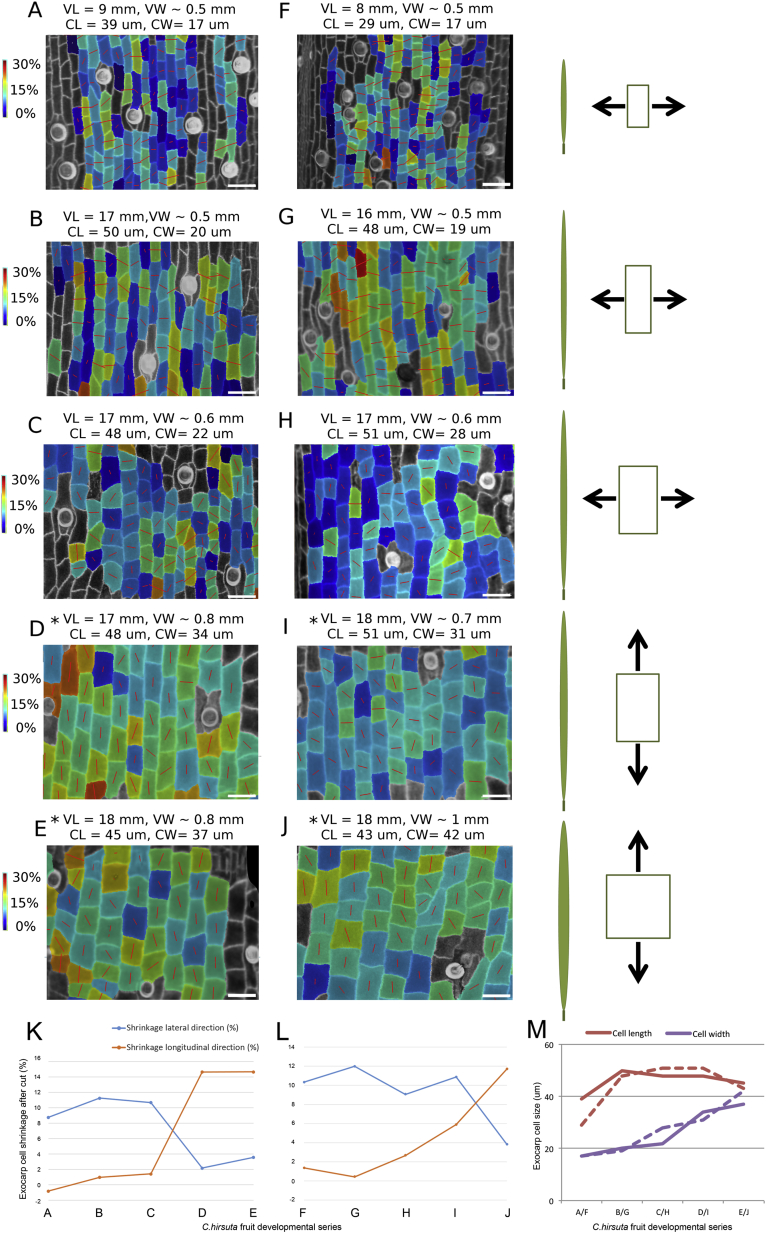
Changes in Exocarp Tension In Situ during *C. hirsuta* Fruit Development, Related to Figure 6 (A–J) Valve regions were imaged on the fruit, then cut off the fruit and re-imaged, and deformations were quantified at the cellular scale. The exocarp shrank after it was cut off the fruit, indicating that it was in a state of tension while attached to the fruit. The direction of maximal shrinkage of the exocarp is quantified in a developmental series of fruits from two different plants (A–E, F–J). All images are oriented such that vertical corresponds to the long axis of the fruit. Red lines show the direction of maximal shrinkage for each cell and the heatmap indicates the percentage of shrinkage in this maximal direction. Fruits at near final length and width (D, E, J) exhibit valve curling after cutting and these exocarp cells are under maximal tension in the longitudinal direction. Prior to this developmental stage, valves do not curl after cutting and exocarp tension is maximal in the lateral direction (A, B, F, G). The switch in direction of maximal tension occurs during an intermediate stage (C, H, I). Please note that panels (B, D, E) are presented in Figure 6 of the main text but are also shown here for clarity. The cartoons to the right of each pair of images summarize the dimensions of the fruit and exocarp cells at each developmental stage, and the principal direction of tension. (K and L) Graphs showing the percentage of exocarp cell shrinkage in lateral (blue) and longitudinal (orange) directions for each fruit developmental series: (A)–(E) shown in (K) and (F)–(J) shown in (L). (M) Graph showing exocarp cell size in the length (red) and width (purple) dimensions for each fruit developmental series (A-E, solid lines) and (F-J, dashed lines). VL: total valve length before cutting, VW: total valve width before cutting, CL: mean cell length, CW: mean cell width. Asterisk indicates valves that curled when detached from the fruit. Scale bar: 50 μm.

To understand whether CMT reorientation and subsequent cell shape change might provide sufficient developmental inputs to cause cell shortening, we analyzed valve tension in situ. Specifically, we analyzed the development of tension in the valve by quantifying the magnitude and principal direction of shrinkage in the exocarp after excision at successive stages of fruit development (Figures 6C–6E, 6I, and S6). In valves that were full-length but not yet competent to curl, maximal tension was aligned across the valve, possibly exerted by expanding seeds (Figures 6C, S3A, S3B, and S6). The tension required to curl is exerted along the length of the valve and increased sharply once exocarp cells began to expand in width, coincident with thickening and lignification of endocarp *b* secondary cell walls (Figures 6D–6I and S6). In summary, our analysis suggests the following sequence of events: a change in cell wall anisotropy following CMT reorientation, coupled with tension in the direction of the fruit width, causes exocarp cell growth to switch from the length to the width direction. This reorientation drives a change in exocarp cell shape from rectangular to square. Subsequent anisotropic deformation of these cells results in their shortening, causing tension to rapidly establish along the length of the valve. This tension is harnessed to drive curvature by coincident stiffening of the endocarp *b* layer (Figure 6I).

### Valve Extension Experiment Links Multi-scale Models

**Figure 7:**
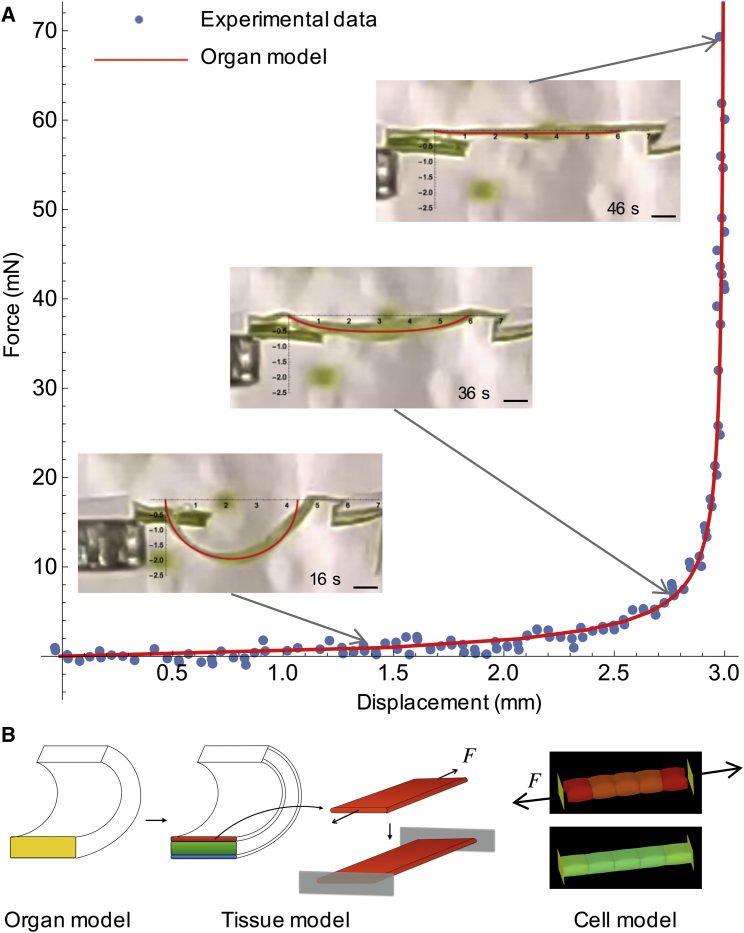
Linking Multi-scale Models (A) Force-displacement curves measured (blue dots) and computed from an organ model (red line) showing the force exerted, as a valve is pulled from curved to flat in air. Insets show valve at three time points during the experiment indicated by arrows on the curve, overlaid in red is the corresponding profile calculated from the organ model. Scale bars, 1 mm. (B) Cartoon of experimental design: valve stiffness (yellow) determined in (A) was used to compute exocarp stiffness (red), from which exocarp pulling force (*F*) was calculated. The same force parameter was extracted independently from the cell-level model. See also Figure S7.

Our results show that the mechanism of explosive seed dispersal has important features at the organ, tissue, and cellular scales. We have developed a series of models to understand each of these features and how they are connected at different scales. To validate our multiscale approach, we devised a link between a mechanical experiment performed at the organ level with a theoretical model built from organ and tissue levels. We then compared the predictions provided independently by this model and our cell-level model (Figure 7; Supplemental Experimental Procedures).

**Figure S7:**
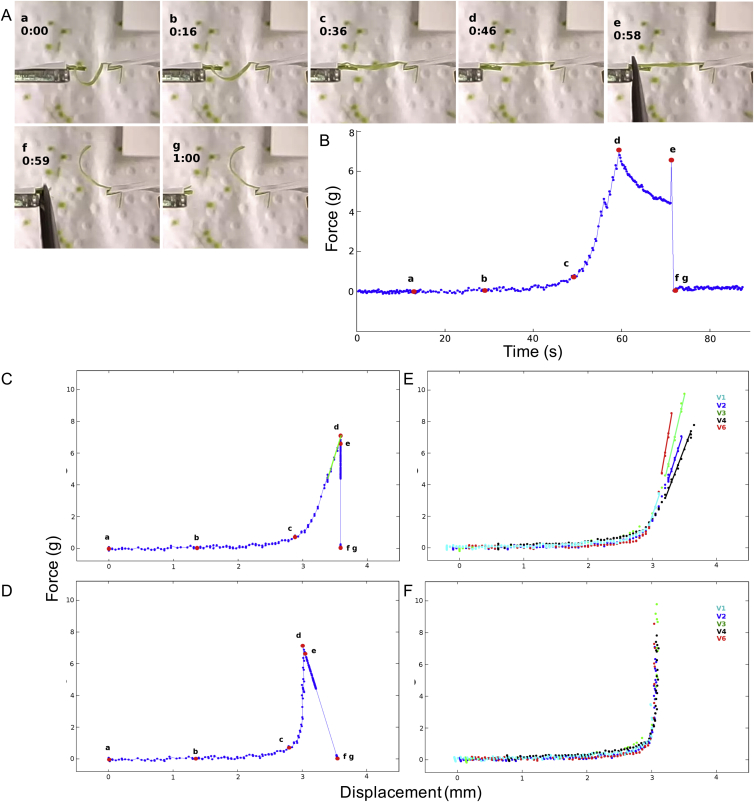
Profile of Extensometer Experiments and Correction for Setup Stiffness, Related to Figure 7 (A) Image sequence (a-g) extracted from a movie of experiment V2 was used to determine the exact time of valve rupture and compare with the force recorded in time. (B) Just before rupture, the force exhibits a local maximum (e) due to the scissors pressing on the valve, after which the force drops to zero and the valve is released from the clamp (f). Using the point of rupture (e) as a reference, the other time points were traced back on the force-time curve: initial pulling on the valve (a) and complete straightening of the valve (d), as well as intermediate points of valve uncurling (b and c). Note that the force decreases with time between (d) and (e) due to creep of the whole experimental setup. (C–F) Force-displacement curves. (C) Curve from experiment V2 shown above; time points (a-g) extracted from the movie are indicated in red. The stiffness of the experimental setup, composed of the force sensor, clamps, actuator and straight valve, is extracted by fitting a line to the force-displacement curve before valve rupture (green line). The setup stiffness is then used to obtain a corrected force-displacement curve (D) that can be fitted to the organ model shown in Figure 7 of the main text. (E) Curves from 5 different experiments (V1, V2, V3, V4, V6), truncated after valve rupture for clarity. Setup stiffness for each experiment was determined based on the linear fit of the curves before rupture (solid lines). Note that clamping of the valve is different for each experiment, resulting in variation in setup stiffness. (F) Each curve corrected for setup stiffness.

To measure the force required to extend whole valves, we clamped a curled valve, freshly excised from the fruit, to a high precision extensometer and recorded force-displacement measurements as the clamps were moved apart, flattening the valve (Figures 7A and S7). We recorded five replicates of this experiment. We then used an organ-level model, treating the excised valve as a single elastic beam with intrinsic curvature, to fit the experimental force-displacement curves and extract the bending stiffness of the whole valve (Figure 7A). In our tissue-level model, we used this effective stiffness to compute the Young’s modulus of the exocarp layer (Figure 7B; Supplemental Experimental Procedures). This is an important parameter of our tissue-level model in determining the total energy available for coiling. Therefore, we used the close match that we obtained between the simulated and measured dynamics of valve coiling and seed launch (Figures 1E and 1K) to validate this parameter value. Hence, an additional outcome of this experiment to bridge different modeling scales was an experimentally determined value for the elasticity of the exocarp.

Next, we compared the value for the Young’s modulus of the exocarp layer, derived above, with an independent calculation from our cell-level model. The Young’s modulus was computed in the cell-level model by simulating a stretched file of turgid exocarp cells and measuring the force exerted at the ends of the file (Figure 7B). Parameters in the cell-level model are based on imaging experiments that were performed in water, where the cells are maximally turgid, therefore the force estimated from this model is expected to be slightly higher than that obtained from valve extension experiments, which were performed in air. The computed force acting on the exocarp layer before explosion in the tissue-level model (mean of five experimental replicates = 37 ± 20 mN, max = 75 mN) is in good agreement with the value obtained from the cellular model (61 ± 9 mN), showing that the predictive value of each model is consistent across scales. Note that this force corresponds to an approximate weight of 5 g; four orders of magnitude greater than a *C. hirsuta* seed, illustrating the considerable force required to disperse small projectiles that are dominated by drag (Vogel, 2005).

### Conclusions

Through a combined experimental and theoretical analysis, we have identified cellular innovations for the storage and rapid release of energy that underpin the evolution of explosive seed dispersal in *C. hirsuta*. Our study demonstrates the strength of combining a genetically tractable system with theoretical models at different spatial scales to obtain an integrated and comprehensive understanding of the developmental and mechanical basis of this rapid plant movement. In particular, we have shown that the mechanical catalyst for explosive energy release is the hinged cell wall of a single fruit layer, which appears to have evolved once in the Brassicaceae and promoted ballistic seed dispersal. A specific prediction from our work is that genes regulating secondary cell wall synthesis and patterning are likely targets of evolutionary modification. Additionally, we found that tissue shortening in the fruit valve does not arise from passive shrinkage as previously thought, but is an active, turgor-driven process dependent on the three-dimensional geometry and anisotropy of exocarp cells. It will be interesting to determine whether this cellular mechanism, that shares design principles with robotic “air muscles,” has been used by other organisms as a common evolutionary solution to power rapid movements.

## Experimental Procedures

### Plant Material and Generation of Transgenic Plants

The *C. hirsuta* reference accession Oxford (Hay and Tsiantis, 2006) was used as wild-type in this study. The *lig2* mutant was isolated from an EMS screen and the causal mutation identified by map-based cloning and whole genome sequencing. The following transgenes were constructed using multisite Gateway in the pGREENII125 binary vector containing norflurazon selection: p*LIG2::*g*LIG2-vYFP*, p*LIG2::*g*lig2-vYFP*, *Ch*p*NST3::AtVND7-vYFP* and *Ch*p*NST3::GUS*. *35S::GFP:TUA6* was described previously (Ueda et al., 1999). Constructs were transformed into *C. hirsuta* by floral dip using *Agrobacterium tumefaciens*. Primers used for plasmid construction and gene expression analysis are listed in Table S2.

### Image Analysis, Microscopy, and Osmotic Treatments

MorphoGraphX was used for quantitative image analysis (Barbier de Reuille et al., 2015). The main axes of cellular deformation were quantified using the algorithm normally used to compute principal directions of growth. The principal orientation of GFP-TUA6 signal was computed using the Fibril orientations algorithm. Images were acquired by confocal laser scanning microscopy, light microscopy, and transmission electron microscopy. Osmotic treatments were performed with segments of fruit valve pre-stained with propidium iodide (PI). Turgid cells were imaged in deionized water. These samples were then treated with 1 M NaCl, re-stained, and plasmolyzed cells were reimaged in deionized water. GUS, lignin, and cellulose microfibril staining were performed as previously described (Landrein et al., 2013, Liljegren et al., 2000, Roeder et al., 2003).

### Force Measurements and High-Speed Videography

Cellular force microscopy was performed as previously described (Routier-Kierzkowska et al., 2012, Weber et al., 2015). A high precision extensometer was custom built from a miniature load cell and a piezoelectric micropositioner arm, which progressively stretched the whole fruit valve by increments of 50 μm. Explosive pod shatter was filmed with two synchronized high-speed cameras fitted either with 55 mm or 105 mm lenses and configured to save images at 1,500 frames per second (fps) and 256 × 1,024 pixels, or at 15,000 fps and 256 × 272 pixels. Seed trajectories were tracked manually using software custom-written in MATLAB (Walker et al., 2009).

Further details of these methods and a detailed description of all models can be found in the Supplemental Experimental Procedures.

## Author Contributions

H.H., A.R-K., R.J.B., S.W., H.R., P.S., X.G., M.T., and A.H. produced experimental data. D.M., T.L., A.G., Y.V., G.M., and R.S. produced modeling data. R.J.B. and S.W. designed high-speed imaging experiments and R.J.B. analyzed this data. A.H. wrote the paper and designed the study together with D.M., T.L., A.G., and R.S. who designed modeling approaches. D.M. and R.S. wrote the modeling parts of the extended experimental procedures. H.H., D.M., T.L., A.R-K., and R.J.B. contributed equally to the study. All authors discussed results and commented on the manuscript.

## Accession Numbers

The accession number for the genomic DNA sequence of *C. hirsuta LIG2* gene (CARHR188820) reported in this paper is GenBank: KX063714.

## Supplemental Information

Document S1. Supplemental Experimental Procedures and Tables S1 and S2

Movie S1. *C. hirsuta* Explosive Pod Shatter, Related to Figure 1 Recorded at 15,000 fps.

Movie S2. Coiling Valve Dynamics Using Lagrangian Characterization, Related to Figure 1 Axes show distance in mm.

Movie S3. Turgor-Driven Shrinkage, Related to Figure 5 Simulations from a finite element model of exocarp cells pressurized from 0 to 0.7 MPa; colored according to a heatmap scale of relative increase (orange) or decrease (blue) in cell length, horizontal yellow line shows initial length. Cells of dimensions 100 × 20 × 20 μm with isotropic wall material (final frame shown in Figure 5D).

Movie S4. Turgor-Driven Shrinkage, Related to Figure 5 Simulations from a finite element model of exocarp cells pressurized from 0 to 0.7 MPa; colored according to a heatmap scale of relative increase (orange) or decrease (blue) in cell length, horizontal yellow line shows initial length. Cells of dimensions 50 × 50 × 20 μm with isotropic wall material (final frame shown in Figure 5E).

Movie S5. Turgor-Driven Shrinkage, Related to Figure 5 Simulations from a finite element model of exocarp cells pressurized from 0 to 0.7 MPa; colored according to a heatmap scale of relative increase (orange) or decrease (blue) in cell length, horizontal yellow line shows initial length. Cells of dimensions 50 × 50 × 20 μm with anisotropic wall material (final frame shown in Figure 5F).

Document S2. Article plus Supplemental Information
