# Supplementary figures and images for: Morphomechanical Innovation Drives Explosive Seed Dispersal
Source: Cell. 2016 Jun 30;166(1):222–33. doi: 10.1016/j.cell.2016.05.002 (PMC4930488; doi:10.1016/j.cell.2016.05.002)

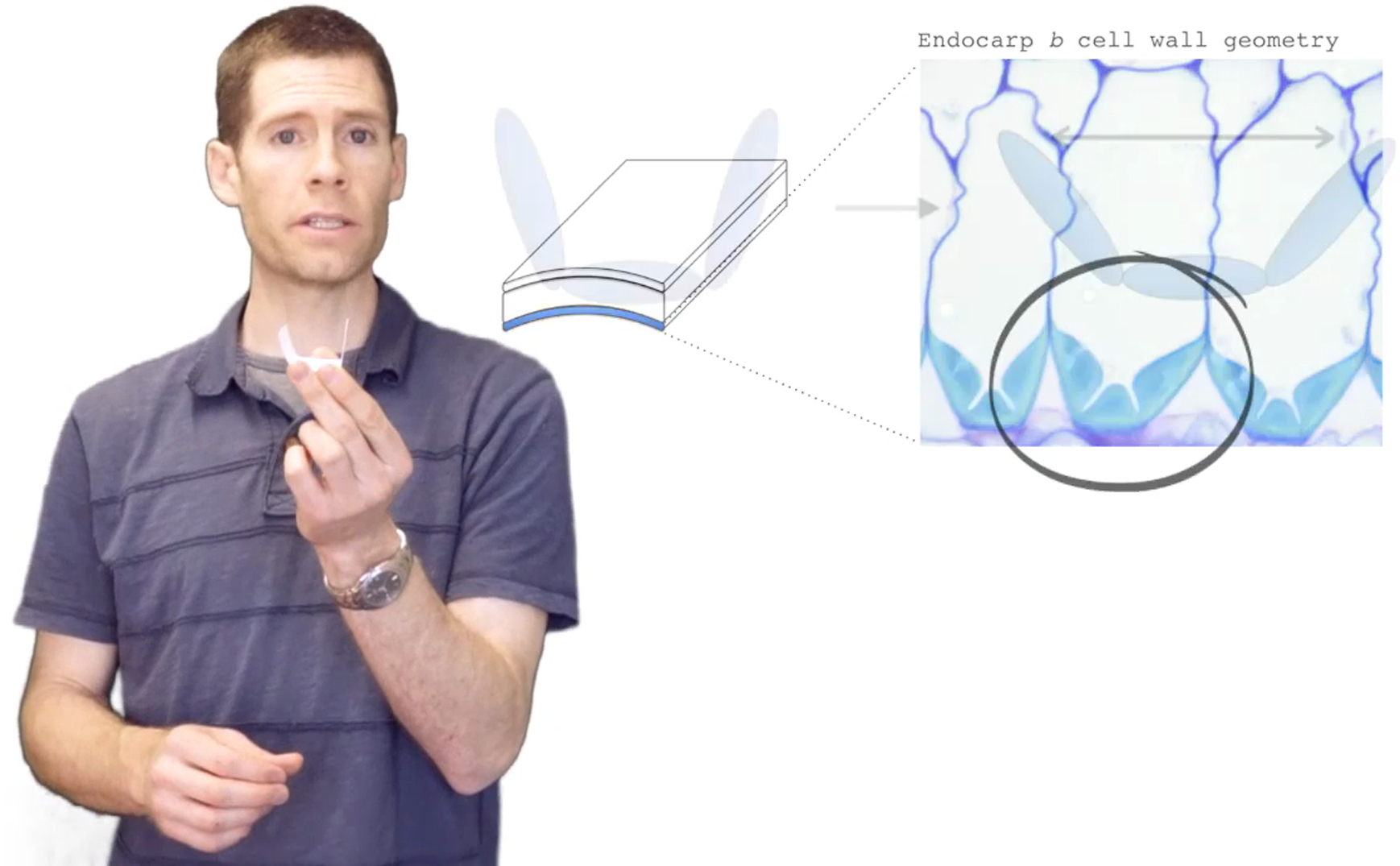

Supplement: Supplementary file 1 [file mmc8.jpg]

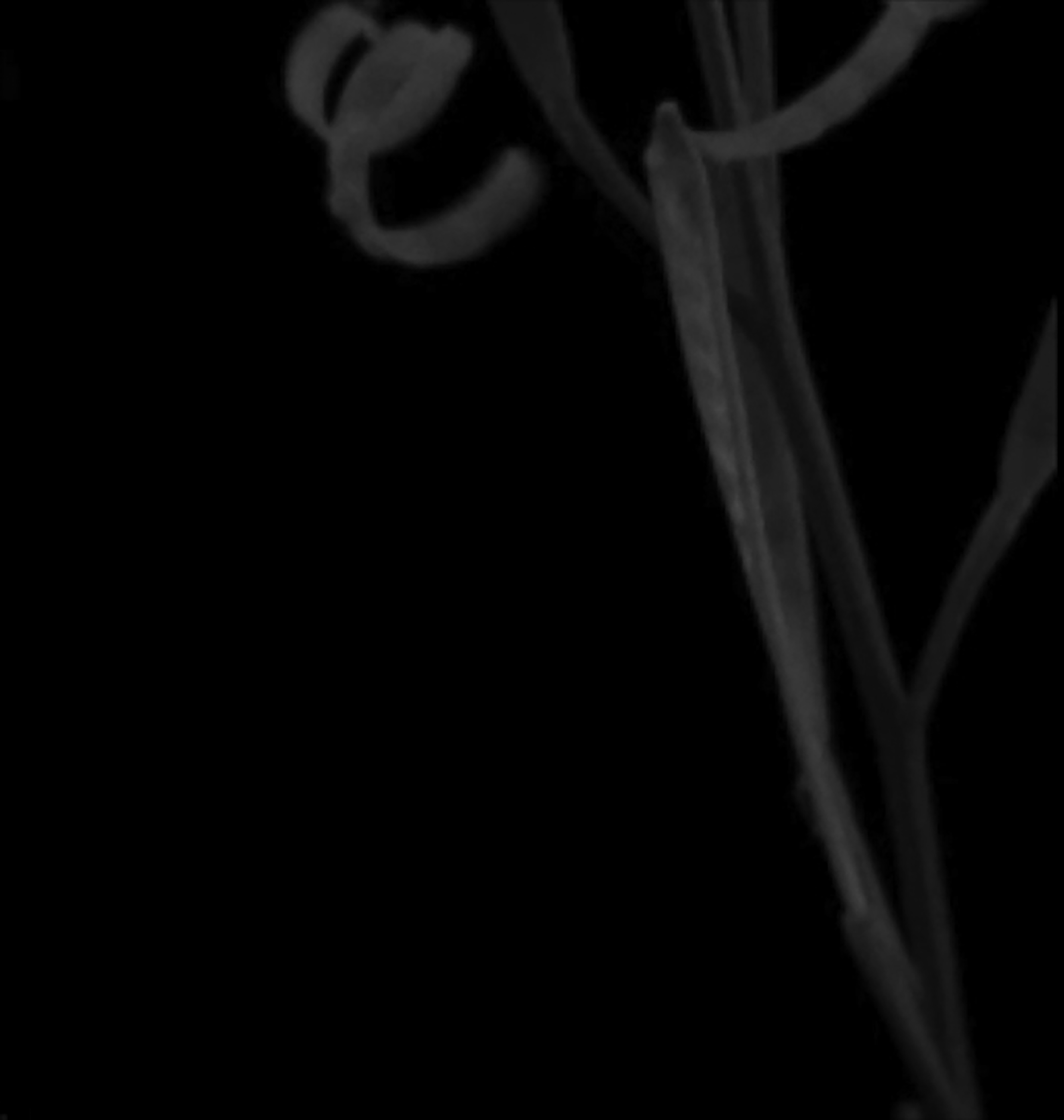

Supplement: Movie S1. C. hirsuta Explosive Pod Shatter, Related to Figure 1 — Recorded at 15,000 fps. [file mmc2.jpg]

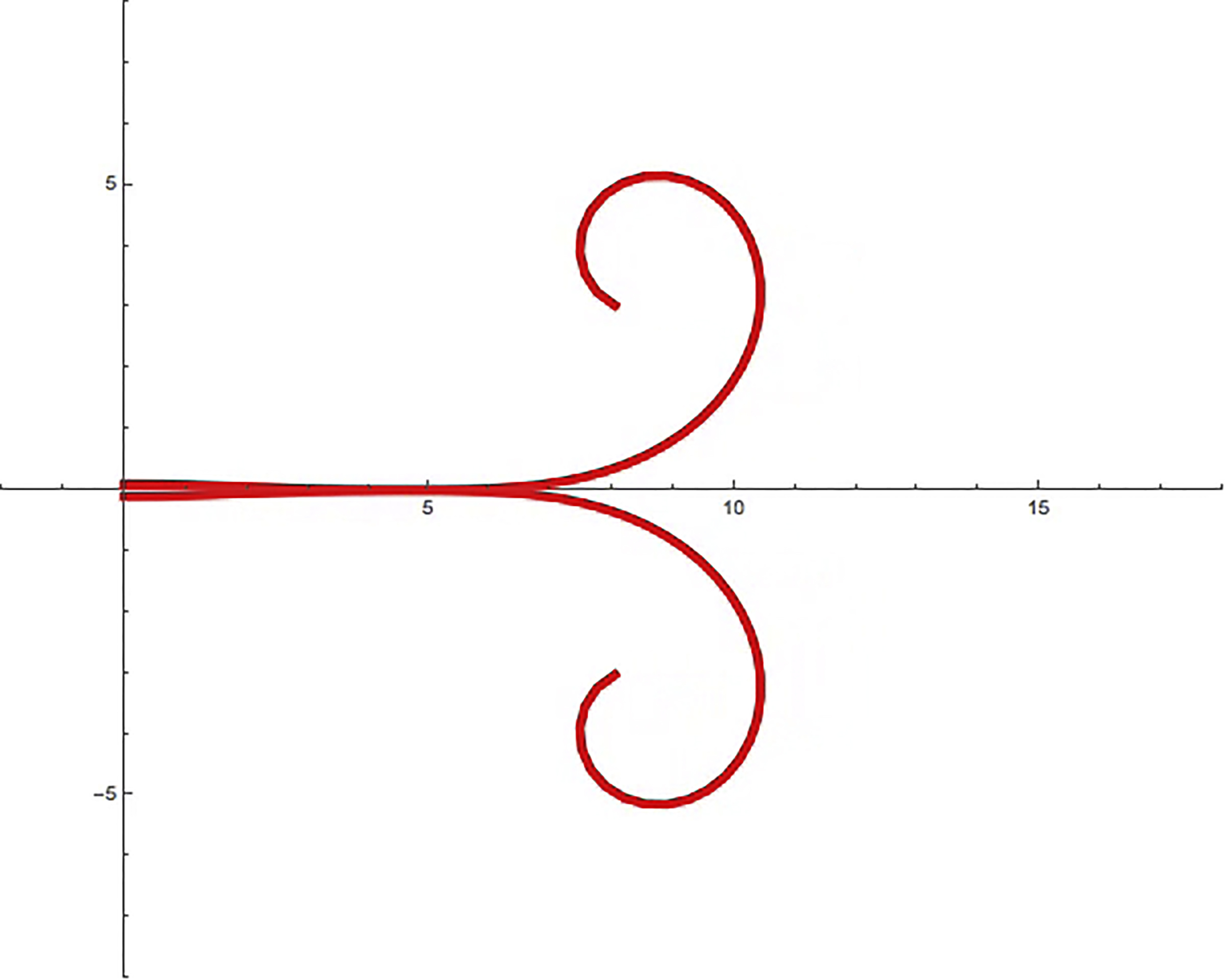

Supplement: Movie S2. Coiling Valve Dynamics Using Lagrangian Characterization, Related to Figure 1 — Axes show distance in mm. [file mmc3.jpg]

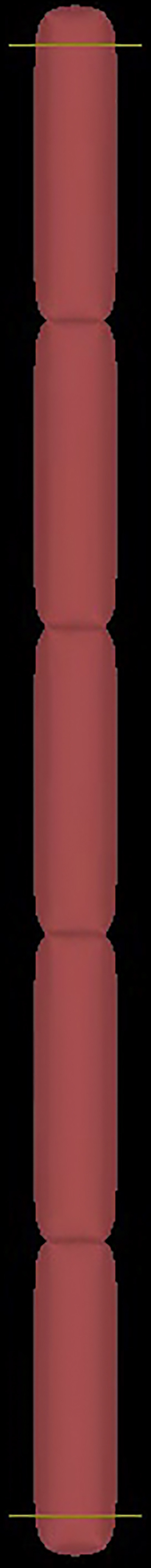

Supplement: Movie S3. Turgor-Driven Shrinkage, Related to Figure 5 — Simulations from a finite element model of exocarp cells pressurized from 0 to 0.7 MPa; colored according to a heatmap scale of relative increase (orange) or decrease (blue) in cell length, horizontal yellow line shows initial length. Cells of dimensions 100 × 20 × 20 μm with isotropic wall material (final frame shown in Figure 5D). [file mmc4.jpg]

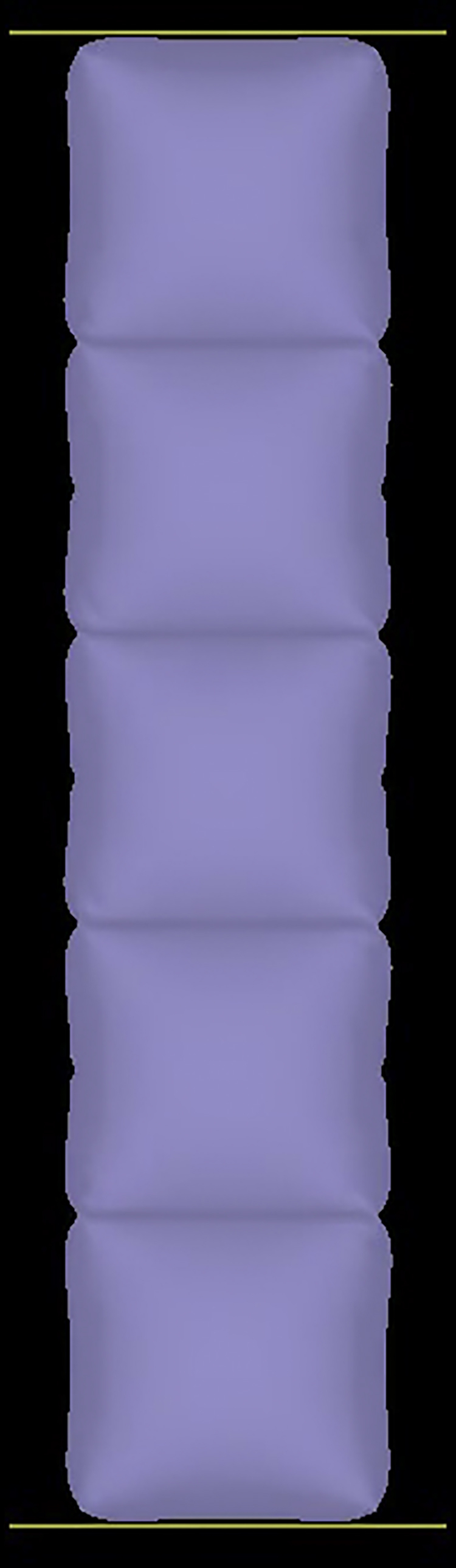

Supplement: Movie S4. Turgor-Driven Shrinkage, Related to Figure 5 — Simulations from a finite element model of exocarp cells pressurized from 0 to 0.7 MPa; colored according to a heatmap scale of relative increase (orange) or decrease (blue) in cell length, horizontal yellow line shows initial length. Cells of dimensions 50 × 50 × 20 μm with isotropic wall material (final frame shown in Figure 5E). [file mmc5.jpg]

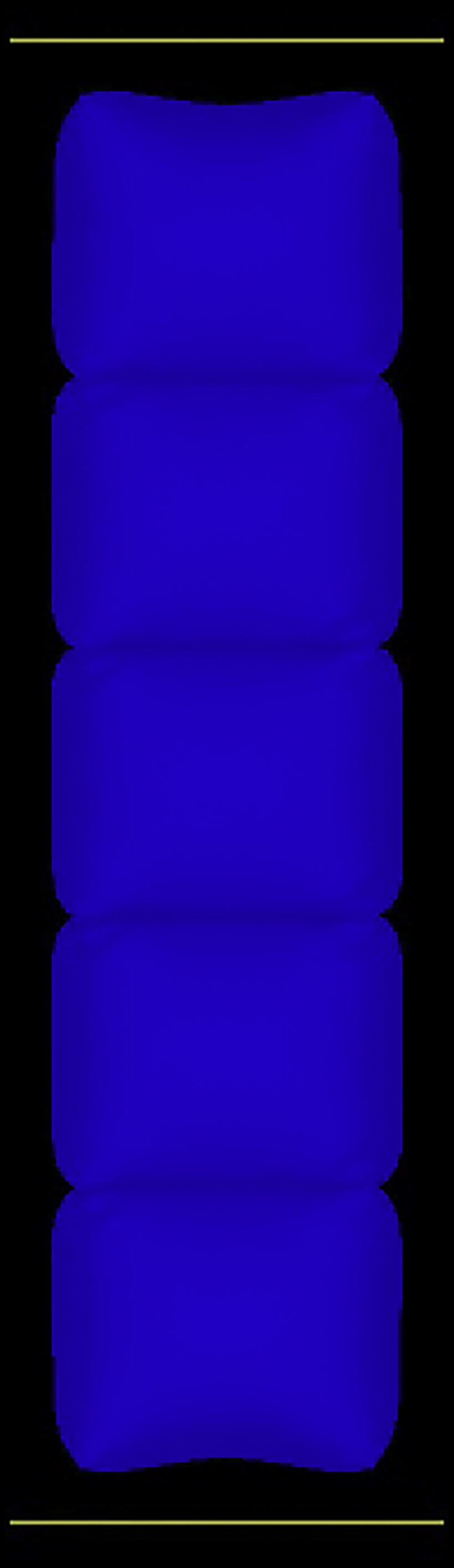

Supplement: Movie S5. Turgor-Driven Shrinkage, Related to Figure 5 — Simulations from a finite element model of exocarp cells pressurized from 0 to 0.7 MPa; colored according to a heatmap scale of relative increase (orange) or decrease (blue) in cell length, horizontal yellow line shows initial length. Cells of dimensions 50 × 50 × 20 μm with anisotropic wall material (final frame shown in Figure 5F). [file mmc6.jpg]
