# Supplementary material for: Morphomechanical Innovation Drives Explosive Seed Dispersal
Source: Cell. 2016 Jun 30;166(1):222–33. doi: 10.1016/j.cell.2016.05.002 (PMC4930488; doi:10.1016/j.cell.2016.05.002)
Supplement: Document S1. Supplemental Experimental Procedures and Tables S1 and S2 [file mmc1.pdf]

Cell, Volume *166*

Supplemental Information

Morphomechanical Innovation

Drives Explosive Seed Dispersal

Hugo Hofhuis, Derek Moulton, Thomas Lessinnes, Anne-Lise Routier-Kierzkowska, Richard J. Bomphrey, Gabriella Mosca, Hagen Reinhardt, Penny Sarchet, Xiangchao Gan, Miltos Tsiantis, Yiannis Ventikos, Simon Walker, Alain Goriely, Richard Smith, and Angela Hay

Extended Experimental Procedures

Plant material

Plants were grown on soil in a greenhouse under long day conditions [16 h light (20°C) and 8 h dark (16°C)] with supplemental lighting. Seeds were stratified for 2-4 days at 4°C before sowing. In all cases, the *C. hirsuta* wild type is the reference Oxford (Ox) accession [specimen voucher Hay 1 (OXF)] (Hay and Tsiantis, 2006). The *lig2* mutant was isolated from an EMS mutagenesis screen and is monogenic recessive. Other Brassicaceae species analyzed: *C. flexuosa, C. parviflora, C. alpina, C. resedifolia, C. corymbosa, C. trichocarpa, Leavenworthia alabamica, Rorippa islandica, Arabidopsis thaliana, Olimarabidopsis pumila, Brassica rapa, Turritus glabra, Turritus laxa, Pseudoturritis turrita, Bochera stricta, Braya alpina, Arabis sagittata, Physaria fendleri, Camelina microcarpa, Capsella grandiflora, Lepidium apetalum, Geoccocus pusilus, Heliophila coronopifolia*.

Ethyl methane sulfonate (EMS) mutagenesis, mutant screen and mapping

C. hirsuta seeds were washed for 15 mins in 0.1% Triton® X-100 and mutagenized by agitation with 0.18% EMS (Sigma) in deionized water for 10 h, washed extensively in deionized water, with the final wash replaced with 0.1% agarose and seeds then sown on soil and selfed progeny harvested individually from 515 M1 plants (Vlad et al., 2014). Fruits of 13,905 M2 plants were subsequently stained for lignin and screened for pod shatter. Stage 17b siliques, which in the wild type have undergone lignification of the endocarp *b* and the dehiscence zone, were pooled from multiple individuals of a single M2 family and stained with phloroglucinol as described below. When an aberrant phloroglucinol-staining pattern was observed within a pool, corresponding plants were stained individually to identify the mutant plant, which was then backcrossed twice to wild type before analysis. The *lig2* mutant was isolated from M2 A279-i. 277 homozygous *lig2* mutants were analyzed from a F2 mapping population generated by selfing the progeny of a cross between the *lig2* mutant and the polymorphic Wa accession of *C. hirsuta*. Rough mapping was performed on pooled samples containing equal amounts of DNA from 36 individuals. Seven markers, located on chromosome 6 between positions 14978109 bp and 15807704 bp, were subsequently analysed in each of the 277 individual *lig2* DNA samples. Eight additional markers were then analysed in the remaining *lig2* recombinants to fine-map the *lig2* mutation between positions 15429314 bp and 15539723 bp. Primer sequences for markers are given in Table S2. The EMS-induced SNPs in this interval were identified by Illumina Whole Genome Sequencing. DNA was extracted from pooled tissue of 48 *lig2* individuals from the F2 mapping population and sequenced on the Illumina HiSeq 2000 platform (MPIPZ genome centre). SNP sites were identified by comparison with the Ox reference genome sequence. We excluded SNPs derived from the Wa accession by comparison to genome resequencing data which left three EMS-derived SNPs in the interval, including one non-synonymous SNP. The software package IMR/DENOM was used for SNP calling with a cutoff of Phred score at 20 (Gan et al., 2011).

Seed distribution

Twenty one *C. hirsuta* plants were grown in a large greenhouse without supplemental lighting or temperature regulation at Wytham Field Station, University of Oxford, in the summer of 2009. Each plant was grown in a single pot, fitted with an Aracon base (Arasystem) of 5 cm diameter. Just before the plants set seed, they were staked upright and placed at the centre of a series of concentric rings drawn on a large sheet of plastic. Circles were drawn every 25 cm until 175 cm, with an additional circle at 15 cm, such that a total diameter of 3.5 m was assayed around every plant. Following seed dispersal, every seed in each of the concentric rings and the Aracon was counted for each plant and an average for each ring calculated; 52,585 seeds were counted in total. Fruits that matured on *C. hirsuta* plants after cessation of watering did not explode and the seed in these fruits were not included in our analysis.

Binary plasmid construction and generation of transgenic plants

A 3.4 kb fragment starting at position -1 of the upstream regulatory sequence of the *C. hirsuta NST3* gene was amplified with the primer combination pNST3-F and pNST3-R. A 0.5 kb fragment of the upstream regulatory sequence of *LIG2* was amplified using the primer combination pLIG2-F and pLIG2-R. Both promoter sequences were subsequently recombined into a pGEMt-easy1R4 entry vector (Galinha et al., 2007) using BP clonase II Plus (Life Technologies). Full length *LIG2* (2.8 kb) and truncated *lig2* (2.5 kb) genomic sequences were amplified using the primer combinations gLIG2-F1 & gLIG2-R1 and gLIG2-F1 & glig2-R1, respectively. Both fragments were recombined in a pGEMt-easy221 entry vector. All sequences were amplified from *C. hirsuta* genomic DNA; primer sequences are listed in Table S2. We used

multisite Gateway (MSG) LRII plus recombination to generate the following expression constructs. A *Ch*p*NST3::AtVND7-vYFP* expression construct was generated by recombining *Ch*p*NST3* pGEMt-easy1R4, the *A. thaliana VND7* pGEMt-easy221 (Bennett et al., 2010) and Venus YFP-nosT pGEMt-easy2R3 (Prasad et al., 2011) entry vectors. A *Ch*p*NST3::GUS* transcriptional reporter was generated by recombining *Ch*p*NST3* pGEMt-easy1R4, *GUS* pGEMt-easy221 and nosT pGEMt-easy2R3 entry vectors. A *LIG2::gLIG2-vYFP* translational fusion was generated by recombining p*LIG2* pGEMt-easy1R4, *gLIG2* pGEMt-easy221 and Venus YFP-nosT pGEMt-easy2R3 entry vectors. A *LIG2::glig2-vYFP* translational fusion was generated by recombining p*LIG2* pGEMt-easy1R4, *glig2* pGEMt-easy221 and Venus YFP-nosT pGEMt-easy2R3 entry vectors. All entry vectors were recombined into a MSG-compatible pGREENII destination vector (Galinha et al., 2007). The pGREENII125 binary vector used contained norflurazon selection. A *35S::GFP:TUA6* construct in a kanamycin selectable binary vector was previously described (Ueda et al., 1999). All constructs were transformed into *C. hirsuta* by floral dip using *Agrobacterium tumefaciens* strain GV3101 or C58 (Hay et al., 2014). For norflurazon selection, T1 seeds were sown on wet paper, sprayed with 1 μM norflurazon (in dH_2O, Supelco, N12668), stratified at 4°C, germinated under greenhouse conditions in the dark and subsequently exposed to light. For kanamycin selection, T1 seeds were selected on MS plates supplemented with 100 μg/ml kanamycin (in dH_2O, Sigma). Transgenic T1 plants were transplanted to soil and self-pollinated. For each construct a minimum of 10 independent T1 lines were obtained, of which at least 3 independent, single insertion T2 lines were analyzed in detail.

RT-PCR analysis

Transgene expression was analysed in stage 16 fruits of the following genotypes: *lig2, lig2* expressing the complementing p*LIG2::gLIG2-vYFP* transgene or the non-complementing p*LIG2::glig2-vYFP* transgene. Fruit tissue was harvested and immediately frozen in liquid nitrogen. Total RNA was extracted from 5 fruits per sample using the Spectrum Plant Total RNA Kit (Sigma, STRN50) and treated on-column with DNaseI (Sigma, DNASE70). One μg of eluted RNA was used for complementary DNA (cDNA) synthesis using the SuperScriptIII kit (Invitrogen). A fragment was amplified from the reference gene *C. hirsuta ACT8* (CARHR094190) to validate that each sample contained equal amounts of cDNA using the primer combination ACT8-F & ACT8-R. Transgene expression was examined by RT-PCR using the primer combination LIG2-F2 & YFP-R, which amplified a 402 bp product from the complementing *LIG2-YFP,* and a 224 bp product from the *lig2-YFP* transgene. Primer sequences are listed in Table S2.

Quantitative RT-PCR analysis

Gene expression levels were quantified in tissue samples from intact *C. hirsuta* wild-type fruits at developmental stages 9, 15, 16 and 17, and stage 17 fruits dissected into valves, seeds, and remaining fruit tissues. All samples were immediately frozen in liquid nitrogen and stored at -80°C before processing. Total RNA was extracted from 100 mg of tissue using the Spectrum Plant Total RNA Kit (Sigma, STRN50), treated on-column with DNaseI (Sigma, DNASE70), and 1 μg of eluted RNA was used for complementary DNA (cDNA) synthesis using the SuperScriptIII kit (Invitrogen). cDNA templates were used in qPCR reactions to amplify *LIG2*, with the primer pair qLIG2-F3 & qLIG2-R3, and *AP2M*, with the primer pair AP2M-F & AP2M-R, using Power SYBR Green Supermix (Applied Biosystems) on a ViiA7 machine (Applied Biosystems). Transcript levels for each gene were calculated using the formula (Etarget)–ΔCPtarget (control-sample)/(Eref)–ΔCPref (control-sample) (Pfaffl, 2001) and expression of *LIG2* was normalized to the reference gene *Clathrin*/*AP2M* (CARHR174880). Primer sequences are listed in Table S2.

High-speed filming and image analysis

Explosive pod shatter was filmed with two synchronized high-speed cameras (Photron Fastcam SA3 120K-M2, Photron Europe Ltd, Bucks, UK) and different experimental set-ups were used in order to analyse the trajectories of the valves and the seeds. In both cases, c. 30 stereo images were taken of a custom calibration grid held in a range of orientations and positions within the volume of view. These calibration images were used to calibrate the space according to a photogrammetric model described in (Walker et al., 2009) using custom Matlab code (The Mathworks Inc., Natick, MA).

To film the initial stages of pod shatter, cameras were fitted with 105 mm lenses (Sigma 1:2.8DG Macro) and configured to save images to a continuous circular buffer at 15000 frames per second at a resolution of 256 × 272 pixels. Fruits were illuminated with three 96-LED panels (HDV-Z96) and cameras were triggered automatically when an exploding valve tripped a light beam linking a laser pointer to a photodiode in a custom circuit. We centre-triggered the cameras and saved images either side of the trigger. Image sequences were saved using Photron FASTCAM Viewer software (Ver. 3273). Plants were

monitored for no longer than overnight and discarded if a pod shatter event did not occur in this time. Three recordings were selected for full kinematic analysis of valve dynamics as described below in the modeling section.

To capture seed trajectories, the cameras were fitted with 55 mm lenses and configured to save images at 1500 frames/second and a resolution of 256 × 1024 pixels. Mature fruits were carefully excised from the plant and the pedicel clamped to position the fruit upright. Fruits were illuminated from above and from the side. Applying gentle vibration to the fruit with the metal projection from an electric toothbrush induced pod shatter and cameras were post-triggered manually. Twenty fruit recordings were selected for tracking and analysis of seed trajectories. The image coordinates for each seed were digitised manually across the frames for which they were visible to both cameras using tracking software custom-written in Matlab (Walker et al., 2009). We measured seed launch conditions for 229 seeds from 14 fruits and predicted seed trajectories using iterative ballistics incorporating drag.

Seed velocities were measured from movies recorded at 1500 fps. We could not measure the muzzle velocity of seeds because the coiling valve often obscured our view of their initial release. In addition, the location of the first sighting of each seed (and hence the distance already travelled along its total trajectory) was variable between seeds. Therefore, our measured dynamics may slightly underestimate the real seed dynamics because seeds may have already slowed a little before we could observe their flight. For these reasons we show both maximum and mean values.

<u>Pontamine Fast Scarlet 4B staining (adapted from (Landrein et al., 2013))</u>
Pontamine Fast Scarlet 4B (S4B) stain was used to visualize cellulose microfibrils in valve exocarp cells. Siliques from a series of developmental stages were harvested, fixed in 6:1 (v/v) acetic acid/ethanol for 10 minutes, cut into 3-5 mm segments and fixed for a further 20 minutes while shaking. Tissue was washed twice with 100% ethanol and once with 50% ethanol, for 30 minutes each wash. Valves were then excised from the fruit segments by cutting along the valve margin and stored in phosphate buffered saline (PBS pH 7.3) for at least 30 minutes. Valve segments were then stained for 2-6 hrs with 5 µg/ml Direct Red23 (Sigma Aldrich, catalogue number 212490) in PBS (pH7.3) and rinsed with deionized water before analysis. Stain accumulated in 3-4 cell layers adjacent to a cut surface.

<u>Propidium iodide (PI) staining and osmotic treatments</u>
To visualize exocarp cell walls in intact mature fruits, valves were punctured with a small needle to facilitate uptake of 1% PI in deionized water. Exocarp cells distant from the wound site were then imaged. Valve segments of 2-4 mm length, containing the previously imaged cells, were either detached from the fruit by cutting along the valve margin or the outer valve layers only were detached. Segments were re-stained with PI for an additional 5 minutes before being re-imaged.

Osmotic treatments were performed with 2-4 mm valve segments of mature fruit pre-stained with 1% PI in deionized water for 5~10 minutes. Exocarp cells were imaged in deionized water (turgid), transferred into 1M NaCl for 45~60 minutes, re-stained with 1% PI for 5 minutes, and reimaged in deionized water (plasmolyzed). Plasmolysis was assessed by retraction of the plasma membrane away from the cell wall.

<u>Confocal Laser Scanning Microscopy (CLSM)</u>
A Leica DM6000 TCS SP8 microscope equipped with a HyD detector was used with a 100x/1.3 oil immersion objective to visualize S4B stained cellulose microfibrils (excitation 514 nm, emission 584-630nm). Samples were mounted in water on microscope slides and Z-stacks were acquired with 0.1 µm interval. 20x/0.5 and 63x/0.9 water immersion objectives were used to visualize DAPI (excitation 405, emission 416-502), *LIG2*-YFP and *lig2*-YFP fluorescence (excitation 514, emission 520-542). A Leica TCS SP2 microscope fitted with long distance water immersion objectives was used to visualize GFP-TUA6 (excitation: 488 nm, emission: 495-545 nm), lignin auto-fluorescence (excitation 405nm, emission 450-550 nm) and PI (excitation 488nm, emission 600-650nm) fluorescence. Z-stack step size was optimized for the objective used and ranged between 0.5 µm and 0.25 µm for 40x and 63x objectives, respectively. All samples were submerged in deionized water during imaging. Valve segments and sections sink naturally; whole fruit samples were fixed with adhesive tags to the bottom of small petri dishes.

<u>Light microscopy</u>
A Zeiss AxioImagerD2 compound microscope equipped with an Axiocam HR3 camera and a 100x/1.3 oil immersion objective was used to image semi-thin sections of paraffin and plastic-embedded tissues. A Zeiss Axiophot compound microscope equipped with a Leica DFC490 camera and 20x and 40x objectives was used to image fresh tissues. A Nikon SMZ18 stereoscope equipped with equipped with a DS-Fi camera was

used to image whole valves.

Transmission electron microscopy

Small sections of fruit were isolated under the surface of the fixative (3% glutaraldehyde, 2% paraformaldehyde in 0.03 M phosphate buffer, pH 7.2), degassed using a vacuum pump and then fixed at room temperature for 6 hrs. After washing in buffer, the tissue was postfixed on 1.5% osmium tetroxide in distilled water for 3 hr at room temperature, washed in distilled water, dehydrated in an ethanol series and embedded in medium-hard embedding resin (TAAB Laboratory Equipment Cat T262) following the manufacturer's instructions. Once hardened, gold-refracting sections were cut from the blocks using a LKB Ultracut microtome, post-stained in uranyl acetate and lead citrate, and viewed in a JEOL 2000CX transmission electron microscope operating at 80 kV.

Toluidine blue staining of paraffin and plastic-embedded tissue sections

Mature fruits were cut into 2-3 mm segments and processed in one of three ways. Samples were either fixed in 4% formaldehyde freshly prepared from paraformaldehyde, processed through to paraffin using a Tissue-Tek® processor (Sakura Finetek USA, Inc), and 8 µm sections were stained with 0.05% toluidine blue; or samples were fixed in 2.5% glutaraldehyde in phosphate buffer, dehydrated, step-wise infiltrated with and embedded in TAAB Low Viscosity resin (TAAB), and 1.5 µm sections were stained with 0.05% toluidine blue. Alternatively, samples were fixed in 2% paraformaldehyde, 2% glutaraldehyde in 0.1M sodium cacodylate buffer (pH 6.9) for 2 hours at room temperature then overnight at 4 °C, and post-fixed for 2 hours at room temperature in 1% aqueous osmium tetroxide. After washing and dehydration, these samples were equilibrated gradually in acetone, embedded in Agar Low Viscosity Resin (Plano GmbH) for 8 days, polymerized in flat embedding moulds at 60 °C for 24 hours, and 1 µm thin sections were stained with 1% toluidine blue supplemented with 1% sodium tetraborate, and mounted permanently in Low Viscosity Resin (Agar Scientific).

Fresh tissue sections

Mature fruits were embedded in 10% low melting agarose in deionized water. Agar was kept at 45 °C and rapidly transferred to ice while embedding the sample. Agar blocks were cut into 4-6 mm long stubs and samples were cut into 100-250 µm sections with a TPI Vibratome series 1000 (Technical Products International Inc.). Vibratome sections of 70 µm were made using a Leica VT 1000 S vibratome. Sections were either stained with 1% PI in deionized water or directly imaged in deionized water by CLSM.

Phloroglucinol staining

For whole-mount visualization of lignin, fruits were first fixed in 14.2% acetic acid in ethanol for 1 to 4 hours shaking at room temperature, or overnight shaking at 4 °C, then rinsed and washed with 100% ethanol for 5 minutes and 70% ethanol for 2 minutes before being cleared for 1 to 3 hours in chloral hydrate solution. Fixed and cleared fruits were stained with phloroglucinol solution (1.72% phloroglucinol, 81.9% ethanol, 13.8% hydrochloric acid in water) and inspected after 10, 20 and 40 minutes (adapted from (Liljegren et al., 2000)). To visualize lignin at the tissue level, fruits were freshly cut into 70 µm sections with a vibratome, as described above, and sections were transferred to chambers demarcated by tough tags on glass slides and stained with phloroglucinol solution as above. To visualize lignin at the cellular level, samples were fixed, paraffin embedded and sectioned as described above. After paraffin removal, sections were stained with 2% phloroglucinol w/v in 95% ethanol for 2-5 min, washed in 10 N hydrochloric acid for 1 min, rinsed in deionized water, and mounted in 5 N hydrochloric acid for viewing (adapted from (Mitsuda and Ohme-Takagi, 2008)).

GUS staining (adapted from (Roeder et al., 2003))

Fruits were prefixed in cold (4 °C) 90% acetone at room temperature for 20 minutes and rinsed with deionized water. Cold GUS staining solution (50 mM phosphate buffer pH 7.2, 0.2% triton X-100, 5 mM Ferro- and Ferri-cyanide and 2 mM X-Gluc (5-Bromo-4-chloro-3-indolyl-β-D-glucuronide monohexyl ammonium salt, Carl Roth GmbH) was added and vials were vacuum infiltrated on ice for 45 minutes. Staining was performed overnight in the dark at 37 °C. Samples were taken through the following ethanol series at room temperature: 20%, 30%, 50% ethanol, post fix solution (50% ethanol, 3.7% formaldehyde, 5% Acetic acid), 70%, 80% and 90% ethanol, with each step taking 30 minutes. Samples were kept in the fridge until fully cleared before sectioning with a vibratome as described above.

Quantitative image analysis

To quantify exocarp cell deformations, CLSM image stacks from case and control samples were loaded into MorphoGraphX software and the tissue surface detected and converted into a mesh by applying the edge-detect and marching cube algorithms (de Reuille et al., 2015). The surface-associated signal (2 - 6 µm) was

projected onto this mesh to allow for cell shape segmentation with the watershed 2D tool. Differences in cell length and width between samples were computed by applying the principal direction of growth algorithm on co-segmented cells. To determine differences in cell depth, cells were segmented with the watershed 3D tool and an ellipsoid was fitted to the extracted cell shape with the PCA algorithm with the smallest diameter of the ellipsoid representing the maximal depth of the cell. We computed cell volume and area by converting cell shapes into a 3D mesh and applying the heat map tool to quantify volume and area. Changes in exocarp cell length, width, volume, area and depth in response to osmotic treatments were calculated as described above, although over-segmentation was required in order to reconstruct correct 3D cell shapes in the fully plasmolyzed samples. Moreover, it was important to extract the surface area from segmented 3D cell shapes because the cuticle had swollen during salt treatment.

To quantify changes in exocarp tension *in situ* during *C. hirsuta* fruit development, CLSM image stacks of exocarp cells expressing a plasma membrane marker were acquired from intact fruit, and the same region of cells was re-imaged in water after the segment of valve was cut off the fruit. These image stacks were loaded into MorphoGraphX software and the main axes of deformation were quantified at the cellular scale using the algorithm normally used to compute principal directions of growth (de Reuille et al., 2015). Overall curling of the cut valve segment (about 1.5 mm long) was assessed by eye. Experiments were performed on fruits at five consecutive stages of development in two different plants.

To quantify the hinge angle of lignified endocarp *b* secondary cell walls, CLSM image stacks were acquired, imported into MorphoGraphX software and rotated such that a clipping plane was perpendicular to the major axis of the valve. Digital cross-sections in this clipping plane were exported as TIFF files and used to measure the geometry of the secondary cell wall in ImageJ (V1.46R). The angle between the lignified cell walls on two adjacent cell faces was measured as shown in Fig. 3B, C of the main text.

To quantify the degree and direction of cortical microtubule alignment, CLSM image stacks of *35S::GFP:TUA6* expression in exocarp cells were loaded into MorphoGraphX software and their surfaces extracted with the marching-cube algorithm (de Reuille et al., 2015). The surface mesh was subdivided into a fine mesh and the surface-associated signal (3-6 µm) was projected on to this mesh to allow for cell segmentation with the watershed 2D tool. The GFP signal was re-projected within a distance of 0.5-3 µm of the extracted surface, depending on the signal depth in the sample. The principal orientation of the GFP signal within each cell was computed using the Fibril orientations algorithm.

To measure the cross-sectional area of *C. hirsuta* endocarp *b* cells, transverse semi-thin plastic sections of valves from stage 14 to 17b were stained with toluidine blue and the cross-sectional area of endocarp *b* cells quantified using ImagePro software (MediaCybernetics).

<u>Cellular Force Microscopy (CFM)</u>

CFM was performed as previously described (Routier-Kierzkowska et al., 2012; Weber et al., 2015). Whole *C. hirsuta* fruits were immobilized on glass petri dishes with the valve facing upwards using strong waterproof tags (Tough Tags, Diversified Biotech, Dedham, USA) and submerged in water to avoid artifacts due to water loss or movements during measurements. To eliminate artifacts due to surface curvature (Routier-Kierzkowska et al., 2012), measurements were taken only on flat areas of the exocarp cells.

<u>Valve pulling experiment using custom-built extensometer</u>

One valve was first taken off the fruit and its diameter of curvature measured under a stereoscope. The rest of the fruit (including valve and replum) was then attached with strong adhesive tags on one end to a miniature load cell (LSB200, Futek, Advanced Sensor Technologies Inc.) and on the other end to a piezoelectric micropositioner arm (SLC-2475, SmarAct GmbH). The replum was then dissected off. The starting configuration ("zero load") is a curved valve (positioner arm and load cell are close to each other). The valve was then progressively stretched by increments of 50 µm. The stretching was stopped immediately by cutting the valve with scissors once the valve was visibly flat, in order to avoid further damage of the valve. The diameter of curvature of this pulled valve was then measured under a stereoscope. Experiments were filmed with a high magnification (300x) webcam. Scripts for the analysis of the force-displacement curves were written in Octave.

Modelling

1 Elastic energy – Tissue model

A mechanical model of the elastic energy in the valve was constructed by considering the valve as consisting of three distinct mechanical layers: a thin soft exocarp layer (exo), a soft middle mesocarp layer (meso), and the lignified stiff endocarp b layer (endo). The meso layer plays only a passive role, noncritical to the explosive mechanism. The mechanics is driven by a change in reference geometry of the exo layer, marked by a decrease in length and proportional increase in depth. Modelling details for the change in reference geometry are provided in Sec. 4. For the purposes of modelling in the present section, this change in geometry is taken as a fixed input, and we derive the energy landscape in the system to predict the degree of valve curvature when free from the replum and the amount of total elastic energy available for coiling. First, we describe the geometry of this tri-layer system.

1.1 Geometry

The tissue-level valve geometry is pictured schematically in Display item 1. We consider three distinct configurations of the valve. While attached to the replum, Config. I, the valve has a curved cross-section. Once off the replum, the valve must flatten before coiling, a transient state we refer to as Config. II. The length in Config II is the same as in Config I, with changes only occurring in the width and depth. Once flattened, the valve curls along its length until reaching an energy minimising state, Config. III. This involves changes in depth and length only, hence the width in Config III is equal to that in Config II.

In Config. I, let the width and depth of the layers be respectively given by $\hat{W}_i$ and $\hat{D}_i$, where $i = 1, 2, 3$ for the endo, meso, and exo layers, respectively. Similarly, let D_i denote the depths in Config. II, and d_i accordingly in Config. III. The length of all layers in Configs I and II is equal, given by L, while the width in all layers in Configs II and III is equal, given by W. We let l_i denote the lengths in Config. III and note that $l_1 = L$ due to the inextensibility of the lignified endo layer.

Let the cross-sectional curvature be given by U in Config. I and the axial curvature (both measured to the centre of the endo layer) in Config III given by u. Approximating the curved valve cross-sectional shape by the arc of an annulus, we note the relationships

$$\hat{W}_1 = \frac{\psi}{U}, \quad \hat{W}_2 = \psi(\frac{1}{U} + \delta_1), \quad \hat{W}_3 = \psi(\frac{1}{U} + \delta_1 + \delta_2), \tag{1}$$

where $\delta_1 = (\hat{D}_1 + \hat{D}_2)/2$ is the distance in depth between the centre of the endo and meso layers, and similarly $\delta_2 = (\hat{D}_2 + \hat{D}_3)/2$, and ψ is the arc angle as shown in Display item 1.

The coiling of the valve is driven by a change in reference geometry of the exo layer, marked by a decrease in length and an increase in depth. We define d_3^* and l_3^* as the reference depth and length for this layer. Similar variables could be defined for the width or for the other layers, but is unnecessary as no other change in reference geometry is observed. Hence the reference dimensions for the meso layer and exo width are taken as those in Config. I. Letting $g < 1$ denote the scale factor for the change in reference dimension, and assuming no growth or residual stress, we write $l_3^* = gL$, $d_3^* = \hat{D}_3/g$.

1.2 Energy

The mechanical energy consists of bending and stretching energies in the different layers. We approximate each layer as an incompressible hyperelastic cuboid with quadratic strain energy. For a cuboid with Young's modulus E, the stretching energy is given by

$$\frac{1}{2}\int_{\Omega} E\left(\lambda_1^2 + \lambda_2^2 + \lambda_3^2 - 3\right)\, d\mathbf{x}, \tag{2}$$

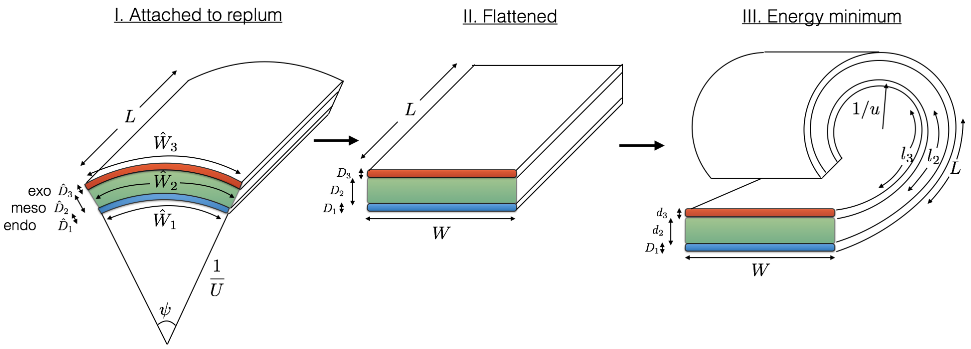

Display item 1: Valve geometry.

where integration is over the reference cuboid, and λ_i are the principal stretches. Assuming a uniform deformation from dimensions w^*, d^*, l^* to dimensions w, d, l, the principal stretches are simply the ratios of current to reference dimensions, and (2) simplifies to

$$\frac{EV}{2}\left(\left(\frac{w}{w^*}\right)^2+\left(\frac{d}{d^*}\right)^2+\left(\frac{l}{l^*}\right)^2-3\right). \tag{3}$$

where $V = wdl$ is the volume, assumed to be conserved in each layer through the deformation. Due to the inextensibility of the endo layer, stretching is negligible there, and the stretching energy is given by contributions of the meso and exo layers only. Explicitly, the stretching energy is

$$\mathcal{E}_{\mathrm{s}} = \frac{E_{\mathrm{meso}}V_2}{2}\left(\left(\frac{W}{\hat{W}_2}\right)^2+\left(\frac{d_2}{\hat{D}_2}\right)^2+\left(\frac{l_2}{L}\right)^2-3\right)+\frac{E_{\mathrm{exo}}V_3}{2}\left(\left(\frac{W}{\hat{W}_3}\right)^2+\left(\frac{d_3}{d_3^*}\right)^2+\left(\frac{l_3}{l_3^*}\right)^2-3\right). \tag{4}$$

where E_{meso} and E_{exo} are the Young's moduli for the meso and exo layers, respectively, and V_2 and V_3 are the respective volumes.

The bending energy for a layer with curvature $\tilde{u}$ is

$$\frac{EI}{2}\int_\Gamma \tilde{u}^2\, dS, \tag{5}$$

where I is the 2nd moment of area, equal to $wd^3/12$ for a rectangular cross-section, and integration is along the reference length. Note that curvature is taken with respect to reference arclength. For our simplified geometry with constant curvature in each layer and curvature u in the endo layer, the total bending energy is

$$\mathcal{E}_{\mathrm{b}} = \frac{E_{\mathrm{endo}}Wd_1^3}{24}Lu^2+\frac{E_{\mathrm{meso}}Wd_2^3}{24}Lu^2+\frac{E_{\mathrm{exo}}Wd_3^3}{24}\frac{(Lu)^2}{l_3^*}. \tag{6}$$

Note that the extra factor in the exo term accounts for the change in reference length in this layer.

1.3 Lignin geometry

The cross-sectional curvature in Config. I arises in part due to the growth of the seeds within the pod bowing out the valve. Once detached from the replum, the valve must first flatten in cross-section before lengthwise coiling can occur. The question is how this flattening affects the energy landscape. In particular, in the bowed state there is a width differential between outer and inner layers, e.g. $\hat{W}_3 > \hat{W}_1$ in Config I, whereas all widths are equal in Config II.

The hinged geometry of the lignin enables the endo layer to accommodate the flattening by opening the hinges and increasing the width of the endocarp cells to $\hat{W}_3$. Taking the reasonable assumption that the hinges can open/close passively, i.e. with negligible energy cost, then in terms of the endo and exo layers, the transition from Config I to II can occur at no energy cost. It is quite possible that there is in fact an energy gain, as the meso and exo layers may be flat in their stress-free states. The details of this depends on developmental specifics of the various cells. Such information is both very difficult

to ascertain and beyond the scope of our analysis, in part because the dynamics model for coiling that we develop in Sec. 8 assumes a fixed width (i.e. it considers the dynamics starting from Config II) and would be made unnecessarily complicated by including possible energy exchange from Config I to II.

Hence, for simplicity we assume in the case of hinged lignin that flattening occurs at no energy cost and that the width in Config II is given by $W = \hat{W}_3$. However, as illustrated schematically in Display item 2, in the case of *boxed lignin* the transition from Config I to II is not without energy cost. For one, the boxed lignin is unable to open and hence the endo layer cannot increase in width, so that the width in Config II is $W = \hat{W}_1$. Thus, the width differential must be accommodated by the meso and exo layers. In particular, the exo width must decrease and the exo depth must accordingly increase (since there is no length change from I to II). Recall that the exo layer geometry is such that the reference dimensions involve an increased depth and decreased length by a proportional factor $g < 1$, i.e. $l_3^* = gL$, $d_3^* = \hat{D}_3/g$. If the exo depth already increases during flattening while the length is unaffected, the result is that in coiling the exo layer is unable to reach its reference dimensions: if $d_3 = d_3^*$, we will have $l_3 > l_3^*$ while $l_3 = l_3^*$ implies $d_3 > d_3^*$. As a consequence, the energy landscape is not as favourable for coiling.

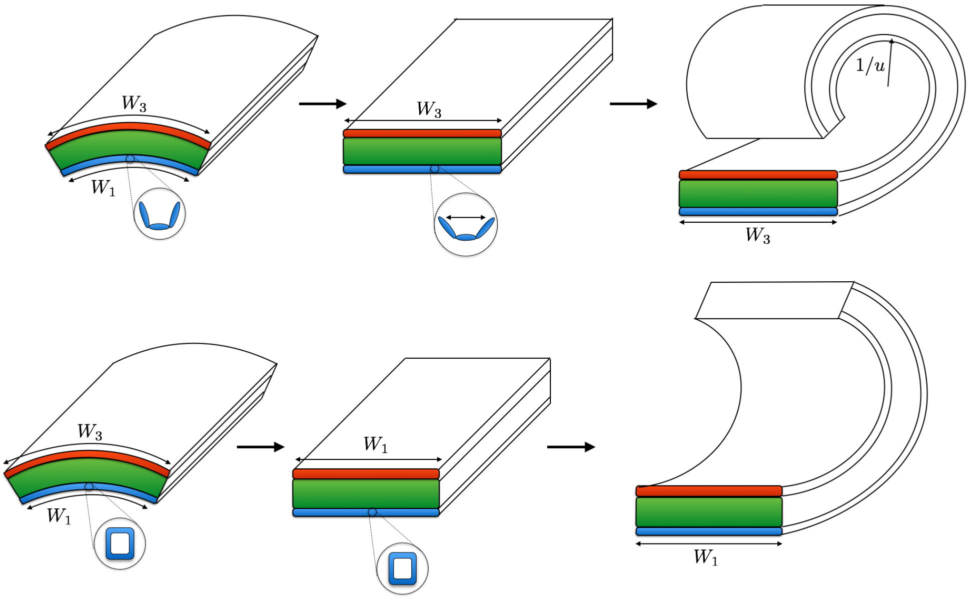

Display item 2: Hinged versus boxed lignin.

There is another effect of the boxed lignin geometry. We have observed that the lignin in the endo layer forms *after* the bowing of the valve. The formation of boxed lignin in an already curved valve stiffens the layer in a curved state, effectively "sealing in" the curvature. In this way, the endo layer can be thought of as having a nonzero reference cross-sectional curvature U that requires energy to flatten. Thus in Configs II and III, we surmise an additional "flattening" energy present in the case of boxed lignin only, given by

$$\mathcal{E}_{\mathrm{f}} = \frac{E_{\mathrm{endo}} L D_1^3}{24} W_1 U^2. \tag{7}$$

1.4 Energy minimization

The geometrical arguments above suggest that a hinged geometry is energetically favorable for explosive coiling. To quantify the analysis, we determine the energy minimising state and the change in stored energy between the different configurations. We take as input the geometry in Config I, i.e. the parameters $\{\hat{W}_i, \hat{D}_i, L\}$ for $i = 1, 2, 3$ (subject to the constraints of Eqn (1)), the change in reference proportion for the exo layer, g, and the material parameters E_{exo}, E_{meso}, E_{endo}. As discussed above, the width in Config II and III is given by $W = \hat{W}_3$ in the hinged case and $W = \hat{W}_1$ in the boxed case. Since the length for all layers is L in Config II, the depths D_i in Config II are also known.

As the endo width and depth do not change from Config II to III, the remaining unknowns are the meso and exo depths and lengths as well as the curvature u. Geometry enables us to write the depth in

Parameter	Description	Value
$\hat{W}_1$	endo width on replum	0.94 mm
$\hat{W}_2$	meso width on replum	0.98 mm
$\hat{W}_3$	exo width on replum	1.04 mm
$\hat{D}_1$	endo depth on replum	15 μm
$\hat{D}_2$	meso depth on replum	85 μm
$\hat{D}_3$	exo depth on replum	20 μm
L	length on replum	18 mm
g	exo geometry ratio	0.8
E_{exo}	Young modulus exo layer	16.5 MPa
E_{meso}	Young modulus meso layer	16.5 MPa
E_{endo}	Young modulus endo layer	4.5 GPa

Display Table 1: Model parameter values for *C. hirsuta* wild type.

terms of the curvature u, as follows: Since u is the curvature measured to the center of the endo layer, simple geometry for circular arcs gives the expression

$$Lu(\frac{1}{u} + \delta) = l_2, \tag{8}$$

where $\delta = (d_1 + d_2)/2$ is the distance in depth between the centre of the endo and meso layers. By volume conservation we have $l_2 = D_2L/d_2$, thus (8) provides a quadratic equation that can be solved to give d_2 as a function of u. A similar calculation yields $d_3 = d_3(u)$. Then another application of volume conservation gives $l_2 = l_2(d_2(u))$ and similarly for l_3. In this way, for both the boxed and hinged geometries, the total energy can be expressed as a function of the single variable u, the curvature in Config III. The energy function expresses a single minimum value that defines Config III, and note that $u = 0$ corresponds to the flat state, Config II. We define the *coiling energy* as the drop in energy from Config II to Config III. Having computed the energy minimising curvature, the number of coils N is obtained from the relation

$$\frac{2\pi N}{u} = L. \tag{9}$$

1.5 Parameters

Parameter values for the wild type are provided in Display table 1. Geometric parameters were obtained by measuring whole fruits and fruits freshly sectioned with a vibratome as described in materials and methods. The Young's modulus of the endo layer is based on the stiffness of lignin and an approximation of the proportion of the endo layer that is lignified, roughly 50% for the wild type.

It remains to estimate the Young's modulus of the exo layer and meso layers. Being unlignified, both layers were assumed to have a comparable stiffness, thus we set $E_{\text{meso}} = E_{\text{exo}}$. An important feature of the energy landscape we have observed is that the location of the energy minimum is robust to changes in the exo stiffness. Thus, the curvature in Config III is essentially unchanged by varying E_{exo}. However, the energy difference between Config II and III, that is the amount of energy released in coiling, is strongly affected. A 10-fold difference in E_{exo} produces a nearly 10-fold difference in energy.

Thus, E_{exo} plays an important role in the coiling timescale. To obtain an estimate of E_{exo} we utilised a multi-scale approach, connecting our tissue-level model to an organ-level model of the valve that was matched to an experiment on the whole valve. This is described in Sec. 2, and the determined value of E_{exo} is reflected in Display table 1.

1.6 Hinged vs boxed lignin

The parameters for the transgenic plants *C. hirsuta* NST3::VND7 with boxed lignin are given in Display table 2. Aside from the boxed lignin, these valves showed an increased depth of lignified cells, but with proportionally less lignin, hence an increased value of $\hat{D}_1$ compared to the wild type but a decreased stiffness E_{endo}. The energy profile is given in Main text, Fig. 3E. Compared to the Wild type, the model predicts approximately 80% less coiling energy and a decreased curvature, with approximately

Parameter	Description	Value
$\hat{W}_1$	endo width on replum	0.94 mm
$\hat{W}_2$	meso width on replum	1.0 mm
$\hat{W}_3$	exo width on replum	1.04 mm
$\hat{D}_1$	endo depth on replum	50 μm
$\hat{D}_2$	meso depth on replum	50 μm
$\hat{D}_3$	exo depth on replum	20 μm
L	length on replum	18 mm
g	exo geometry ratio	0.8
E_{exo}	Young modulus exo layer	16.5 MPa
E_{meso}	Young modulus meso layer	16.5 MPa
E_{endo}	Young modulus endo layer	1 GPa

Display Table 2: Parameter values for transgenic plant with boxed lignin *C. hirsuta* NST3::VND7.

Valve type	Coiling energy (mJ)	Number of coils	Endo flattening energy (mJ)
Wild type	0.47	3.5	0
Boxed transgenic	0.1	1.5	0.14
Boxed wild type	0.11	1.5	0.01

Display Table 3: Energy summary for different valve simulations, comparing the Wild Type, the transgenic plants *C. hirsuta* NST3::VND7 with boxed lignin, and a hypothetical transgenic plant equivalent to the Wild Type except for having boxed lignin (final row). Number of coils is computed per 18mm length.

1 coil in Config III. Moreover, the energy cost associated with flattening the cross-sectional reference curvature of the endo layer in Config I is 0.14 mJ, nearly 3 times the coiling energy. Note that due to this extra component of the energy not present in wild type, the total energy in the system is higher in the simulated transgenic valve. Effectively, most of the energy is "trapped" due to the boxed geometry, so that only a fraction is available for coiling.

The comparison between the wild type and transgenic is summarised in Display table 3. Since these two plants do not differ *only* in the lignin geometry (as stated the total depth of lignified cells, referred to as the endo depth, is greater in the transgenic), we also simulated a valve with identical dimensions to the wild type, but with boxed lignin. The result is also displayed in Display table 3: these valves again showed a significantly decreased coiling energy and decreased curvature in Config III, though both were slightly higher than the transgenic valve. These factors, in particular the slight energy cost in flattening, make it unlikely that such a valve would exhibit explosive pod shatter.

2 Valve pulling experiment

2.1 Organ level model

We model the pulling experiment at the organ level, treating the valve as a single elastic rod with fixed intrinsic curvature. The intrinsic curvature is measured experimentally and is assumed to be a known parameter in the model. Due to the formation of "kinks" in the relaxed state of the clamped valve, and supposing that the kink points act merely as a hinge, so that the valve has zero moment at these points, then mechanically the situation is equivalent to pulling on an inextensible beam with intrinsic curvature and zero moment (pinned) boundary conditions. This situation is described by the elastica equations:

$$\begin{aligned} x' &= \cos\theta \\ y' &= \sin\theta \\ m' &= n_x \sin\theta - n_y \cos\theta \\ m &= E_b(\theta - \hat{u}) \end{aligned} \tag{10}$$

**Data Set**	$\hat{u}$ (mm^{-1})	$E_b = K$ (Nmm^2)	E_{exo} (MPa)	g
V1	0.58	0.0075	11.9	0.85
V2	0.91	0.0046	5.8	0.74
V3	0.86	0.0092	14.3	0.79
V4	0.90	0.0072	10.6	0.78
V6	0.90	0.0035	3.8	0.72

Display Table 4: Best fit values of $\hat{u}$ and K, using high curvature measurement, to match organ-level model with pulling experiment, and the corresponding values of E_{exo} and g when translating to tissue-level trilayer model.

**Data Set**	$\hat{u}$ (mm^{-1})	$E_b = K$ (Nmm^2)	E_{exo} (MPa)	g
V1	0.34	0.0187	34.1	0.92
V2	0.57	0.009	14.5	0.86
V3	0.66	0.0143	24.3	0.85
V4	0.57	0.0144	24.8	0.87
V6	0.36	0.012	20.7	0.91

Display Table 5: Equivalent values as in Display table 4, using low curvature fit.

along with boundary conditions

$$\begin{aligned} &y(0) = y(L) = 0 \\ &m(0) = m(L) = 0 \\ &x(0) = 0, \;\; x(L) = L - \Delta L. \end{aligned} \tag{11}$$

Here all variables are functions of the fixed arclength $s \in (0, L)$, where L is the length of the valve, $(x(s), y(s))$ is the position of material point s, θ is the angle between the tangent and the x-axis, m is the moment, $\hat{u}$ the intrinsic curvature of the bilayer, and E_b the effective bending stiffness of the whole valve, the product of Young's modulus and 2nd moment of area. The stress in the beam is given by the constant vector (n_x, n_y), but symmetry implies $n_y = 0$; we are left with the unknown n_x, which is the tensile force measured by the extensometer in the pulling experiment and is a function of the imposed end displacement ΔL. Under a small angle linearisation, $|\theta| \ll 1$, (10) simplifies to

$$\begin{aligned} &\theta'' - c^2\theta = 0 \\ &\theta'(0) = \theta'(L) = \hat{u}, \end{aligned} \tag{12}$$

where $c = \sqrt{n_x/E_b}$. This is easily solved exactly, from which the end displacement condition yields the following relation:

$$\Delta L = \frac{\hat{u}^2(\sinh(cL) - cL)}{4c^3 \cosh(cL/2)^2}. \tag{13}$$

For given values of length L and curvature $\hat{u}$, this relation defines a force displacement curve, n_x vs ΔL. The only free parameter is the stiffness E_b, as the length and curvature are measured in the experiment. However, two different values of curvature $\hat{u}$ were reported, one for the freshly exploded half of the valve and one measured after the pulling experiment. The latter gave a smaller curvature value, likely due to "tiring" of the valve from pulling. Excellent fits with the data could be obtained with either value. Hence, we have fit E_b to the data twice, using both the high and low curvature values.

The fitting exercise was conducted for five different data sets, with the best fit value of E_b given in Display tables 4 (high curvature) and 5 (low curvature). The next step is to determine the bending stiffness of the exo layer from the effective bending stiffness of the whole valve, which requires a tissue-level analysis of the trilayer.

2.2 Tissue level model

At the tissue level, we return to the trilayer energy of Sec 1, and use the effective stiffness determined at the organ level to estimate the Young's modulus of the exo and meso layers. The energies described

in Sec 1 correspond to linear constitutive laws. Consider a bilayer structure, letting a '+' superscript denote a quantity in the outer layer, and equivalently a '-' for the inner layer. The constitutive laws are

$$\begin{aligned} n^+ &= k^+(\alpha^+ - 1) \\ m^+ &= K^+ u^+ \\ m^- &= K^- u^-, \end{aligned} \tag{14}$$

where n^+ is the inner layer axial stress, a linear function of the axial stretch. Note the inner '-' layer will ultimately correspond to the inextensible endo layer; hence there is no constitutive law for n^-, as it is instead replaced by the geometrical constraint of inextensibility. Here α^+ is the axial stretch, equal to the ratio of current to reference length in the '+' layer, k^+ is the axial stiffness coefficient, and $m^\pm$ are the bending moments, linear functions of curvature with bending stiffness coefficients $K^\pm$.

To map to the organ level model, we follow the ideas in Lessinnes et al. (2015) and use the fact that two planar elastic rods with constitutive laws of the form (14) are mechanically equivalent to a single elastic rod with constitutive law

$$M = K(u - \hat{u}), \tag{15}$$

where the effective stiffness and intrinsic curvature, K and $\hat{u}$ respectively, are given by

$$K = \frac{1}{g}\left(K^+ + gK^- + \delta^2 k^+\right), \quad \hat{u} = \frac{\delta k^+(1-g)}{K^+ + gK^- + \delta^2 k^+}. \tag{16}$$

In these expressions g is the length differential between the reference lengths, i.e. $g = l^*/L$ in the parlance of Sec 1, and δ is the distance in depth between each layer. Note that the effective rod is defined with centreline at the centre of the inextensible '-' rod. Since our tissue model for the mechanics in fact utilizes a trilayer, we must apply these equations twice. We take as input the dimensions of endo, meso, and exo layers, as well as endo Young's modulus (following Display table 1). For a layer with width w, depth d, and Young's modulus E, the stiffness parameters are $k = Ewd$, $K = Ewd^3/12$. Hence, in terms of the $K^\pm$, the only unknown is $E_{\text{meso}} = E_{\text{exo}}$. The distance between layers, δ, was computed using the depths D_i in Config II. We first determined the effective stiffness of the endo and meso layers by applying Eqns (16) with $g = 1$ (since no reference length differential exists between meso and exo layers). Hence for these two layers $\hat{u} = 0$ and $K := K_{1,2}$ depends on the as yet unknown E_{exo}. We then considered the effective stiffness of the whole valve by applying Eqns (16) again, with $K^- = K_{1,2}$ corresponding to the effective meso+endo layer and k^+, K^+ corresponding to the exo layer. Here we take K and $\hat{u}$ as the best-fit values from the organ-level model. Then Eqns (16) provide two equations to solve for the two unknowns g and E_{exo}.

For each data set we applied the above procedure to solve for g and E_{exo}. The resulting values are given as the final two columns in Display tables 4 and 5. The average values from the 5 data sets were

$$E_{\text{exo}} = 9.3\text{MPa}, \quad g = 0.78. \tag{17}$$

for the high curvature, and

$$E_{\text{exo}} = 23.7\text{MPa}, \quad g = 0.88. \tag{18}$$

for the low curvature. The value of g corresponds well with the independent experimental measure of $g = 0.8$ as represented in Display table 1. The average of the two computed Young's moduli, 16.5MPa, is reflected in Display table 1 and was used for subsequent computations. The Young's modulus was validated against the cellular-level model, as discussed in Sec.7.

3 Mechanical model of 3D cellular plant tissue

In order to understand the change in geometry in the exocarp cell layer that drives the mechanics of explosive seed dispersal, we constructed a mechanical model of *C. hirsuta* exo cells. 3D cells were created as a staggered array of boxes (Display item 3a) representing an idealised geometry for the exocarp-layer. Each 3D cell was represented by a closed triangular surface mesh using the vertex-vertex data structure (Smith et al., 2004). Nodes on the walls between cells were shared. Because of the symmetry of the problem, only one quarter of the template was simulated. All models were developed under the Virtual Laboratory modelling environment, (Federl and Prusinkiewicz, 1999).

The mechanical simulation was performed with a GPU-accelerated, explicit finite element method which extends the model of Bassel et al. (2014) to handle anisotropy. Triangles representing sections

of cell wall were modelled as membrane elements with a linear, hyperelastic, transverse isotropic, St. Venant material law (Holzapfel, 2000; Bonet and Burton, 1998). For the membrane elements we used plane stress and a zero transversal shear strain hypothesis (for a modified version of the Kirchhoff-Love plate theory see Chapelle and Bathe (2003)).

Assuming the fibre direction in the y axis, so that x and z are the isotropic axes, the elasticity tensor for the transversely isotropic St. Venant model in Voight notation is

$$\mathcal{S} = \begin{pmatrix} \frac{E(1-n\tilde{\nu}^2)}{m(1+\nu_z)} & \frac{E_y\tilde{\nu}}{m} & \frac{E(\nu_z+n\tilde{\nu}^2)}{m(1+\nu_z)} & 0 & 0 & 0 \\ \frac{E_y\tilde{\nu}}{m} & \frac{E_y(1-\nu_z)}{m} & \frac{E_y\tilde{\nu}}{m} & 0 & 0 & 0 \\ \frac{E(\nu_z+n\tilde{\nu}^2)}{m(1+\nu_z)} & \frac{E_y\tilde{\nu}}{m} & \frac{E(1-n\tilde{\nu}^2)}{m(1+\nu_z)} & 0 & 0 & 0 \\ 0 & 0 & 0 & 2G & 0 & 0 \\ 0 & 0 & 0 & 0 & \frac{E}{1+\nu_z} & 0 \\ 0 & 0 & 0 & 0 & 0 & 2G \end{pmatrix} \tag{19}$$

where

$$n = E_y/E, \quad m = 1 - 2n\tilde{\nu}^2 - \nu_z \tag{20}$$

and E_y is the Young modulus along the fibers, E is the Young modulus in the orthogonal plane (which is isotropic), $\tilde{\nu} = \nu_{xy} = \frac{\nu_{yx}}{n}$. For small strains -$\nu_{xy}$ determines (at first order) the strain induced in the fibre direction because of a strain in one of the isotropic directions. The same holds for ν_{yx}, but with the directions inverted. ν_z is Poisson ratio in the isotropic plane and G is the shear modulus between the fibre direction and the isotropic plane. All the symmetry properties of the material have been exploited in the writing of the matrix. The II Piola-Kirchhoff stress tensor S is then

$$S_{ij} = \mathcal{S}_{ijkl}\mathcal{E}_{kl} \tag{21}$$

where $\mathcal{E}$ is the Green-Strain tensor. Expanded, this becomes

$$\begin{pmatrix} S_{xx} \\ S_{yy} \\ S_{zz} \\ S_{xy} \\ S_{xz} \\ S_{yz} \end{pmatrix} = \begin{pmatrix} \frac{E(1-n\tilde{\nu}^2)}{m(1+\nu_z)} & \frac{E_y\tilde{\nu}}{m} & \frac{E(\nu_z+n\tilde{\nu}^2)}{m(1+\nu_z)} & 0 & 0 & 0 \\ \frac{E_y\tilde{\nu}}{m} & \frac{E_y(1-\nu_z)}{m} & \frac{E_y\tilde{\nu}}{m} & 0 & 0 & 0 \\ \frac{E(\nu_z+n\tilde{\nu}^2)}{m(1+\nu_z)} & \frac{E_y\tilde{\nu}}{m} & \frac{E(1-n\tilde{\nu}^2)}{m(1+\nu_z)} & 0 & 0 & 0 \\ 0 & 0 & 0 & 2G & 0 & 0 \\ 0 & 0 & 0 & 0 & \frac{E}{1+\nu_z} & 0 \\ 0 & 0 & 0 & 0 & 0 & 2G \end{pmatrix} \begin{pmatrix} \mathcal{E}_{xx} \\ \mathcal{E}_{yy} \\ \mathcal{E}_{zz} \\ \mathcal{E}_{xy} \\ \mathcal{E}_{xz} \\ \mathcal{E}_{yz} \end{pmatrix} \tag{22}$$

Triangular membrane elements allow the nodal force calculations to be performed in 2D by rotating each deformed triangle onto the xy-plane. Reference triangles are constructed from rest lengths of edges stored in the triangular mesh, and rotated so that their fiber direction is aligned with the y axis. The non null components of the Green-Strain tensor in the plane are, in turn, evaluated as:

$$\begin{aligned} \mathcal{E}_{xx} &= \left(D_x[1]\boldsymbol{u} + 0.5(D_x\boldsymbol{u})^T(D_x\boldsymbol{u})\right) \\ \mathcal{E}_{yy} &= \left(D_y[2]\boldsymbol{u} + 0.5(D_y\boldsymbol{u})^T(D_y\boldsymbol{u})\right) \\ \mathcal{E}_{xy} &= 0.5\left(D_x[1]\boldsymbol{u} + D_y[2]\boldsymbol{u} + (D_x\boldsymbol{u})^T(D_y\boldsymbol{u})\right) \end{aligned} \tag{23}$$

D_x and D_y are defined as

$$D_x = \begin{pmatrix} \frac{\partial\phi_1}{\partial x} & 0 & \frac{\partial\phi_2}{\partial x} & 0 & \frac{\partial\phi_3}{\partial x} & 0 \\ 0 & \frac{\partial\phi_1}{\partial x} & 0 & \frac{\partial\phi_2}{\partial x} & 0 & \frac{\partial\phi_3}{\partial x} \end{pmatrix} \tag{24}$$

$$D_y = \begin{pmatrix} \frac{\partial\phi_1}{\partial y} & 0 & \frac{\partial\phi_2}{\partial y} & 0 & \frac{\partial\phi_3}{\partial y} & 0 \\ 0 & \frac{\partial\phi_1}{\partial y} & 0 & \frac{\partial\phi_2}{\partial y} & 0 & \frac{\partial\phi_3}{\partial y} \end{pmatrix} \tag{25}$$

where ϕ_i are the (linear) hat basis functions. We will use $D_i[j]$ to indicate operator D_i, row j. Under the plane stress hypothesis ($S_{zz} = 0$) the strain connected to the change in thickness of the membrane element can be computed as follows:

$$\mathcal{E}_{zz} = -\frac{(\mathcal{S}_{zzxx}\mathcal{E}_{xx} + \mathcal{S}_{zzyy}\mathcal{E}_{yy})}{\mathcal{S}_{zzzz}} \tag{26}$$

The nodal forces $\mathbf{F}$ on a triangle due to its deformation were calculated as

$$\begin{pmatrix} F_x^1 \\ F_y^1 \\ F_x^2 \\ F_y^2 \\ F_x^3 \\ F_y^3 \end{pmatrix}^T = \begin{array}{c} \Big(\left(D_x[1] + ({D_x}^T D_x \boldsymbol{u})\right) S_{xx} + \left(D_y[2] + ({D_y}^T D_y \boldsymbol{u})\right) S_{yy} + \\ \left(D_x[2] + D_y[1] + ({D_x}^T D_y + {D_y}^T D_x)\boldsymbol{u}\right) S_{xy} \Big) tA \end{array} \tag{27}$$

where $\boldsymbol{u}$ is the vector of nodal displacements, t is the element thickness and A is the area of the triangle in the undeformed configuration. Forces were then rotated back to the global configuration and the contribution from pressure added to the nodes in the direction of the triangle normal. Pressure was defined to be constant in all cells. The forces were then summed over the nodes and a pseudo-time stepping method was used to reach mechanical equilibrium. The process was repeated until the sum of the squared forces on the nodes was smaller than a prescribed tolerance. Integration was performed with backward Euler, with the resulting nonlinear system linearised with Newton's method and solved with a GPU-based Krylov solver. Calculations were performed using an nVidia GeForce Titan graphics card that has the equivalent of 896 double-precision cores. GPU operations were coded using the Thrust toolkit (http://docs.nvidia.com/cuda/thrust/index.html).

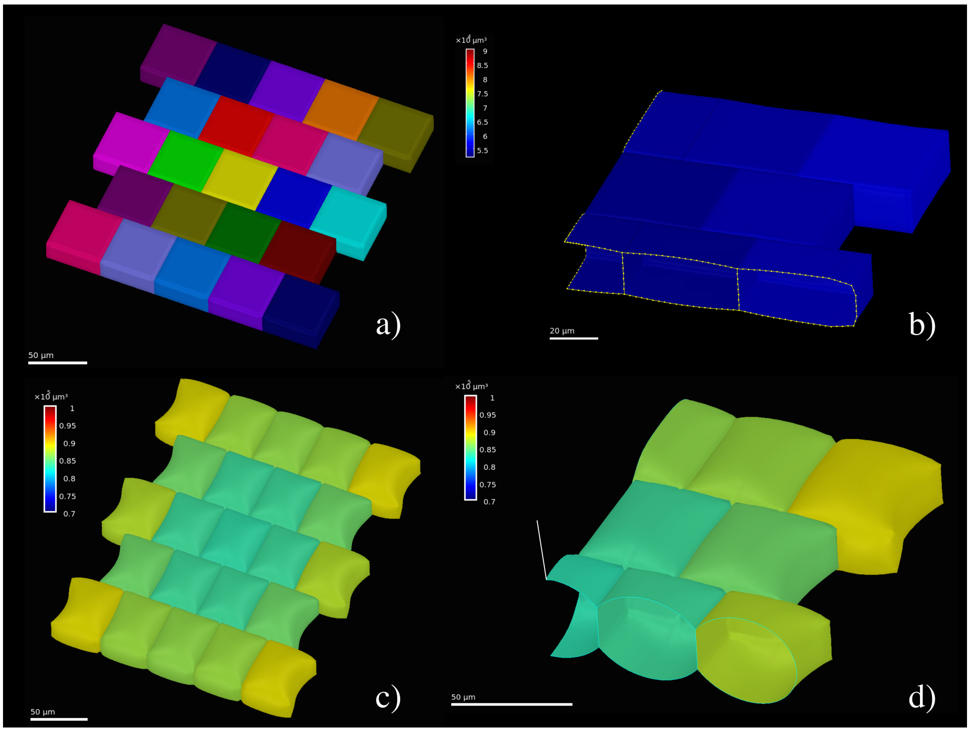

Display item 3: Template for finite element model of a *C. hirsuta* valve. Colorbar represents cells volume in μm^3. For cut cells we used the volume of the corresponding whole cell a) Initial cell template. b) Template after slight pressurization to obtain more realistic cell shapes. Due to symmetry, only $\frac{1}{4}$ of the template is required for the simulation. c) Fully pressurized template before indentation. d) Template after indentation.

4 Model of turgor driven shrinkage

3D cellular templates were created with dimensions taken from averaged values for *C. hirsuta* valve exocarp cells at two different developmental stages (Display Table 6). In order to have a zero-turgor (stress-free) template with a more realistic shape, the initial templates were pressurised slightly (Display item 3b). This gave a slight curvature to the cell walls that more closely matched the zero-pressure (plasmolised) state (Main text Fig. 5B). The slightly deformed cell template was then used as the stress-free starting point (reference configuration) for further simulation. Pressure inside the cells was then applied to the template (Display item 3c) and the parameters for the two Young's moduli (E and E_y) adjusted to approximate the deformation data obtained from osmotic treatments (Supplementary Table S1). This allowed us to estimate how much anisotropy was required in order to match the length shrinkage and volume increase of the cells. Note that these values are approximations, as the cell wall material most likely exhibits non-linear behaviour, and we are approximating it with a linear material model. The disadvantage of choosing a non-linear material model is that the parameter interpretation becomes more complex. Display Table 7 shows the model is much less sensitive to changes in anisotropy than to changes in pressure. Volume increase was calculated from the template centre cell, and length increase from the distance between the centres of the central cell and the cell adjacent to it in the fibre direction , in order minimise any effect from boundary conditions.

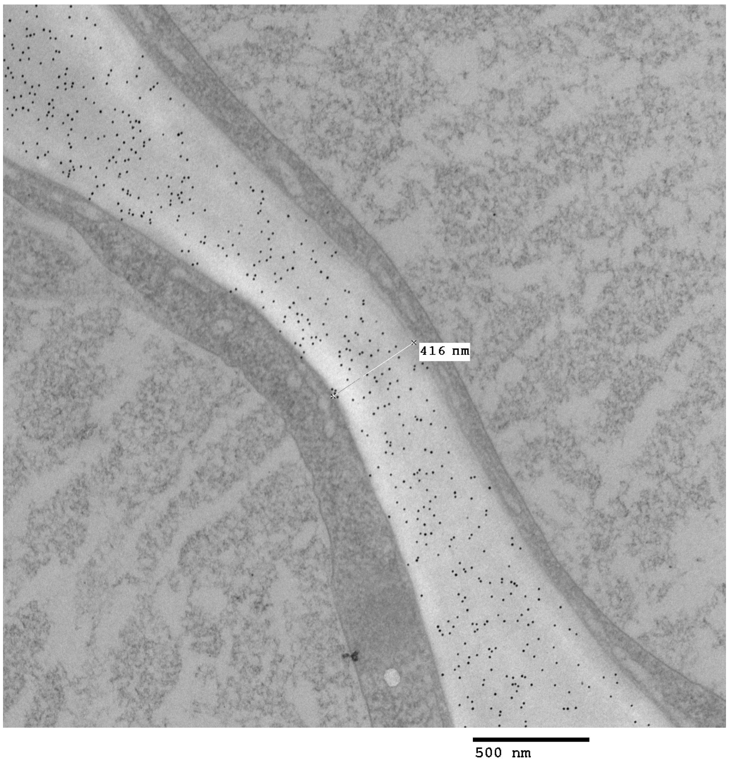

Display item 4: Transmission electron micrograph of two cell walls between adjacent exocarp cells in *C. hirsuta* fruit. The pectin component of the cell wall is labeled by immuno-gold using the JIM7 antibody. The average thickness for two adjacent cell walls (N=53) was halved to give an average cell wall thickness of $0.203\mu m \pm 0.007\ \mu m$ (standard error of mean).

We set the Poisson's ratio in the isotropic case to $\nu = .4$. In the anisotropic case $n\tilde{\nu} = \nu_{yx}$ was set to 0.4, while the Poisson's ratio in the isotropic plane ν_z was set to .8 so that the total cell wall compressibility was comparable to the isotropic case. The shear modulus is not an independent parameter in the isotropic case. For the anisotropic case we set $G = \frac{E}{2(1+n\tilde{\nu})}$, so to be the same as for an isotropic material with Young's modulus E and Poisson's ratio $\nu = 0.4$. A list of simulation parameter values is given in Display Table 6.

Parameter	Symbol	Value	Main text Figure
All simulations			Fig. 5, 7
Thickness [1]	t	.2μm	
Mesh element size	$l \times w$	2μm x 2μm	
C. hirsuta Stage 15:			
Pressure	P	0.7 MPa	
Young's modulus	E	600 MPa	
Poison's ratio	ν	.4	
Cell size	$l \times w \times h$	30μm $\times$ 20μm $\times$ 14μm	
C. hirsuta Stage 17b:			Fig. 5G
Pressure	P	0.65 MPa	
Young's moduli	E	150 MPa	
	E_y	9000 MPa	
Shear modulus	G	54 MPa	
Poison's ratio [2]	$\tilde{\nu}$	$.4/n = 0.0067$	
Poison's ratio (iso plane)[2]	ν_z	.8	
Cell size	$l \times w \times h$	50μm $\times$ 50μm $\times$ 20μm	
Long cells, isotropic			Fig. 5D
Pressure	P	0.7 MPa	
Young's modulus	E	300 MPa	
Poison's ratio	$\tilde{\nu}$	0	
Cell size	$l \times w \times h$	100μm $\times$ 20μm $\times$ 20μm	
Square cells, isotropic			Fig. 5E
Pressure	P	0.7 MPa	
Young's modulus	E	300 MPa	
Poison's ratio	$\tilde{\nu}$	0	
Cell size	$l \times w \times h$	50μm $\times$ 50μm $\times$ 20μm	
Square cells, anisotropic			Fig. 5F
Pressure	P	0.7 MPa	
Young's moduli	E	200 MPa	
	E_y	4800 MPa	
Shear modulus	G	96 MPa	
Poison's ratio [2]	$\tilde{\nu}$	$.4/n = 0.017$	
Poison's ratio (iso plane)[2]	ν_z	.78	
Cell size	$l \times w \times h$	50μm $\times$ 50μm $\times$ 20μm	

Display Table 6: Parameter values for FEM simulations.
[1]See Display item 4.
[2]Values set so that compressibility (total change in material volume) is comparable to isotropic case.

5 Cellular Force Microscopy (CFM)

CFM measurements were performed as described previously (Weber et al., 2015). Stiffness was measured by a straight-line fit of the force indentation curve from an indentation depth of approximately 0.5-1 μm (see Display item 5). As is typical with CFM measurements, there was a slight difference between the indentation and retraction stiffness, so the average of the two stiffness values was used. For every fruit sample about 5 to 8 average sized exocarp cells were selected for CFM experiments (Display Table 8), and 3 to 12 indentations were performed on each cell. Stiffness values were averaged over all cells measured in a single fruit sample. Measurements were discarded when force-indentation curves showed sudden drops in force during the indentation or exhibited other non-linear behaviour, or more than a 33% difference in stiffness between the indentation and retraction phase. With these criteria about 60%

Parameter change	E	E_y	P	Volume ratio	Length ratio	Reaction force	Stiffness
Actual				1.53	.88	20	24
Fitted model	150	9000	0.65	1.53	0.91	22.21	24.58
E_y 15% less	150	7650	0.65	1.53	0.91	22.25	24.46
E_y 15% more	150	10350	0.65	1.53	0.91	22.54	25.05
E_y 50% less	150	4500	0.65	1.55	0.92	21.5	23.55
E_y 50% more	150	13500	0.65	1.52	0.91	22.59	25.1
P 15% less	150	9000	0.55	1.49	0.91	19.6	21.44
P 15% more	150	9000	0.74	1.57	0.91	24.97	27.37

Display Table 7: Sensitivity analysis of stage 17b fruit model to changes in anisotropy E_y, and pressure P. All other parameters as in Display Table 6.

of the indentations were used to calculate average stiffness values (Display Table 9).

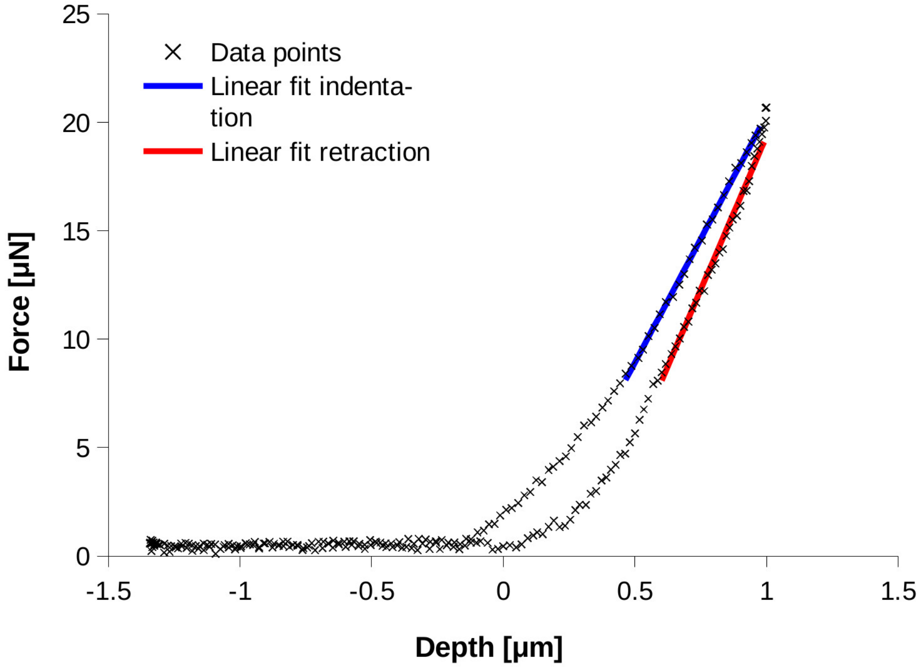

Display item 5: Typical force-indentation curve obtained from a CFM indentation experiment. The zero-point on the x-axis denotes the sample surface. To calculate the stiffness, a linear fit was made to the data points between 8 and 20 μN. This was done independently for the indentation and retraction phase.The average of these values was then used for the stiffness.

6 Modeling the Cellular Force Microscopy (CFM) experiments.

Even with the above assumptions for Poisson's ratios and the shear moduli, the model still has too many free parameters to fit volume and length shrinkage data, since the pressure is unknown. To address this, measurements were made with the CFM which is known to be highly sensitive to pressure (Weber et al., 2015; Routier-Kierzkowska et al., 2012). The CFM experiment was simulated as follows. After the initial pressurisation of the template converged, the bottom of the template was fixed in z and

Stage	Length	Width	Depth	n
Early	28.9 ± 4.55 μm	19.6 ± 0.42 μm	14 μm (est)	11
Late	46.7 ± 12.1μm	47.8 ± 1.84 μm	18 μm (est)	7

Display Table 8: Summary of exocarp cell size for CFM measurements on C. hirsuta fruits. ± indicates standard deviation and n indicates the number of fruit samples. Early developmental stage corresponds to stage 15 (5mm fruit length), and late is stage 17 (17mm fruit length). Depth values were estimated from optical sections.

Stage	Indentation Stiffness	Retraction Stiffness	n	Average Stiffness
Early	16.5 ± 1.82 N/m	21.1 ± 3.60 N/m	32	19 N/m
Late	20.8 ± 1.97 N/m	28.8 ± 3.92 N/m	50	24 N/m

Display Table 9: Summary of stiffness measurements on C. hirsuta fruits. ± indicates standard deviation and n indicates the number of fruit samples. Early developmental stage corresponds to stage 15 (5mm fruit length), and late is stage 17 (17mm fruit length).

then indentation was simulated by displacing a central node on the upper surface of the template in the z direction (Display item 3d). Previous studies have shown that for turgid cells, the indenter size does not affect the reaction force if the indenter is small (Weber et al., 2015). The displacement was performed in small steps, with the force recorded after mechanical equilibrium was reached for each step. This produced a force-indentation curve similar to that obtained from the CFM experiments (see Display item 5). The force-indentation curves from CFM experiments were more linear after significant indentation than during contact (see Display item 5). Therefore we used the stiffness after significant indentation, calculated as the difference in force at the depths $.5\mu m$ and $1.5\mu m$. The use of stiffness rather than absolute force to fit the model is more robust since it avoids non-linearities in force during contact and the associated uncertainty of the exact contact point. With the addition of stiffness data from CFM experiments, there were then three experimental measurements, stiffness, length decrease, and volume increase, to fit to the three model parameters, two Young's moduli and the pressure. To ensure that the results were independent of the mesh discretisation, we performed mesh refinement until the results differed by less than 2.5% between successive refinement steps. This resulted in a mesh with approximately 3600 triangles.

7 Connection with macroscopic model of seed launch

In order to compare the cellular model with the tissue level model of explosive seed dispersal, we investigated how much tension the model would predict the valve to have in the outer layer before explosion. To achieve this, the ends of the model were fixed in y before pressurisation to prevent any decrease in the length of the structure. We then pressurised the model and recorded the forces on the ends after the system reached mechanical equilibrium. Using the cell size parameters in Display Table 6 for the stage 17b fruit, the pulling force for the complete exo layer was computed as 61 mN.

In Display Table 7 the results for different parameters are shown. Although the stiffness ratio, defined as E_y/E weakly affects the fitted parameters for the CFM indentation experiments (see Main text Fig. 5G-I), here it plays a more significant role.

To compare this result with the tissue level model, we computed the force needed to keep a layer of cells 1mm wide by 20 μm deep at their original length, if they have shrunk by 10%. Following the constitutive law for n^+ in Eqn (14) and using the average of the computed exo Young's moduli, the force is computed as

$$n = E_{\text{exo}} wd(\alpha - 1) \approx 16.5\text{MPa} \times 1000\mu\text{m} \times 20\mu\text{m} \times (\frac{1}{0.9} - 1) \approx 36.6\text{mN}. \qquad (28)$$

While this average value is lower than that from the cell model computation, it is the same order of magnitude and the cell value is within the range of computed tissue values (e.g. the computed force

E_y	Reaction force on the tissue end face (mN)
9000	61
7650 (15% decrease)	57 (9% decrease)
10350 (15% increase)	65 (6% increase)

Display Table 10: Pulling force on the outer layer of a *C. hirsuta* valve obtained by preventing length increase by fixing the ends of a line of cells. All parameters are the same as in Display Table 6, except E_y.

using the V1 data in Table 4 is 75mN).

8 Coiling dynamics

To describe the dynamics of the coiling valve, we used a Lagrangian characterisation. The basic setup is depicted in Display item 6. We discretise the valve along its length with N equally spaced points, and track the position in space of discretised points along the endo layer, denoted $\mathbf{r}_i(t) = (x_i(t), y_i(t))$. We orient (for now) the uncoiled valve along the x-axis with the attachment to the plant at the origin and the free tip at $(L, 0)$, and let θ_i be the angle between x-axis and the ith segment (i.e. the line joining $\mathbf{r}_{i-1}$ and $\mathbf{r}_i$). Supposing that the point (x_0, y_0) is fixed at $(0, 0)$ for all time, we have the relations

$$x_i(t) = \tilde{l}\sum_{j=1}^{i}\cos\theta_j(t), \quad y_i(t) = \tilde{l}\sum_{j=1}^{i}\sin\theta_j(t), \tag{29}$$

where

$$\tilde{l} = \frac{L}{N}$$

with L the total length. Since we are tracking the inextensible endo layer, these lengths $\tilde{l}$ do not vary with time. From (29) it follows that

$$\dot{x}_i = -\tilde{l}\sum_{j=1}^{i}\sin\theta_j\dot{\theta}_j, \quad \dot{y}_i = \tilde{l}\sum_{j=1}^{i}\cos\theta_j\dot{\theta}_j. \tag{30}$$

Thus the position and velocity of any point are given in terms of the angles θ_j, and the total kinetic energy

$$T = \sum_{i=1}^{N} m_i(\dot{x}_i^2 + \dot{y}_i^2) \tag{31}$$

can be expressed in terms of the θ_j. Note m_i is the mass of the ith segment; for simplicity we suppose each $m_i = M/N$, where M is the total mass of the valve. The general approach is to write the Lagrangian fully in terms of the variables $\{\theta_1, \theta_2, \ldots \theta_N\}$. For this we next turn to the potential energy.

For the potential energy, we use discretised versions of the expressions for bending and stretching energy derived in Section 1. We make the simplification that the width of the valve does not vary dynamically, in other words we do not explicitly include the flattening and consider the coiling dynamics starting from Config II.

As described in Section 1, the energy can be expressed as a function only of the curvature u, which is well approximated in the discrete setting by the turning angle, that is

$$u(\mathbf{x}_i) \approx u_i := \frac{\theta_{i+1} - \theta_i}{\tilde{l}}, \tag{32}$$

and the discretised potential energy $\mathcal{E}(u)$ is fully expressible in the Lagrangian variables $\{\theta_i\}$ via (32). The formula $\mathcal{E}(u)$ is quite cumbersome due to the nonlinear formulas $d_i(u)$ and $l_i(u)$, hence for computational ease we used a Taylor expansion of the energy for small u in our dynamic calculations. Since the curvature takes a maximum value at approximately $u = 1$, we have kept terms up to u^3 in the expansion, which approximates the energy to within 10^{-3}mJ.

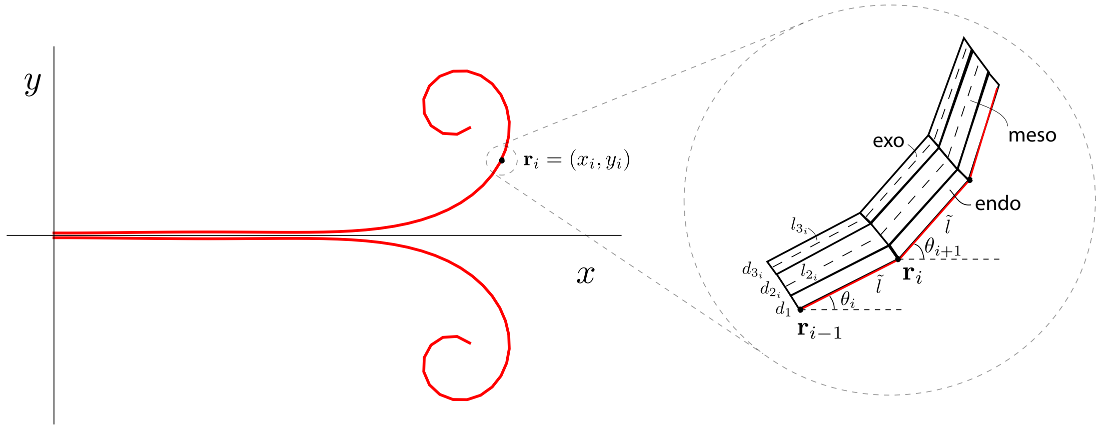

Display item 6: Coiling valve dynamics. Lagrangian setup, geometrical parameters.

8.1 Opposite valve

One further component is needed in the energy to account for the presence of the opposing valve. Both valves are curling away from the plane of the replum in a symmetric manner, and the presence of the other essentially keeps each valve from crossing the replum plane. We model this within the Lagrangian framework by adding an energy term with a large penalty on any point $\mathbf{r}_i$ for which $y_i < 0$, and negligible if $y_i > 0$. We have used the functional form

$$U(y) = \mu\left(\frac{b}{(y-a)^n} - \frac{c}{(y-a)^m}\right) \tag{33}$$

with the values $\mu = 10^{-8}, a = -0.12, b = 0.005125, c = 0.5, n = 5, m = 3$. The extremely small value of μ assures that the energy only has an effect when a y coordinate becomes negative. With this form, the dynamics is generally robust to the specific parameter values. We then add the energy term

$$\mathcal{E}_{\text{rep}} = \sum_{i=1}^{N} U(y_i)\tilde{l}. \tag{34}$$

8.2 Lagrangian

The total potential energy is given by the sum of the above described components, that is

$$V = \mathcal{E}_1 + \mathcal{E}_2 + \mathcal{E}_3 + \mathcal{E}_4. \tag{35}$$

The kinetic energy is given by (31), and the Lagrangian is

$$L = T - V.$$

We note that V is a function of $\{\theta_1, \theta_2, \ldots \theta_N\}$ while T is a function of $\{\theta_1, \theta_2, \ldots \theta_N, \dot{\theta}_1, \dot{\theta}_2, \ldots, \dot{\theta}_N\}$[1] The dynamics is governed by the system of equations

$$\frac{d}{dt}\frac{\partial T}{\partial \dot{\theta}_i} - \frac{\partial T}{\partial \theta_i} + \frac{\partial V}{\partial \theta_i} = 0, \quad i = 1, 2 \ldots, N \tag{36}$$

Along with this we have the initial conditions $\theta_i(0) = \dot{\theta}_i(0) = 0$, for $i = 1, 2 \ldots, N$. This system was integrated forward in time numerically, treated as a system of differential-algebraic equations, as the derivatives cannot be solved for directly (i.e. it cannot be written in the general form $\dot{\mathbf{v}} = f(\mathbf{v})$).

For numerical stability, we have used a clamped boundary condition on the left end, $\theta_1(t) \equiv 0$. Inspection of high speed films of valve coiling suggest that this is a reasonable approximation for the

[1] These functions may be written explicitly, tracing the equations above back to the θ_i, but we do not provide them here as it is not informative to do so.

first portion of the coiling. Thus our model should be able to predict the trajectory of seeds on the half of the valve from the middle to the free tip; seeds on the other half tend to follow a more complicated dynamics, as the valve itself becomes ballistic once the propagating coiling wave reaches the end.

We have also added a damping term, both for numerical stability and to account for energy dissipation. We have taken a simple form proportional to $\dot{\theta}^2$ to account for both air resistance and other forms of dissipation, thus changing (36) to

$$\frac{d}{dt}\frac{\partial T}{\partial \dot{\theta}_i} - \frac{\partial T}{\partial \theta_i} + \frac{\partial V}{\partial \theta_i} + \nu \dot{\theta}_i^2 = 0, \quad i = 1, 2 \ldots, N \tag{37}$$

We have used a small damping coefficient, $\nu = 0.015/N$, which puts the time scale of coiling on the order of 3 ms, consistent with experimental observation. A typical coiling simulation appears in Main text Fig. 1J.

9 Coiling dynamics comparison with experiment

Explosive pod shatter was captured with paired, synchronized, calibrated high-speed cameras (see Material and Methods section). A calibration plane was placed in different orientations throughout the volume of view and used in a photogrammetric technique known as direct linear transformation to create a calibration matrix that transformed x and y pixel positions from each camera into x, y, z coordinates in space. Further details of the calibration procedure are available elsewhere Walker et al. (2008). Identifiable locations along the valve and the ejected seeds were tracked until they left the fields of view. Positional data acquired at 15,000 frames per second were filtered using a 3rd order Butterworth filter with a cut off frequency of 3000 Hz.

The result of the data extraction is multiple sets of points in 3-space, each set of the form

$$\{(x_1, y_1, z_1), (x_2, y_2, z_2), \ldots, (x_n, y_n, z_n)\},$$

tracking the location of a particular material point at each time step. To convert the data to a form comparable with the Lagrangian model, we first converted this data to a planar form by determining the plane of best fit and projecting all data points on the plane. We then rotated all data points to orient the valve along the x-axis. To mimic the experiment with the Lagrangian model, we chose material points corresponding to the same locations along the valve as the data and plotted the location of each material point at equivalent time steps (Main text Fig. 1K).

9.1 Seed release

The Lagrangian dynamics provide the accelerations and velocities for any material point on the valve. To investigate the distribution of seeds, we require, for a seed at a given location on the valve, the launch velocity and ballistic trajectory. This requires first a criterion for a seed to be be ejected from the valve.

9.2 Seed ejection criterion

The mechanism for seed ejection is quite complex, as it depends on the exact nature of the adhesion between the seed and the valve, the changing geometry of the coiling valve, the air flow past the valve/seed, and the accelerations, which vary both with time for any given seed, and over the length of the valve. It is not our intention here to go into great detail in modelling the exact nature of the seed to valve bond. Rather, we take a data driven approach. The Lagrangian dynamics give access to the accelerations and velocities at all material points. From these, we consider three hypotheses, and examine the feasibility of each in the context of experimental observables, namely seed distribution and observations of launch angles.

Maximum speed release. First, we examine the idea that each seed is launched at the point when its speed is maximal. While it is forces that ultimately cause seed release, not velocities, this hypothesis is nevertheless worth consideration as this is the criterion for each individual seed to travel as far as possible. It is conceivable that the plant could have devised a mechanism for release to occur at this point, for instance by having differential adhesion properties along the length of the valve. However, from the coiling dynamics, we find that maximal speed occurs almost simultaneously with the velocity pointing in the direction tangent to the valve itself, and with a roughly 45° launch angle to the valve.

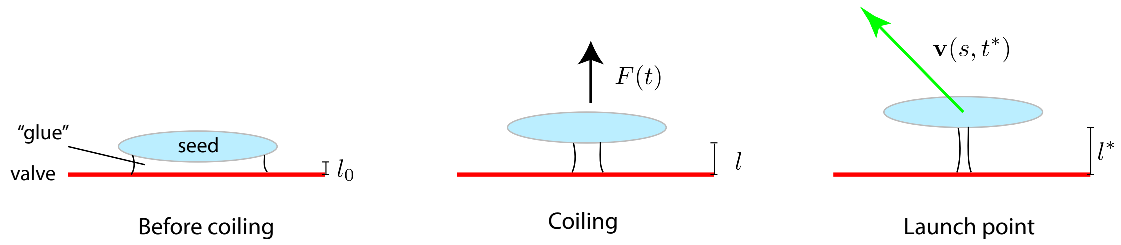

Display item 7: Schematic for the viscoelastic seed release model. During coiling the seed feels a force due to the acceleration of the valve; this deforms the viscoelastic "glue"; the seed launches when its strain crosses a threshold value; the initial velocity and position of the seed are then taken to be that of the valve when the threshold is crossed.

Therefore, under this hypothesis all seeds launch with roughly the same angle (Display Item 8(a)). As this does not match experimental observation (Display item 8(c)), we discard this hypothesis.

Critical force hypothesis The simplest physics-based hypothesis is that seeds at all points along the valve have a similar adhesive bond, and that the seed departs from the valve when the force due to acceleration of the valve passes a threshold value. This hypothesis is problematic for two reasons: first the seeds towards the free tip of the valve feel accelerations on the order of 10 times that of seeds towards the middle, and second, the acceleration at any given material point is maximal immediately after that point begins coiling. Under this hypothesis the seeds that eject at all would eject at an angle directly orthogonal to the axis of the valve, which is also not the case.

Viscoelastic adhesion hypothesis A small but important extension to the notion of a critical force is to allow the forces felt by the seed to integrate over time and ultimately lead to ejection. We utilised a simple Kelvin-Voigt viscoelastic spring model to investigate this hypothesis. We suppose that in its rest state, the seed is connected to the valve by a coat of "glue" of thickness l_0. Letting $l(t)$ denote the total length of the glue at time t, and defining

$$\epsilon := \frac{l}{l_0},$$

which measures the strain of the viscoelastic glue (and the distance the seed has pulled away from the valve), we have

$$\eta\epsilon'(t) + E(\epsilon - 1) = \sigma(t), \tag{38}$$

where η is the viscosity and E the Young's modulus of the glue and σ is the stress in the material. At time t, the seed at material point s along the valve is subject to the force

$$\mathbf{F}(t) = m\mathbf{a}(s,t) \tag{39}$$

where m is the mass of the seed and $\mathbf{a}$ the acceleration vector determined from the Lagrangian dynamics. In the viscoelastic spring model, the stress σ is given by $|\mathbf{F}|/A$, where A is the cross-sectional area; assuming a cylindrical column of glue with fixed volume V, we have

$$A = \frac{V}{l} = \frac{V}{\epsilon l_0}.$$

The launch criteria is then given by defining a threshold strain ϵ^*, or equivalently a seed-to-valve distance l^*, at which the glue has thinned to the point of breaking. At this point the seed becomes ballistic with an initial velocity equivalent to the material velocity $\mathbf{v}(s,t^*)$, where $\epsilon(t^*) = \epsilon^*$.

A full analysis requires measuring of the amount and rheological properties of the glue, as well as determining a critical strain. We leave such details for a future study; the objective here is to investigate this adhesion hypothesis by testing the qualitative effect on the ballistic launch and eventual distribution of seeds. For this, we estimated the viscoelastic and geometric parameters (Display Table 11). Viscoelastic parameters were estimated from previous seed mucilage rheology study Deng et al. (2013). For a seed at a given material point, we used the output of the Lagrangian model to integrate forward Eqn (38). The point at the free tip is subject to the largest accelerations and hence highest strain, hence we used this point to set the threshold strain. We then integrate (38) forward for material

Parameter	Description	Value
η	viscosity	50 Pa·s
E	modulus of elasticity	500 Pa
l_0	initial glue thickness	5 μm
V	glue volume	$6.2 \cdot 10^{-4}$ mm^3
m	seed mass	0.11 mg

Display Table 11: Parameter values for viscoelastic model.

points from the free tip to around the midpoint of the valve[2]; at each point, once we have determined the time at which the threshold strain is reached, we pass the position and velocity at that time to a calculation of the ballistic trajectory of the seed.

Display item 8(b) shows the launch point and velocity for 10 seeds under the viscoelastic release hypothesis. We find that the seeds launch with a distribution of velocities and angle: the seeds at the free tip launch nearly perpendicularly to the initial valve axis and with a large velocity; the seeds further away, subject to smaller forces, hold on to the valve for a longer time before reaching the threshold strain and thus launch in a direction more parallel to the valve axis. This general behaviour was confirmed in seed dispersal videos in which launch angles were evident as blurred lines, see Display item 8(c).

9.3 Seed distribution

We now turn to the largest scale we shall consider: that of seed dispersal. In particular, the probability density function of the seeds landing points around the plant is of central importance in understanding the advantage of the ballistic mechanism of *Cardamine hirsuta*.

The models of Secs. 8 & 9 provide the velocity and position of seeds as they leave a seed pod in a frame of reference attached to the pod. To infer the seed distribution, both the distance between the seed pod and the ground and its orientation with respect to the vertical must be taken into account. The latter can be specified by the three angles $\alpha \in [0, \pi/2]$, the angle to the vertical, $\psi \in [0, 2\pi)$, the rotation about the vertical axis, and $\phi \in [0, \pi)$, the rotation about the valve axis, as shown on Display item 9. Since the angle ψ does not influence how far the seeds land, we fix $\psi = 0$ for all computations.

More specifically, we define $\mathbf{d}_a$ the unit vector along the length of the pod and $\mathbf{d}_b$, the unit vector perpendicular to the plane of the replum, and denote s the arc-length along the valve. The model of Sec. 9 provides the components $\begin{pmatrix} \mathsf{v}_a & \mathsf{v}_b \end{pmatrix}^T$ of the velocity vector $\mathbf{v}$ of the seeds at launch as a function of s and in the planar basis $\{\mathbf{d}_a, \mathbf{d}_b\}$:

$$\mathbf{v}(s) = \mathsf{v}_a(s)\,\mathbf{d}_a + \mathsf{v}_b(s)\,\mathbf{d}_b. \tag{40}$$

Then the components $\begin{pmatrix} \mathsf{v}_x & \mathsf{v}_y & \mathsf{v}_z \end{pmatrix}^T$ of the velocity in the fixed orthogonal basis (lab frame) $\{\mathbf{e}_x, \mathbf{e}_y, \mathbf{e}_z\}$ (see Display item 9) are given by

$$\begin{pmatrix} \mathsf{v}_x \\ \mathsf{v}_y \\ \mathsf{v}_z \end{pmatrix} = \begin{pmatrix} \cos(\alpha + \pi/2) & 0 & \sin(\alpha + \pi/2) \\ 0 & 1 & 0 \\ -\sin(\alpha + \pi/2) & 0 & \cos(\alpha + \pi/2) \end{pmatrix} \begin{pmatrix} 1 & 0 \\ 0 & -\sin\phi \\ 0 & \cos\phi \end{pmatrix} \begin{pmatrix} \mathsf{v}_a \\ \mathsf{v}_b \end{pmatrix}. \tag{41}$$

To take into account the fact that each pod has two valves, for each seed launched with velocity $\mathbf{v}$ defined by Eq. (41), there is also a seed launched with velocity $\mathbf{v}^\dagger$:

$$\begin{pmatrix} \mathsf{v}_x^\dagger \\ \mathsf{v}_y^\dagger \\ \mathsf{v}_z^\dagger \end{pmatrix} = \begin{pmatrix} \cos(\alpha + \pi/2) & 0 & \sin(\alpha + \pi/2) \\ 0 & 1 & 0 \\ -\sin(\alpha + \pi/2) & 0 & \cos(\alpha + \pi/2) \end{pmatrix} \begin{pmatrix} 1 & 0 \\ 0 & -\sin(\phi + \pi) \\ 0 & \cos(\phi + \pi) \end{pmatrix} \begin{pmatrix} \mathsf{v}_a \\ \mathsf{v}_b \end{pmatrix}. \tag{42}$$

Given the initial position and velocity of the seeds at launch from the valve, their trajectory can be computed to determine their landing point. To this end, a seed is tracked by its position vector $\mathbf{r}(t)$ in the frame $\{\mathbf{e}_x, \mathbf{e}_y, \mathbf{e}_z\}$. The forces acting on it are the gravitational force: $-mg\,\mathbf{e}_z$ and the drag force F_d given by the drag equation $F_d = -c_d\, A\, \rho_{\text{air}} |\mathbf{r}'|\, \mathbf{r}'$ where $m = 1.1\, 10^{-7}$ kg is the mass of the seed, $g = 9.81\,\text{m/s}^2$ is the nominal gravitational acceleration, $c_d \simeq 0.9$ is the shape dependent drag coefficient

[2] As stated earlier, the Lagrangian model is only well suited for this range of the valve, due to the use of a clamped boundary condition. In the latter half of the valve, the dynamics are far more complicated and varied, since the valve itself often (though not always) becomes ballistic.

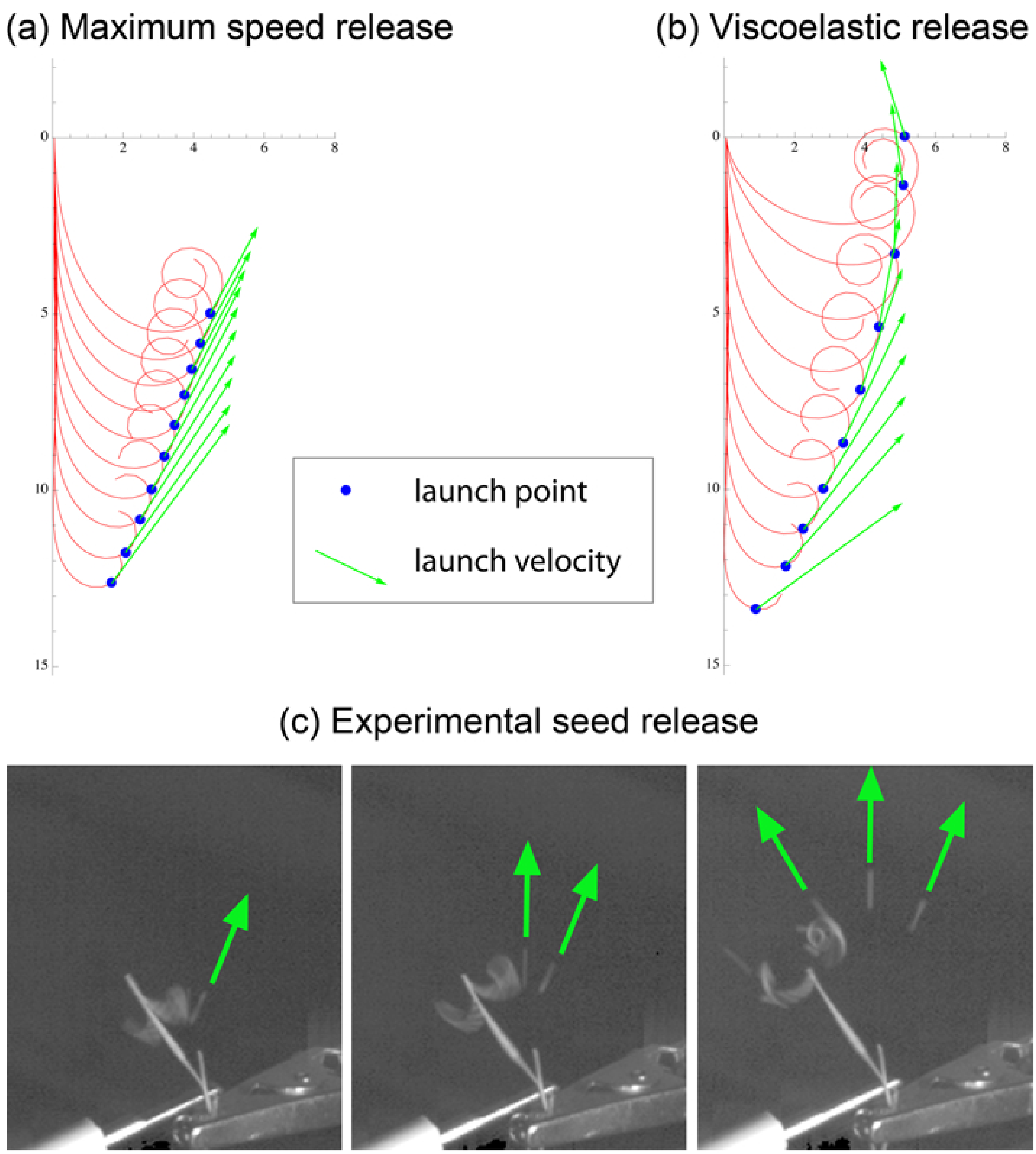

Display item 8: Launch angle and velocity under different seed release hypotheses: maximum speed hypothesis (a) and viscoelastic glue hypothesis (b). (c): Single frame of explosive seed dispersal, recorded at 1,500 fps; each seed is a blurred line, indicating the distance moved in one frame (0.67 ms); launch vectors of three successively launched seeds are shown.

of a streamlined body, $A \simeq 0.5\,\mathrm{mm}^2$ is the average cross-sectional area (perpendicular to the direction of flight) of the seed, $\rho_{\mathrm{air}} = 1.2\,\mathrm{kg/m}^3$ is the mass density of the air and $'$ denotes time derivatives. Newton's second law of motion therefore reads

$$\mathbf{r}'' = -g\,\mathbf{e}_z - \frac{c_d\,A\,\rho_{\mathrm{air}}}{m}|\mathbf{r}'|\,\mathbf{r}'. \tag{43}$$

Finally the distribution of seed landing points around the plants can be estimated by a Monte Carlo computation. The angle α between the longitudinal axes of the pod $\mathbf{d}_1$ and the vertical $\mathbf{e}_z$ was measured for 108 fruits. We found that the distribution can be modelled as Gaussian with an estimated 37.6° mean and 5.1° standard deviation. We could not obtain an explicit distribution for the angle ϕ, but we performed bimodal observations on 24 plants: for each valve on each plant it was assessed whether ϕ was closer to zero or $\pi/2$. The majority (473 out of 504 valves) displayed an orientation closer to $\phi = 90°$, hence we have taken ϕ to be Gaussian distributed with mean 90° and standard deviation 31.5°. The Monte Carlo simulation proceeds by choosing a valve orientation from the assumed distributions, then computing the trajectory, in particular the landing distance from the plant, of each seed. In this way, for given initial seed velocities we produce a probability density function (pdf) of the landing distance of the seeds.

The landing distance pdf for the baseline valve parameters listed in the Display tables in this SOM text is shown in the Main text Fig. 1E, along with the experimental data. It is important to note that in the model, trajectories were only computed for seeds from the free tip to the midpoint of the valve, due to the coiling and ballistic complexities mentioned earlier for seeds on the other half of the valve. In producing Main text Fig. 1E, we have thus scaled the height of the distribution down by a factor of 2 to account for the missing seeds, hence this pdf only adds to 0.5 when taken over all bins. The experimental data, on the other hand, includes seeds from the entire length of each valve, and we would thus expect the model to predict a subset of the experimental data. This is found to be the case, with the primary difference between model and data being that the data showed close to half of the seeds landing within 0.5 m of the plant, while the model predicts only a very small fraction in this range. Nevertheless, this fact is consistent with the general trend we observe in seed trajectories: while we do not explicitly model the remaining seeds, we can surmise that they would land closer to the plant than those from the free tip half of the valve, because the launch speed is generally highest for the seed at the free tip and decreases as the distance from the free tip increases. Hence the seeds furthest from the free tip will launch with the lowest initial speed.

Moreover, we remark that we have only assumed a distribution on the valve orientations. In reality, there are many parameters that may vary from valve to valve (and from plant to plant) that will affect the overall distribution of seeds. While it is impractical to exhaustively vary or provide distributions for all parameters, we assessed the sensitivity to two key factors: the drag coefficient, and the mechanical energy built up in each valve. The former can vary significantly based on whether the seed travels in a tumbling motion or a more aerodynamically favourable "frisbee" type motion. This is hard to ascertain since high speed films can only capture the first couple milliseconds of flight with high resolution before the seeds leave the field of view. Rather than delve into the specifics of the aerodynamics, we assessed the sensitivity to drag by simulating the base values for initial velocity with a drag coefficient reduced by 30%. To assess the sensitivity to mechanical energy, we simulated a low energy and high energy valve coiling, by changing the degree of exocarp length decrease, while keeping all other parameters fixed. That is, we varied the differential shrinkage factor g from the base value 0.8 to 0.75 (increased energy) and 0.85 (decreased energy). We then computed launch velocities and ran Monte Carlo simulations to compute the landing pdf for each case. The result is plotted in Display item 10, which shows the pdf for the base values, reduced energy, increased energy, and reduced drag cases. This shows a predictable shift: the higher energy valves result in seeds landing further from the plant, the lower energy valves land closer, and the reduced drag seeds land the furthest. In all cases, the computed pdf remains within the range of the experimental data. These simulations, when taken to be a lumped representation of the variation across different valves, suggest that the wide distribution of seeds landing observed in the wild type fruit comes from differential launch velocities along the valve, combined with a variation in energy, fruit orientation, and possibly seed aerodynamics.

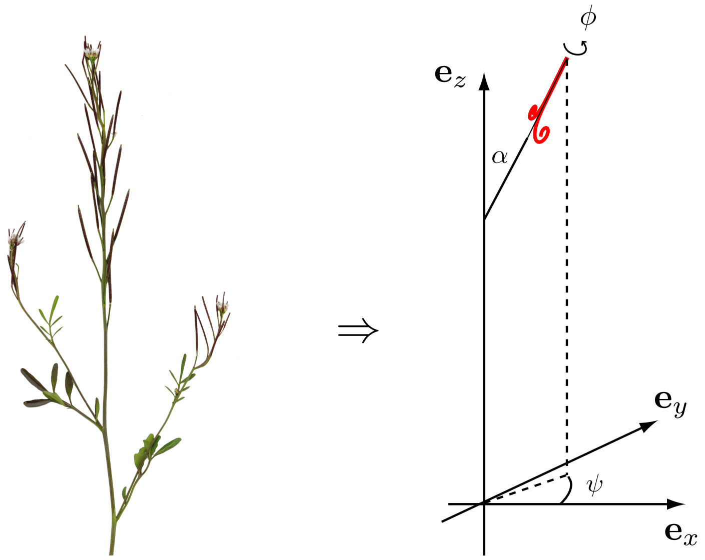

Display item 9: Schematic of the valve geometry for coiling and seed launch. The orientation of the valve is determined by the angles α, ϕ, and ψ, which measure the angle from the vertical, the rotation about the axis of the valve, and the rotation about the vertical direction, respectively.

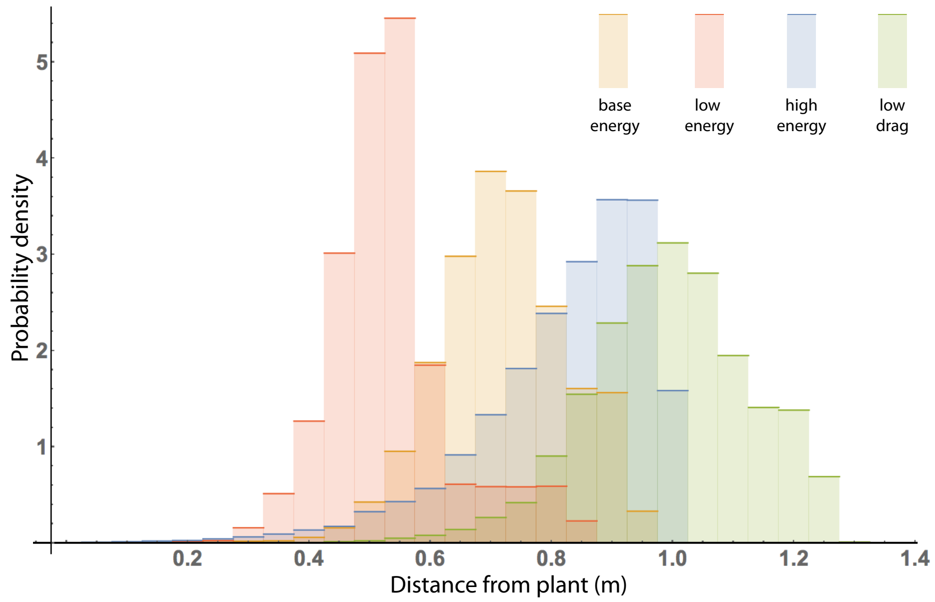

Display item 10: Simulated seed distributions for varying valve energy and drag. Base case ($g = 0.80$ and $c_d = 0.9$) together with decreased potential energy ($g = 0.85$), increased potential energy ($g = 0.75$) and decreased drag ($c_d = 0.6$).

	Explosive pod shatter			Osmotic treatment
	Ratio pre- to post-shatter			Ratio 1M NaCl to water
	Wild type		*lig2*	Wild type
Cell geometry	Whole valve	Minus endocarp	Whole valve	Whole valve
Length	0.80 ± 0.004	0.85 ± 0.002	0.89 ± 0.002	0.87 ± 0.010
Width	1.01 ± 0.004	0.96 ± 0.003	1.02 ± 0.002	1.18 ± 0.012
Depth	1.41 ± 0.010	1.48 ± 0.007	1.07 ± 0.007	1.39 ± 0.021
Surface area	0.99 ± 0.002	1.03 ± 0.003	0.97 ± 0.003	
Volume				1.53 ± 0.025

Table S1. *C. hirsuta* Exocarp Cell Deformations, Related to Figure 5. Exocarp cell deformations shown as ratios; error is standard error of the mean ratio. Exocarp cells were imaged on the whole fruit pre-shatter, and short segments of either the whole valve, or the whole valve dissected off from the endocarp, were imaged post-shatter. This turgor response of exocarp cells does not require a bilayer, as similar cell deformations were observed in *lig2* mutants, and in wild-type valves consisting only of outer layers (minus endocarp). $N = \leq 219$ cells per measurement.

Name	Oligo sequence	Reference
pNST3-F	GGGGACAACTTTGTATAGAAAAGTTGTTggtaattacgtcagtatgagcatgggttt	ChpNST3-fg
pNST3-R	GGGGACTGCTTTTTTGTACAAACTTGTtgatgatgatggcgatatctttttggtcct	ChpNST3-Rg
pLIG2-F	GGGGACAACTTTGTATAGAAAAGTTGTTctcttcttcactgtcacttttgctt	CHpBIN4F
pLIG2-R	GGGGACTGCTTTTTTGTACAAACTTGTaattcacttcttcagagctcaaaac	CHpBIN4R
gLIG2-F1	GGGGACAAGTTTGTACAAAAAAGCAGGCTTTatgagcagctctagagaggaatctcc	CHgBIN4F
gLIG2-R1	GGGGACCACTTTGTACAAGAAAGCTGGGTTtttcttggtttttggcttcttaggagc	CHgBIN4R
glig2-R1	GGGGACCACTTTGTACAAGAAAGCTGGGTTatcatctggctttgaaccagtcttg	CHgBIN4Rdelta
qLIG2-F3	GCGATTATAGCCGAGGAAGTAACAACG	hfe1_qrtBIN4f1
qLIG2-R3	CAAGTCCAAGTACATGTCCCCGG	hfe2_qrtBIN4r1
AP2M-F	TCGATTGCTTGGTTTGGAAGATAAGA	qPCR AP2M FW
AP2M-R	TTCTCTCCCATTGTTGAGATCAACTC	qPCR AP2M RV
LIG2-F2	CCACAATCTAATGTCTATGAGGCAGA	SNP15514471F_BclI
YFP-R	TTACGTCGCCGTCCAGCTCGAC	Venus-r
ACT8-F	AGCTCCGTATTGCTCCTGAA	NB80_Ch ACT8-F
ACT8-R	CAGTGAGGTCACGACCAGCA	SH843_Ch ACT8-R
mapping	TTCCATTTGTAGAAGAACAAGTG	14637-F
mapping	GGTTGCTCAGAACATGAAAGATGGC	14637-R
mapping	GGCTGTTGGGCTAAGCTATGCAAC	14546-F
mapping	ATATCATTATTCTCTACACTCC	14546-R
mapping	ACCATACCAAAGAACAATAACATG	SNP_22377-F
mapping	AAGCGGTTTGATAAGTACGTAACTG	SNP_22377-R
mapping	TGTTTAGAGATTTCTCCCTCACC	14528-F
mapping	TTGAAGACTGGTATCAGAAGCATT	14528-R
mapping	AAACACAACTCCCATCACAATC	640-F
mapping	CGTTTTGATTCGCATCTTCA	640-R
mapping	TGGTTTGTGTCTGGTTTCATAA	210-F
mapping	TTGCAAATCTACAGGAATCTTCA	210-R
mapping	ATGAACCCAGAAACCAAGAATG	853_1F
mapping	TTGTTTTTGGTGGGTCTCTTCT	853_1-R
mapping	TATTAAGTTGGGGTTTTGTAAGA	15339098-F

mapping	CTCAAACTTGAAAAACATTTGAACC	15339098-R
mapping	AGAGTTGAGACATCAAACAGT	15400849-F
mapping	CCCCTCAAAACAATATACGTAA	15400849-R
mapping	ATGTGAAAGTCACCGTAATTCT	15421459-F
mapping	TGTTTCTTTAAGTCTTTGGCTAAT	15421459-R
mapping	TACATCAACATTAGCATATATGT	15429314-F
mapping	AAAACAACAATAAGAAAAACTAAAA	15429314-R
mapping	GATATCGAACTTGCAATATTTCAT	15438646-F
mapping	AATGATCAAACATTTGACACATGG	15438646-R
mapping	GGAAGCTGCACTAGTAGTAAGA	15445290-F
mapping	CTCTCACAGCTTCTGAGACTCCAT	15445290-R
mapping	AAGCTGCACTAGTAGTAAGAAAAT	15463003-F
mapping	CTTCTGAGACTCCATCCTGCAAT	15463003-R
mapping	ATATCTTTGGTTAATTCTCATCCA	15483622-F
mapping	CTGTAATGATGAGATGTGAAAGG	15483622-R
mapping	TTCTCCCATGAATTAAAGAGGTG	15539723-F
mapping	GTACAAACATAATGTTTAAATAT	15539723-R
mapping	AAGTAAGAAATTTTGTTTTGTT	15561005-F
mapping	ACCACCAAAATGAAAAAAAAGTTT	15561005-R

Table S2. Oligonucleotides Used in This Study, Related to Experimental Procedures.

Supplemental References

Bennett, T., van den Toorn, A., Sanchez-Perez, G.F., Campilho, A., Willemsen, V., Snel, B., and Scheres, B. (2010). SOMBRERO, BEARSKIN1, and BEARSKIN2 regulate root cap maturation in Arabidopsis. Plant Cell *22*, 640-654.

Bonet, J., Burton, A. (1998). Computer Methods in Applied Mechanics and Engineering, 162, 151.

Chapelle, D., Bathe, K. J. (2003). The finite element analysis of shells: fundamentals. In computational fluid and solid mechanics, (Springer, Berlin, New York) Sur la p. de titre: with 81 figures.

Deng, W., Iannetta, P.P., Hallett, P.D., Toorop, P.E., Squire, G.R., and Jeng, D.S. (2013). The rheological properties of the seed coat mucilage of *Capsella bursa-pastoris* L. Medik. (shepherd's purse). Biorheology 50, 57-67

Federl, P., Prusinkiewicz, P. (1999). Proceedings of Computer Graphics International,

Galinha, C., Hofhuis, H., Luijten, M., Willemsen, V., Blilou, I., Heidstra, R., and Scheres, B. (2007). PLETHORA proteins as dose-dependent master regulators of Arabidopsis root development. Nature *449*, 1053-1057.

Gan, X., Stegle, O., Behr, J., Steffen, J.G., Drewe, P., Hildebrand, K.L., Lyngsoe, R., Schultheiss, S.J., Osborne, E.J., Sreedharan, V.T., *et al.* (2011). Multiple reference genomes and transcriptomes for *Arabidopsis thaliana*. Nature *477*, 419-423.

Holzapfel, G. A. (2000). Nonlinear Solid Mechanics. A continuum approach for engineering, (J. Wiley and Sons, Chichester).

Lessinnes, T., Moulton, D. E., Goriely, A. (2015). Journal of the Mechanics and Physics of Solids.

Pfaffl, M.W. (2001). A new mathematical model for relative quantification in real-time RT-PCR. Nucleic Acids Res *29*, e45.

Prasad, K., Grigg, S.P., Barkoulas, M., Yadav, R.K., Sanchez-Perez, G.F., Pinon, V., Blilou, I., Hofhuis, H., Dhonukshe, P., Galinha, C., *et al.* (2011). Arabidopsis PLETHORA transcription factors control phyllotaxis. Curr Biol *21*, 1123-1128.

Smith, C., Prusinkiewicz, P., Samavati, F. (2004). Applications of Graph Transformations with Industrial Relevance. In Second International Workshop, AGTIVE 2003, J. L. Pfaltz, M. Nagl, B. Blen, eds. (Springer-Verlag GmbH), pp. 313-327.
